# Small RNA and degradome profiling reveals miRNA regulation in the seed germination of ancient eudicot *Nelumbo nucifera*

**DOI:** 10.1186/s12864-016-3032-4

**Published:** 2016-08-26

**Authors:** Jihong Hu, Jing Jin, Qian Qian, Keke Huang, Yi Ding

**Affiliations:** 1 State Key Laboratory of Hybrid Rice, College of Life Sciences, Wuhan University, Wuhan, 430072 China; 2 State Key Laboratory of Genetic Resources and Evolution, Kunming Institute of Zoology, Chinese Academy of Sciences, Kunming, 650223 China

**Keywords:** miRNA, Target genes, Degradome sequencing, Quantitative qRT-PCR, *Nelumbo nucifera*

## Abstract

**Background:**

MicroRNAs (miRNAs) play important roles in plant growth and development. MiRNAs and their targets have been widely studied in model plants, but limited knowledge is available concerning this small RNA population and their targets in sacred lotus (*Nelumbo nucifera* Gaertn.).

**Results:**

In this study, a total of 145 known miRNAs belonging to 47 families and 78 novel miRNAs were identified during seed germination using high-throughput small RNA sequencing. Furthermore, some miRNA families which have not yet been reported in monocot or eudicot species were detected in *N. nucifera*, indicating that these miRNAs was divergence from monocots and core eudicots during evolution. Using degradome sequencing, 2580 targets were detected for all the miRNAs. GO (Gene Ontology) and KEGG pathway analyses showed that many target genes enriched in “regulation of transcription” and involved in “carbohydrate”, “amino acid and energy metabolism”. Nine miRNAs and three corresponding targets of them were further validated by quantitative RT-PCR.

**Conclusions:**

The results present here suggested that many miRNAs were involved in the regulation of seed germination of sacred lotus, providing a foundation for future studies of sacred lotus seed longevity. Comparative analysis of miRNAs from different plants also provided insight into the evolutionary gains and losses of miRNAs in plants.

**Electronic supplementary material:**

The online version of this article (doi:10.1186/s12864-016-3032-4) contains supplementary material, which is available to authorized users.

## Background

MicroRNAs (miRNAs) are approximately 21 nucleotides endogenous non-coding RNAs that play important roles in the regulation of gene expression at the post-transcriptional level, resulting in the cleavage or translation repression of target mRNAs [1, 2]. In plants, miRNA-guided gene regulation is involved in multiple developmental processes, such as floral development [3], male and female sterility [4, 5], leaf growth [6], and RNA metabolism [7]. Most plant miRNAs have perfect or near-perfect sequences complementary with their target mRNA to regulate gene expression at the post-transcriptional level [8]. Thus, identifying miRNAs and their targets, and elucidating their regulatory mechanisms are critical to understand the development processes.

Recently, degradome sequencing has been successfully used to analyse the miRNA targets. Combined with small RNA sequencing, the pace of miRNA and their targets identification have been greatly improved in plants [9]. Other studies have also suggested that whole-scale miRNA identification and targets analysis might reveal regulatory networks in plant growth and development [10]. Lately, degradome sequencing has been used to find target genes on Arabidopsis, rice, cotton and so on [11–13]. However, the miRNA targets in sacred lotus (*Nelumbo nucifera* Gaertn.) have only been predicted by bioinformatics method, and little information has been experimentally validated [14, 15].

Sacred lotus is a perennial aquatic plant with ornamental, edible, medicinal value and phylogenetic importance [16]. In Asia, sacred lotus has been cultivated for more than 7000 years due to its beautiful flowers, edible rhizomes and seeds [17, 18]. Recently, the genome sequences of the sacred lotus variety ‘China Antique’ have been sequenced, which is available for further identifying miRNAs in *N. nucifera* [19, 20].

As an ancient basal eudicots, sacred lotus seed is one of the oldest directly dated seeds for ~1300 year-old. Previous studies reported that 16 old fruit of ‘China Antique’ were collected from the same lakebed and 84 % of them can be germinated in ~3 days [21, 22]. The proteins which were heat stable and ROS removal were reported to be important for seed longevity [21–23]. ROS adversely affects cellular proteins and enzymes and renders them inactive. Some thermal proteins, including superoxidae dismutase (SOD) and HSP were demonstrated to reduce deleterious ROS accumulation for improving the seed longevity [22, 23]. Because of the sacred lotus seeds remain viable after hundreds of years, resistance and damage repair in seeds must be extraordinarily effective [24]. Therefore, sacred lotus seeds could be an excellent model for studying the adaptive mechanisms of seeds [25].

Seed germination is the first step for plant growth and plays a pivotal role in seedling establishment. It is a complex physiological and biochemical process and previous studies suggested a role for miRNAs in seed germination [26–29]. Using small RNA sequencing, the miRNA expression patterns in rice embryos at 0, 12 and 24 h after imbibition showed that osa-miR159f, osa-miR166l and osa-319b were predominantly expressed in rice seeds [27]. In soybean, gma-miR1530 and gma-miR1536 have been implicated in the conversion between photosynthesis and lipid accumulation by regulating their target genes of transketolase and carboxylase, respcetively [28]. In maize, 115 know miRNAs were identified in the seeds at 24 h after imbibition, and the regulation of the genes targeted by these miRNAs was involved in the early stage of seed germination [29]. However, little miRNAs were known about the seed germination of sacred lotus. Therefore, identification of novel miRNAs and elucidation of their functions in the seed germination will help us to understand the long living of sacred lotus seed. Furthermore, as a basal eudicot, identification of the sacred lotus miRNA and comparison with other angiosperms will aid the study of conservation and evolution of miRNAs in plants.

To comprehensively investigate small RNAs and their targets and provide some information for further understanding the miRNA-mediated regulation network in the seed germination of sacred lotus, five small RNA libraries and a degradome library were constructed in this study. The profiling of the miRNAs and their target genes provides some insights into the regulatory pathways governing gene expression during the seed germination, and the data presented here will lay a foundation for future studies of sacred lotus seed longevity.

## Results

### Overview of small RNA library sequencing

To elucidate the regulatory roles of miRNAs during the seed germination of sacred lotus, five small RNA libraries were constructed from germinating seeds at 0 h, 12 h, 24 h, 36 h and 72 h after imbibition and then sequenced by a Hiseq2,500 platform. After filtering the low-quality reads, including reads <18 nt in length, 5′ adaptor contaminates, reads with polyA, and reads without 3′adaptors, ranging from 10,245,283 clean reads (at 72 h) to 14,412,672 clean reads (at 0 h) were obtained. More than 60 % of the clean reads were successfully mapped to the sacred lotus reference genome (Table 1). Moreover, 3,347,363, 4,084,098, 3,575,988, 4,002,600 and 3,497,201 clean reads were unique mapped to the genome. Other unannotated unique sequences were used for novel miRNAs prediction and further analysis (Table 1).

**Table 1:** Data set summary of sequencing of the five small RNA and one degradome libraries

		0 h	12 h	24 h	36 h	72 h
Small RNA data	Clean reads	14,412,672	14,045,040	10,736,476	12,983,765	10,245,283
	Mapped reads	9,362,670(64.96 %)	8,479,342(60.37 %)	6,700,319(62.41 %)	8,378,691(64.53 %)	6,474,276(63.19 %)
	Unique mapped	3,347,363(100)	4,084,098(100)	3,575,988(100)	4,002,600(100)	3,497,201(100)
	Match miRNA	3,347(0.10 %)	5,309 (0.13 %)	4,648(0.13 %)	4,803(0.12 %)	3,847(0.11 %)
	Unannotation	3,047,439(91.04 %)	3,725,514(91.02 %)	3,248,070(90.83 %)	3,633,160(90.77 %)	3,152,027(90.13 %)
		Total	Unique			
Degradome data	Clean Reads	13,852,945(100)	4,362,200(100)			
	Mapped reads	13,787,820(99.53 %)	4,333,854(99.35 %)			
	Transcript Mapped	7,659,047(55.29 %)	2,018,752(46.28 %)			
	Unannotation	6,193,898(44.71 %)	2,343448(53.72 %)			

In all of the five libraries, the small RNA (sRNA) sequences were ranged from 18 to 28 nt in length, with the majority were 19 to 24 nt. And the 24 nt sRNAs were the most abundant within the total sRNA, which approximately accounted from 20 % to 35 % in all of the five sRNA libraries, followed by 21 nt sRNAs, the typical length of canonical miRNAs (Additional file 1: Figure S1).

The common and specific of total and unique small RNA sequences were compared between the adjacent libraries (0 vs12h, 12 h vs 24 h, 24 h vs 36 h and 36 h vs 72 h), showing that more than 75 % of the total sRNAs were shared (Additional file 1: Figure S2). However, only a small fraction (approximately 12.5 % ~ 14 %) of unique sRNA shared in adjacent libraries, suggesting that these small RNAs were low in abundance but highly in diverse.

### Identification of miRNAs and compared with other plants

Conserved miRNA families have been identified in many plant species and have important functions in plant development [29]. In the present study, 145 known miRNAs (belonging to 47 families) were identified (Table 2 and Additional file 2: Table S1). The unannotated small RNA tags were used to predict novel miRNAs and their secondary structures of the precursors were further detected by MFOLD or RNAfold to remove the false positive (Additional file 1: Figure S3). Finally, 78 novel miRNAs were identified in sacred lotus (Additional file 1: Figure S3, Additional file 2: Table S2). The largest miRNA family size identified was Nnu-miR396 comprising 15 members, following by Nnu-miR169 and Nnu-miR393 possessed 12 and 8 members, respectively. Other miRNA families such as Nnu-miR397, Nnu-miR403, Nnu-miR529 and Nnu-miR827 possessed only one member detected in sacred lotus during seed germination of sacred lotus (Table 2 and Additional file 2: Table S1).

**Table 2:** Comparion of the miRNA families identified in Arabidopsis, rice, Amborella and sacred lotus (*Nelumbo nucifera*)

Family	Arabidopsis	Rice	Amborella	Sacred lotus
miR156	10	12	4	5
miR157	4	0	0	4
miR159	3	6	1	3
miR160	3	6	1	3
miR162	2	2	0	3
miR164	3	6	2	4
miR165	2	0	0	2
miR166	7	13	4	4
miR167	4	10	1	4
miR168	2	2	1	3
miR169	14	18	3	12
miR170	1	0	0	3
miR171	3	9	3	7
miR172	5	4	1	7
miR319	3	2	5	4
miR390	2	1	1	2
miR393	2	2	1	8
miR394	2	1	1	3
miR395	6	25	1	2
miR396	2	8	5	15
miR397	2	2	2	1
miR398	3	2	1	3
miR399	6	11	0	6
miR403	1	0	0	1
miR408	1	1	0	6
miR529	0	2	0	1
miR530	0	1	0	2
miR535	0	1	1	1
miR827	1	1	0	1
miR828	1	0	1	1
miR837	1	0	0	1
miR1432	0	1	0	1
miR2111	2	0	1	2
miR2118	0	17	0	2
miR2275	0	4	0	2
miR2950	0	0	1	2

**Fig. 1:**
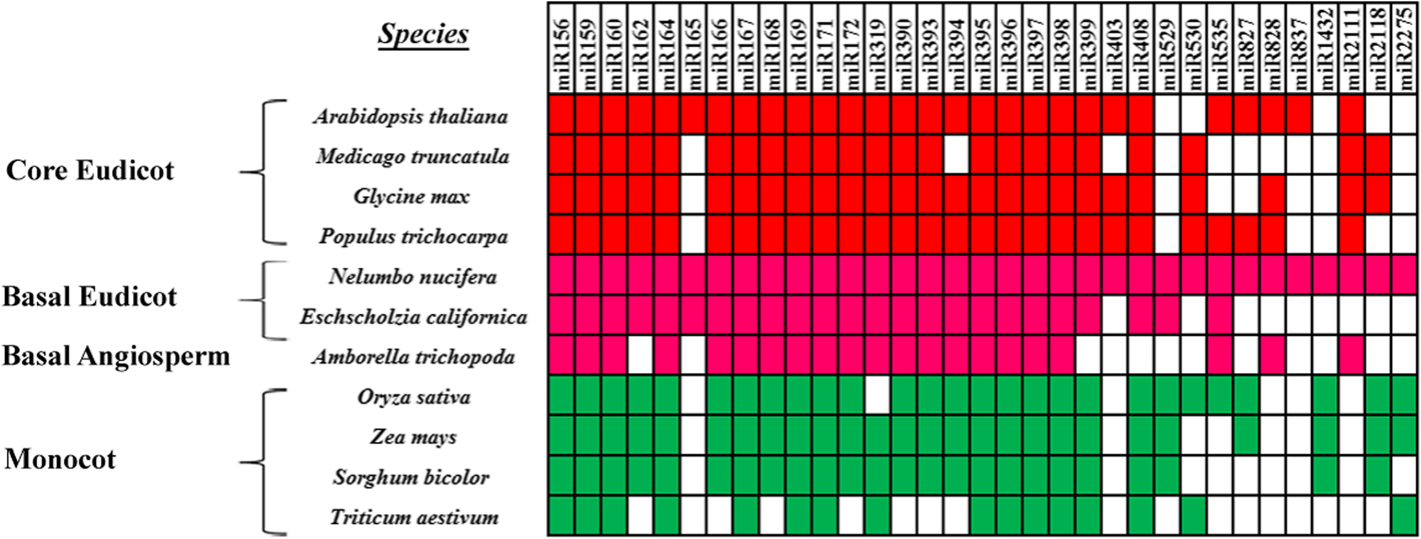
miRNA distribution in different plant species. The miRNA data were obtained from the miRbase 20 and the present study

A comparison of the identified known miRNA families from sacred lotus with other three different plants (Arabidopsis, rice and Amborella) showed that the number of miRNA families widely varied (Table 2) [30, 31]. In sacred lotus, most of the families were represented by from 1 to 8 variants, except for miR396 (15) and miR169 (12). More than 10 variants of miR169 family were also detcted in Arabidopsis and rice (Table 2). When comparing 25 miRNA families of sacred lotus miRNAs to those in 10 other angiosperm species, five families (miR165, miR403, miR828, miR837 and miR2111) were only present in eudicot species. And other two families (miR1432 and miR2275) were found in basal angiosperm and monocots but not detected in core eudicot species (Fig. 1). MiR529 was identified in basal eudicots (*Eschscholzia californica* and *N. nucifera*) and most of the monocots, but no homologues were found in core eudicots (Fig. 1).

**Fig. 2:**
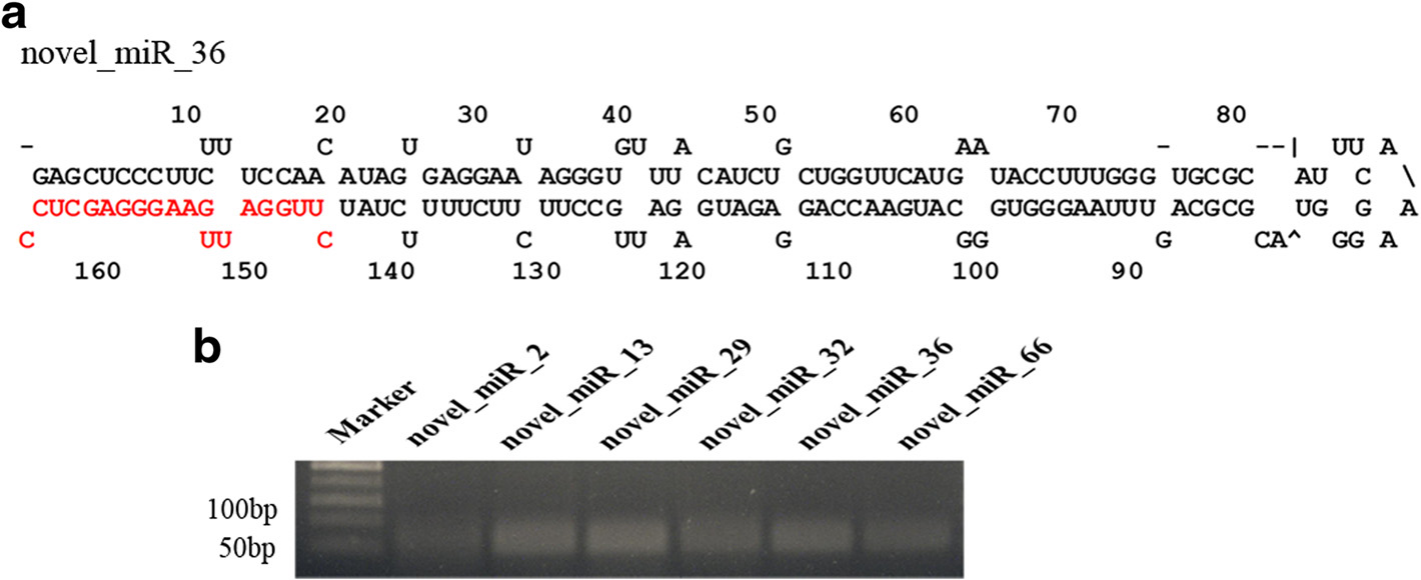
Potential novel miRNAs identified in this study. **a** The secondary structure of novel_ miR_36 precursor was predicted by MFOLD. Mature miRNA is highlighted in red. **b** Stem-loop RT-PCR analysis of the identified novel miRNAs. Six novel miRNAs (novel_miR_2, novel_miR_13, novel_miR_29, novel_miR_32, novel_miR_36, novel_miR_66) were confirmed via stem-loop RT-PCR. The sizes of the obtained PCR products were approximately 60 bp. Marker indicates a 50 bp DNA Ladder Marker

To validate the predicted novel miRNAs, stem-loop RT-PCR was performed for six novel miRNAs (novel_miR_2, novel_miR_13, novel_miR_29, novel_miR_32, novel_miR_36 and novel_miR_66) with relatively high abundance at all the five stages of seed germination (Fig. 2, Additional file 1: Figure S3 and Additional file 2: Table S2). As a result, all the selected six novel miRNAs were found to be expressed in the seed germination (Fig. 2b).

### Differentially expressed miRNA at different stages of seed germination

**Fig. 3:**
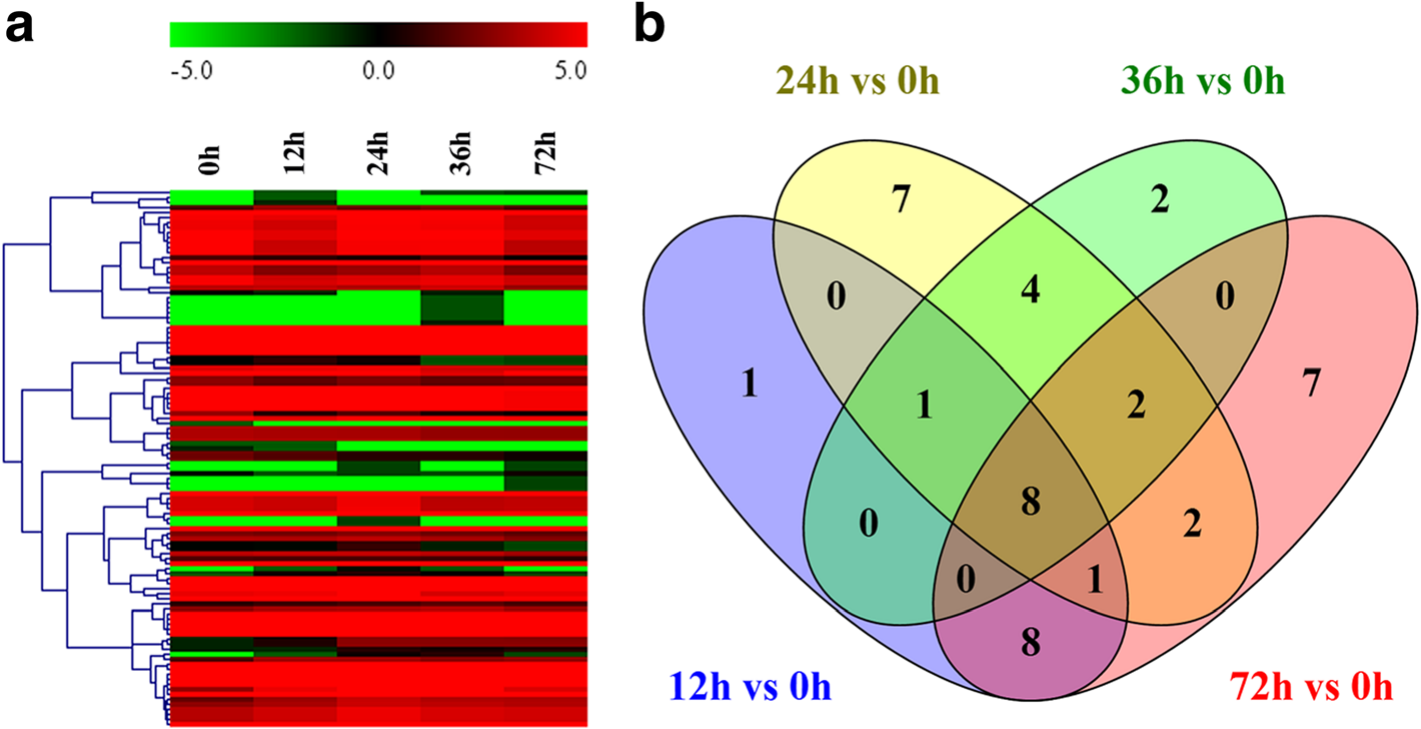
Small RNAs and their expression patterns at each of the five stages during the seed germination of sacred lotus. **a** Heatmap for the clustering analysis of differentially expressed known miRNAs. The bar represents the scale of the expression levels of the miRNAs. **b** Venn diagram of the common and specific known miRNAs at four different stages (12 h, 24 h, 36 h and 72 h) compared with 0 h

To compare the different miRNA expression profiles during the seed germination of sacred lotus, the differential expression analysis of the miRNAs was performed between the four stages with 0 h (CK) of germination, based on the normalized read count for each identified miRNA (Additional file 2: Tables S3 and S4). And a total of 107 differentially expressed (DE) miRNAs were found at the four stages during sacred lotus seed germination. Then cluster analysis was conducted to further elucidate the expression patterns of all known miRNAs, based on the criteria (at least one comparison has a fold change value ≥ 2.0 or ≤ −2.0 with a *p*-value < 0.05) (Fig. 3a). Compared with the 0 h of seed germination, many DE miRNAs were detected in the other four stages (Additional file 2: Table S4). Furthermore, eight DE miRNAs were differentially expressed in all the four RNA libraries, while 26 DE miRNAs were expressed in at least two of the four small RNA libraries, and 17 miRNAs were identified only once in the four samples (Fig. 3b). These results indicated that a larger number of miRNAs were differentially expressed during all five stages of seed germination. Compared with germinating seed at 0 h, a total of 35 DE miRNAs were up-regulated, while 16 miRNAs were down-regulated (Table 3). For instance, Nnu-miR156c-5p and Nnu-miR159a were significantly expressed at 36 h (Table 3). Nnu-miR165a-5p, Nnu-miR166c, Nnu-miR166d and Nnu-miR2111b showed the highest expression levels at 24 h, while Nnu-miR396b-3p showed maximum expression level at 0 h (Table 3). Most of the novel miRNAs showed low expression levels and some of them were only found at one stage during the seed germination. However, novel_mir_13, novel_mir_33 and novel_mir_39 were highly expressed during all the five stages (Additional file 2: Table S4) and stem-loop RT-PCR also confirmed the expression of novel_mir_13 during sacred lotus seed germination (Fig. 2b).

**Table 3:** Differential expressed known miRNAs during the sacred lotus seed germination

Change	miRNA	0 h-std	12 h-std	24 h-std	36 h-std	72 h-std	Significance
Up-regulated	Nnu-miR156c-5p	6.68	10.04	10.59	12.26	8.62	*
	Nnu-miR157a	56.90	100.44	144.83	169.62	69.79	**
	Nnu-miR159a	164.04	434.63	1008.33	1065.40	334.58	**
	Nnu-miR160b	6.99	10.04	16.09	13.28	11.49	**
	Nnu-miR162a-3p	78.52	132.40	218.52	125.68	167.91	**
	Nnu-miR165a-5p	8.27	25.26	45.31	32.02	20.12	**
	Nnu-miR165a-3p	5100.3	10745.91	18344.31	10534.78	10529.28	**
	Nnu-miR166b-5p	0.64	1.52	6.78	6.47	6.57	**
	Nnu-miR166c	5106.39	10766.00	18370.99	10560.33	10551.04	**
	Nnu-miR166d	2117.83	5231.12	9055.48	5330.06	4818.39	**
	Nnu-miR167a-3p	0	0.3	1.27	1.70	0.41	*
	Nnu-miR2111b	2.86	8.83	8.89	6.47	7.39	**
	Nnu-miR319b	103.63	150.05	214.29	149.52	114.13	**
	Nnu-miR319d	736.57	1670.66	2534.59	1737.40	1320.68	**
Down-regulated	Nnu-miR1030	4.45	3.04	1.69	1.70	1.23	*
	Nnu-miR170a	33.06	15.52	29.22	29.63	12.73	**
	Nnu-miR171g	33.38	16.44	30.07	29.97	12.73	**
	Nnu-miR2111a-5p	137.01	98.01	99.10	122.62	55.83	**
	Nnu-miR393a-5p	57.86	39.87	42.77	37.13	28.74	**
	Nnu-miR393c	57.22	38.65	42.35	36.78	28.33	**
	Nnu-miR394a	61.67	19.18	28.37	30.99	22.58	**
	Nnu-miR394b	103.32	44.13	52.09	53.13	32.43	**
	Nnu-miR396b-3p	8.27	1.83	3.39	1.70	1.23	**
	Nnu-miR408a	13.35	6.09	8.05	12.26	5.34	**
	Nnu-miR408c	29.56	17.04	20.75	24.18	16.01	**
	Nnu-miR530a	4.45	3.04	1.69	1.70	1.23	*

"std" represents normalized expression level (Normalised expression, RPM) of miRNAs

"*" and "**" represnent significant differentially expression between 0 h and other hours, *p < 0.05, **p < 0.01

### Degradome sequencing analysis

In this study, a degradome library was constructed from the germinating seed of sacred lotus, followed by degradome sequencing. After discarding the low quality sequences, a total of 13,852,945 clean reads with 4,362,200 unique clean reads was remained. And 99.53 % (13,787,820) of the total sequences, corresponding to representing 99.35 % (4,333,854) of the unique sequences, were mapped to the reference genome to identify the fragments of degraded mRNAs.

**Fig. 4:**
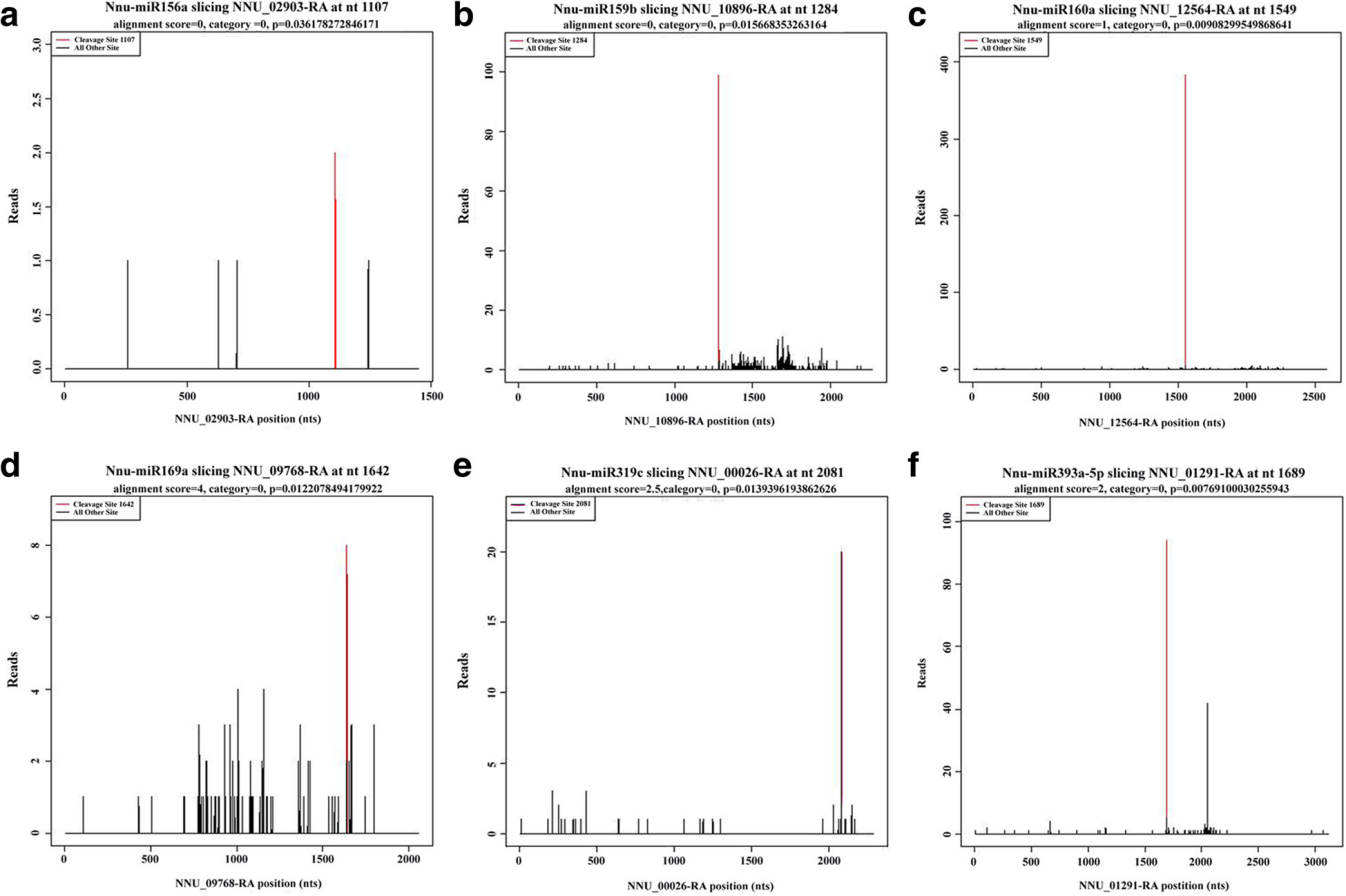
Target plots (t-plots) of six identified known miRNA targets using degradome sequencing. The red lines indicate signatures consistent with miRNA-directed cleavage. **a** Nnu-miR156a slicing NNU_02903-RA at nt 1107(SPL17: Squamosa promoter-binding-like protein 17). **b** Nnu-miR159b slicing NNU_10896-RA at nt 1284 (GAM1: Transcription factor GAMYB). **c** Nnu-miR160a slicing NNU_12564-RA at nt 1549 (ARF18: Auxin response factor 18). **d** Nnu-miR169a slicing NNU_09768-RA at nt 1642 (NFYA10: Nuclear transcription factor Y subunit A-10). **e** Nnu-miR319c slicing NNU_00026-RA at nt 2081(TCP2: Transcription factor TCP2). **f** Nnu-miR393a-5p slicing NNU_01291-RA at nt 1689 (TIR1: Protein TRANSPORT INHIBITOR RESPONSE 1). "alignment score" is the score for mismatch. Score = 0 represents perfect match and G:U = 0.5

Cleavage of target mRNAs of the miRNAs were identified by degradome sequencing (Fig. 4 and Additional file 1: Figure S4). Generally, the target genes of kown miRNAs were mostly transcription factors [32], and similar results were also obtained for the novel miRNAs (Additional file 2: Tables S5 and S6). For example, both Nnu-miR159b and Nnu-miR319c targeted mRNA encoding the MYB transcription factor. Furthermore, Nnu-miR156a, Nnu-miR160a, Nnu-miR160a-5p and Nnu-miR169a targeted mRNA encoding squamosa promoter-binding-like protein 17, auxin response factor 18, NAC transcription factor and nuclear transcription factor Y subunit A-10, respectively (Fig. 4, Additional file 1: Figure S4 and Additional file 2: Table S5). The targets of novel miRNAs were also identified by degradome sequencing and many of them were transcription factors, including GAMYB (novel_mir_13), MYB (novel_mir_32), NAC (novel_mir_36) and TCP (novel_mir_27) (Additional file 2: Table S6).

### Functional annotation of the target genes

**Fig. 5:**
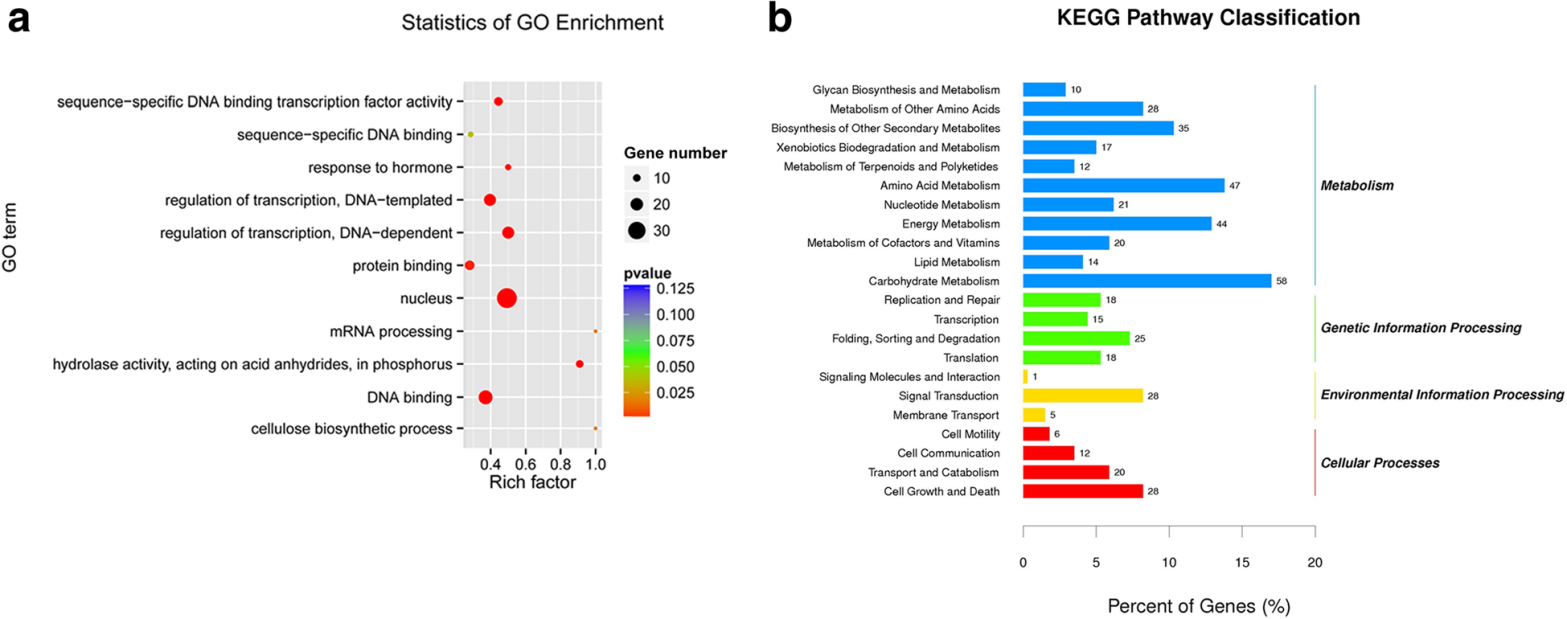
GO analysis and KEGG pathways of the target genes. **a** GO analysis of the target genes for differentially expressed miRNAs. The colouring of the p-values indicates the significance of the rich factor. The circle indicates the target genes that are involved, and the size is proportional to the gene numbers. The Y-axis represents the name of enrichment GO terms. The X-axis represents rich factor. The "Rich factor" was calculated by the number of genes mapped to the GO term divided by the number of all genes in the input list. **b** KEGG pathways of the target genes for differentially expressed miRNAs

To further perceive the putative function of the predicted target genes, Gene Ontology (GO) analysis was performed. And twenty-five different biological processes, fifteen different cellular components and ten different molecular functions were predicted (Additional file 1: Figure S5 and Additional file 2: Table S7). The most significantly enriched GO terms were involved in the three main categories, “protein phosphorylation”, “nucleus”, and “ATP binding”, followed by “regulation of transcription”, “membrane” and “DNA binding” (Fig. 5a and Additional file 1: Figure S5). These results suggested that regulation of transcription played a vital role in the process of the seed germination of sacred lotus. Moreover, all the targets could be used for KOG classifications which were classified into 23 groups (Additional file 1: Figure S6). The most dominative group was the “general function prediction only”, followed by the “transcription” (Additional file 1: Figure S6).

In addition, KEGG analysis was further conducted to elucidate the biological pathways of the DE miRNA target genes, which were classified into five groups including “organismal system”, “metabolism”, “genetic information processing”, “environment information processes” and “cellular processes” (Fig. 5b and Additional file 2: Table S8). The categories of “carbohydrate metabolism”, “amino acid metabolism”, “energy metabolism” and “biosynthesis of other secondary metabolism” were the most enriched pathways, which were all in the group of “metabolism”, suggesting that the miRNA regulating the expression of metabolic genes played crucial roles in the seed germination.

### Quantitative RT-PCR validation of miRNAs and their target genes

**Fig. 6:**
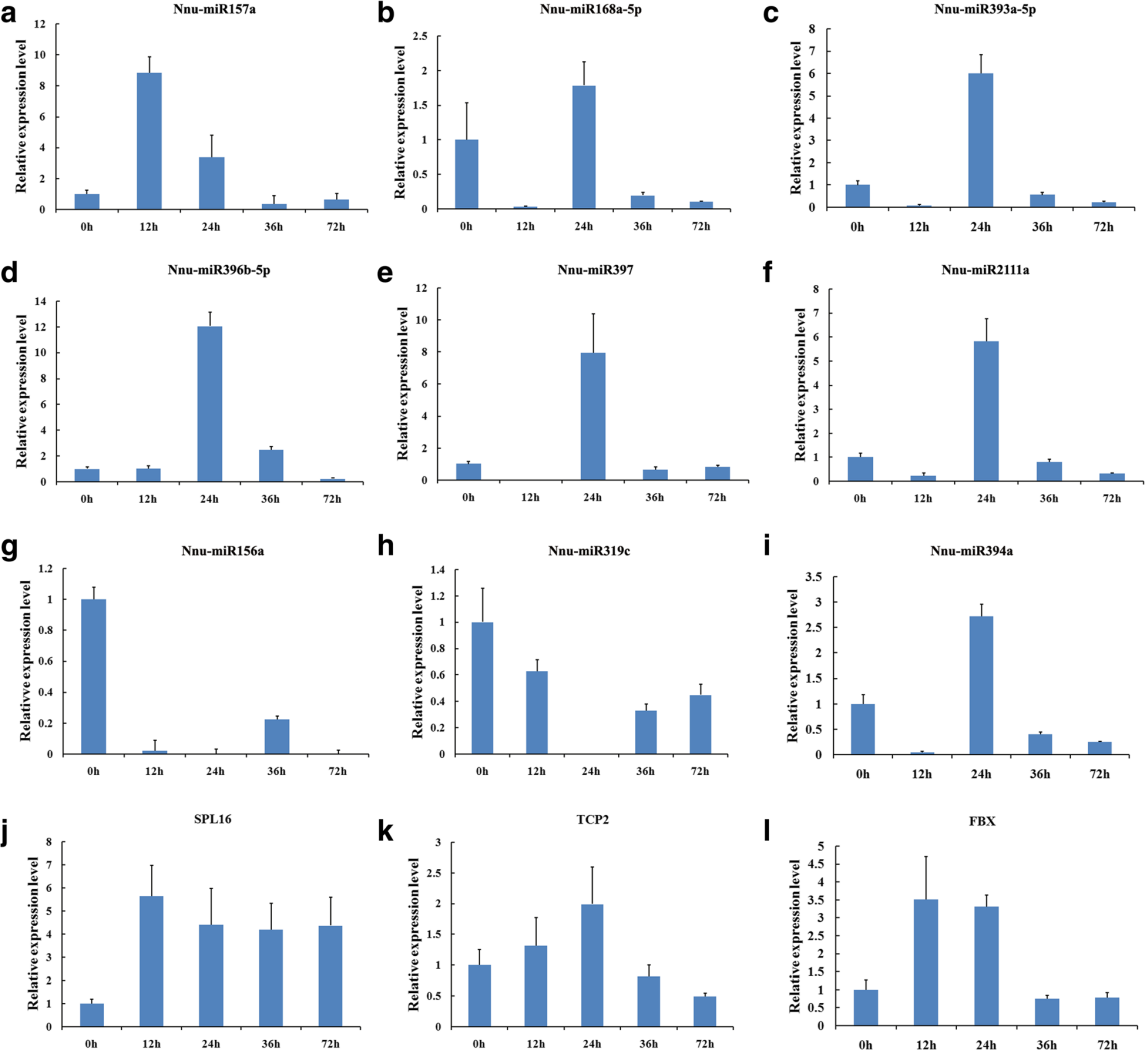
Validation of differentially expressed miRNAs and some of their targets during the seed germination in sacred lotus by qRT-PCR **a** Nnu-miR157a, **b** Nnu-miR168a-5p, **c** Nnu-miR393A-5p, **d** Nnu-miR396b-5p, **e** Nnu-miR397, **f** Nnu-miR2111a,  **g** Nnu-miR156a, **h** Nnu-miR319c, **i** Nnu-miR394a, ** j** Target of Nnu-miR156a (NNU_02036-RA, SPL16), **k** Target of Nnu-miR319c (NNU_00026-RA, TCP2), **l** Target of Nnu-miR394a (NNU_22183-RA, FBX)

In this study, real time quantitative RT-PCR (qRT-PCR) was performed to confirm the expression levels of nine significantly DE miRNAs and three respective predicted target genes, obtained from the high-throughput sequencing (Fig. 6 and Additional file 2: Table S5). Three miRNAs (Nnu-miR393a-5p, Nnu-miR396b-5p and Nnu-miR397) showed the similar expression pattern in terms of read abundance during the five stages of seed germination (Fig. 6 and Additional file 2: Table S3). For instance, Nnu-miR156a showed the highest expression levels at 0 h, and then gradually declined from 12 h to 72 h (Fig. 6g). Moreover, the expression levels of Nnu-miR393a-5p, Nnu-miR394a, Nnu-miR396b-5p, Nnu-miR397 and Nnu-miR2111a were up-regulated and maximized at 24 h and then dramatically decreased to relatively low levels at 36 h and 72 h (Fig. 6). Some of the miRNAs showed the minor discrepancies between sequencing data and the qPCR results, which partially due to the differences in the sensitivity, specificity and algorithm between the two techniques.

The expression levels of some of the predicted target genes (SPL16, TCP2 and FBX) were selected for validation using qRT-PCR, and multiple of target genes showed contrasting expression patterns with their corresponding miRNAs (Fig. 6 and Additional file 2: Table S5). For example, TCP2 (NNU_00026-RA) was the predicted target gene of Nnu-miR319c which expression level was sharply declined to the lowest level at 24 h, then gradually increased and remained at a relatively high level at 72 h. On the contrary, the target (TCP2) of the Nnu-miR319c was increased at the 24 h and then gradually declined at 72 h (Fig. 6h and k). Moreover, Nnu-miR156a also showed a negative relationship with its target SPL16 (Fig. 6g and j). The results suggested that some miRNAs might be invovled in the regulation of seed germination of sacred lotus [33]. However, the expression of FBX showed partially negative relationship with Nnu-miR394a and both of them showed high expression levels at 24 h (Fig. 6i and l). Thus, it is likely that the inverse relationship between miRNAs and their targets might be limited during specific stages of germination, because miRNAs are not the only regulatory factors affecting their targets [34]. Since miRNAs have been shown to also act by translation inhibition in plants, the fact that the RNA expression level of a target is not negatively correlated with miRNA expression level does not mean that the target is not real.

### Putative microRNA editing in sacred lotus

**Fig. 7:**
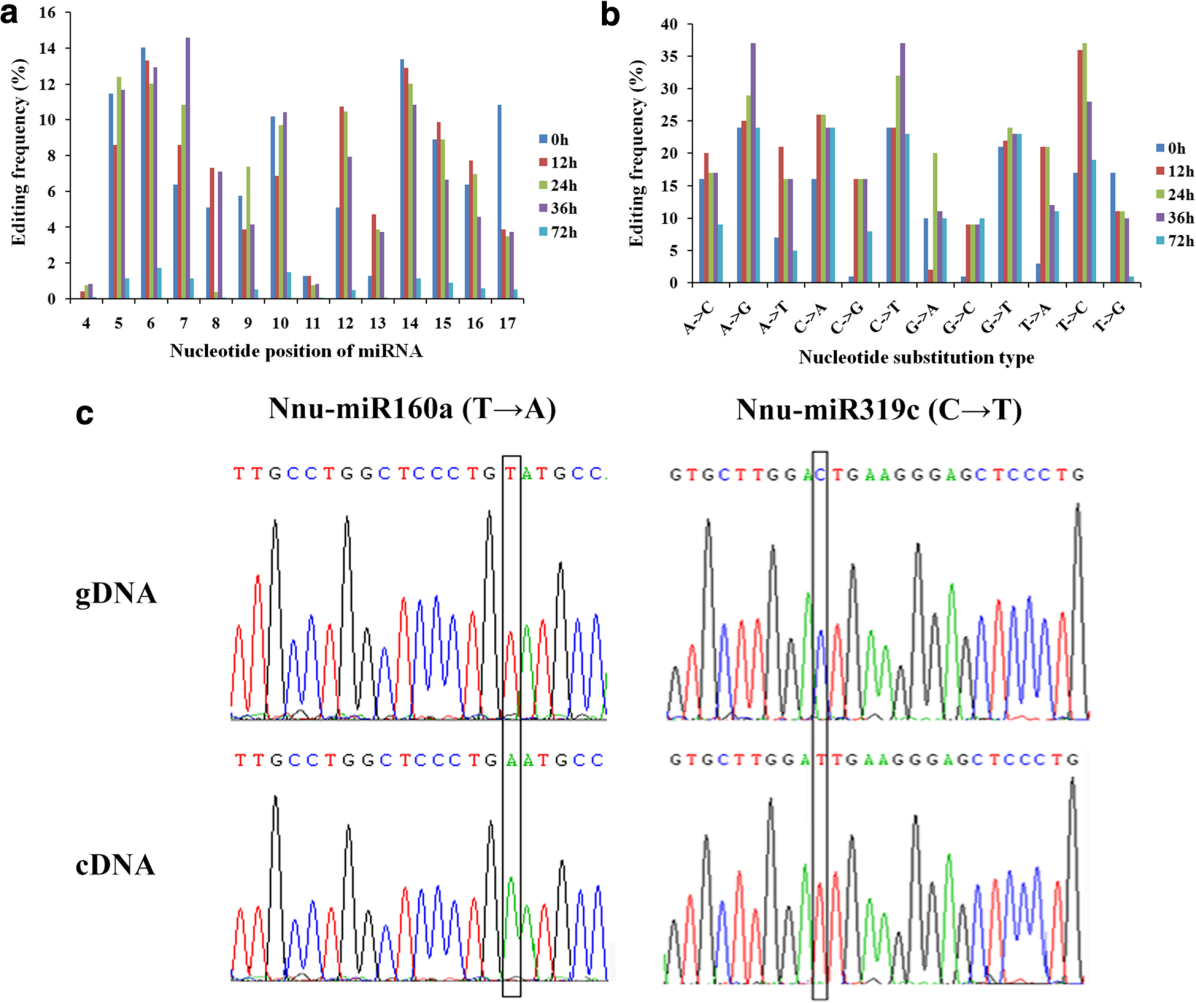
Analysis of miRNA editing events. **a** Summary of the nucleotide substitutions positions among miRNAs. **b** Summary of the nucleotide substitution types among miRNAs. **c** Validation of the editing sites in miRNAs obtained from deep sequencing by Sanger sequencing. The edited sites are highlighted with black frames. The upper is sequences of genomic DNA (gDNA), and the lower is sequences of cDNA

The miRNA editing patterns showed a significant discrepancy among the germinating seeds at 0 h, 12 h, 24 h, 36 h and 72 h (Fig. 7a,b). For example, the most dominant nucleotide substitution type were at 12 h and 24 h with T to C (more than 35 % in both libraries); while the most common nucleotide substitutions were at 0 h and 36 h with A to G and C to T (Fig. 7b). At the nucleotide positions of 4 and 11, the miRNA editing events showed the specifically lowest number (less than 5 number of editing) (Fig. 7a, b and Additional file 2: Table S9). These results were consistent with previous reported miRNA editing in rice grain filling [35].

To validate the occurrence of miRNA editing events in sacred lotus, two editing types (T to A, and U to C) were examined in Nnu-miR160a and Nnu-miR319c, respectively (Fig. 7c). Using PCR amplification, precursor miRNA sequences from genomic DNA and mature miRNA sequences from cDNA were cloned and sequenced, respectively. Comparison analysis of the sequences further confirmed that the miRNA editing events really existed in sacred lotus (Fig. 7c).

## Discussion

As non-coding RNAs, miRNAs play key roles in plant growth and development. Many studies of miRNAs have been reported in model plants such as rice, Arabidopsis and maize and so on. Sacred lotus is a basal eudicot with ornamental, agricultural, medicinal importance. Previous studies on miRNAs in sacred lotus were only in leaves or flowers and for bioinformatics searching without experimentally validation [14, 15]. Phased small interfering RNAs (phasiRNAs) which regulated by miRNAs were also identified in Chinese scared lotus by bioinformatics analysis [36]. However, the number of miRNAs identified in sacred lotus is very few and little is known about the miRNAs involved in the seed germination of *N. nucifera*. In this study, 148 known miRNAs (belonging to 47 families) and 78 novel miRNAs were identified in sacred lotus via deep sequencing and the results were validated by experimental approaches.

Most of the sacred lotus miRNAs identified in the study were present in monocots and at least one of the core eudicot species, indicating their existence in the ancestor of the two large angiosperm clades. Eleven miRNA families were conserved among monocots, basal angiosperm, basal eudicots and core eudicots, suggesting that these miRNAs were involved in important gene regulation pathways in plants (Fig. 1). However, some miRNAs might become lost or evolve within some angiosperm lingeages [30]. MiR165 and miR828 were found in Arbidopsis and two basal eudicot species, but not reported in monocots, while miR529 was present in monocots but not found in the core eudicot species (Fig. 1). That may be because the last common ancestor of monocots and eudicots likely had miR529, with the gene appearing near the speciation of that last common ancestor, but the gene was lot in the ancestor of core eudicots [30]. In addition, most of the target mRNAs of miR165 and miR166 as well as miR156 and miR529 were the same, respectively [33, 34]. The loss of miR165 or miR529 in monocot or core eudicot species indicated the distinct evolution of these species. Moreover, miR1432 and miR2275 were only present in monocots and *N. nucifera*, suggesting that they might be ancient miRNAs in angiosperm. Further investigation of these miRNAs and their targets would give some insights into the functional divergence and evolution in angiosperm.

Using small RNA sequencing, many novel miRNAs with low abundance are discovered from various developmental stages. In this study, 78 novel miRNAs were identified and some of them were validated in the sacred lotus during seed germination (Fig. 2). The expression profiles of these novel potential miRNAs during the different stages might provide valuable information for seed germination and seed longevity in sacred lotus. In addition, a large number of miRNA editing events were found in the seed germination of sacred lotus. Analysis of the nucleotide positions of editing sites showed that the 4 and 11 sites had less editing frequency and positions of 6 and 7 sites had more editing frequency (Fig. 7a). The results were in accordance with rice grains, while in rice young panicles, the two sites had more editing events [5, 35]. These results indicated that miRNA editing was diversity in different tissues and species.

The computational analysis using the cleaveland pipeline revealed a total of 2580 targets for these known and novel miRNAs by degradome sequencing in the present study. Our results showed that most miRNA families had multiple target genes, while several miRNAs from different miRNA families shared some target genes. GO analysis showed that these targets mainly involved in “protein phosphorylation” and “regulation of transcription” (Additional file 1: Figure S5). For KEGG pathway, “carbohydrate metabolism” was enriched in the germinating seeds of sacred lotus (Fig. 5b), indicating that many carbohydrate metabolism related genes were regulated by miRNAs during the seed germination in sacred lotus.

In many plant species, plant hormones including Gibberellins (GAs) and ABA, can promote and inhibit seed germination. And some studies also indicated that cross talk between auxin and GA or ethylene might affect seed germination [29, 37]. In *Arabidopsis thaliana*, overexpression of MIR160 (35S:MIR160) resulted in reduced ABA sensitivity during germination. Transcriptome analysis of germinating ARF10 and the transgenic plants expressing ARF10 with silent mutations (mARF10) revealed that many ABA-responsive genes were overexpressed in germinating mARF10 seeds [38]. Furthermore, miR160 has been shown to regulate the expression of ARF17 which is another auxin response transcription factor [39]. In this study, the Nnu-miR160 also targeted the ARF17 (NNU_16091-RA) and the expression of the three miRNAs of the miR160 families was up-regulated at 24 h during the seed germination (Additional file 2: Tables S3 and S5). These results indicated that overexpression of miR160 might promote seed germinating by decreasing the ABA levels in the seed germination of sacred lotus. Auxin signal pathways are also regulated by miR393 which targets the auxin receptor TIR1 in Arabidopsis [40]. Two TIR1 gene homologues (NNU_01291-RA and NNU_06904-RA) were also detected for the targets of Nnu-miR393 in our degradome dataset (Additional file 1: Figure S4 and Additional file 2: Table S5). The expression level of Nnu-miR393a-5p was also validated by qRT-PCR (Fig. 6c). The down-regulation of some components in auxin signal transduction by miRNAs might be a regulatory step to decrease ABA sensitivity in mature seeds and to switch to the germination mode [29]. MiR159 has been reported to target GAMYB transcription factors which interact with GA-response elements [4]. In Arabidopsis seeds, miR159 regulates the abundance of MYB33 and MYB101 mRNAs during germination in response to ABA. These two MYB transcription factors were positive regulators of ABA responses and overexpressed miR159 would render plants hyposensitive to ABA [41, 42]. In addition, the two target genes of Nnu-miR159 also participate in aleurone vacuolation which was in GA-induced pathways during seed germination. In this study, Nnu-miR159 targeted three GAMYB genes and their expression were highly up-regulated at 36 h during seed germination (Additional file 2: Table S3).

The Nnu-miR156 family members targeted 7 Squamosa Promoter Binding proteins like (SPL) plant specific transcription factors in the present study (Additional file 1: Figure S4 and Additional file 2: Table S5). The expression levels of Nnu-miR156a and its target gene SPL16 (NNU_02036-RA) were also validated (Fig. 6g and j). In plants, SPLs were reported to be involved in diverse developmental processes including leaf development, phase change and flowering in plants [43]. Overexpression of miR156 in Arabidopsis and rice suppressed the expression of SPL genes and reduced apical dominance, delayed flowering time and affected the juvenile to adult transition [43–45]. MiR156 was also reported to be involved in seed germination in soybean and *Lotus japonicas* [46, 47]. However, unlike the maize [43], the expression level of miR156 in sacred lotus was down-regulated after 72 h of the seed germination (Additional file 2: Table S2). That may be a factor for the long living of sacred lotus seed and viable for nearly thousands years.

Nuclear factor Y (NF-Y) was documented to affect the seed development and was targeted by miR169 in many plants. In the degradome dataset in the present study, NFYA2 (NNU_25577-RA), NFYA3 (NNU_11871-RA) and NFYA10 (NNU_09768-RA) were the target genes of miR169 in sacred lotus (Additional file 1: Figure S4 and Additional file 2: Table S5). In Arabidopsis, overexpression of the target of miR169 (NF-YA5) resulted in ABA hypersensitivity during seed germination [48]. Thus, up-regulation of most of the miR169 genes in sacred lotus might reduce the ABA responsiveness during seed germination (Additional file 2: Table S2).

In plants, seed germination is promoted by appropriate environmental conditions including light, temperature, and nutrient availability [49]. Reactive oxygen species (ROS) are largetly generated during the seed germination and reducing the ROS and minimizing cell damage might affect the seed germination. MiR408 which targets L-ascorbate oxidase (NNU_10795-RA) might scavenge the oxidative species produced during stress. In the present study, all the miRNAs of Nnu-miR408 family were significant differentially expressed, indicating their roles in seed germination (Table 3 and Additional file 2: Table S3). PPR proteins that regulate gene expression in mitochondria and chloroplasts were also regulated by some miRNAs, including Nnu-miR168, Nun-miR2673, novel_mir_7 and novel_mir_40. These miRNAs might affect the pathways of photosynthesis and lipid accumulation during the seed germination [50].

**Fig. 8:**
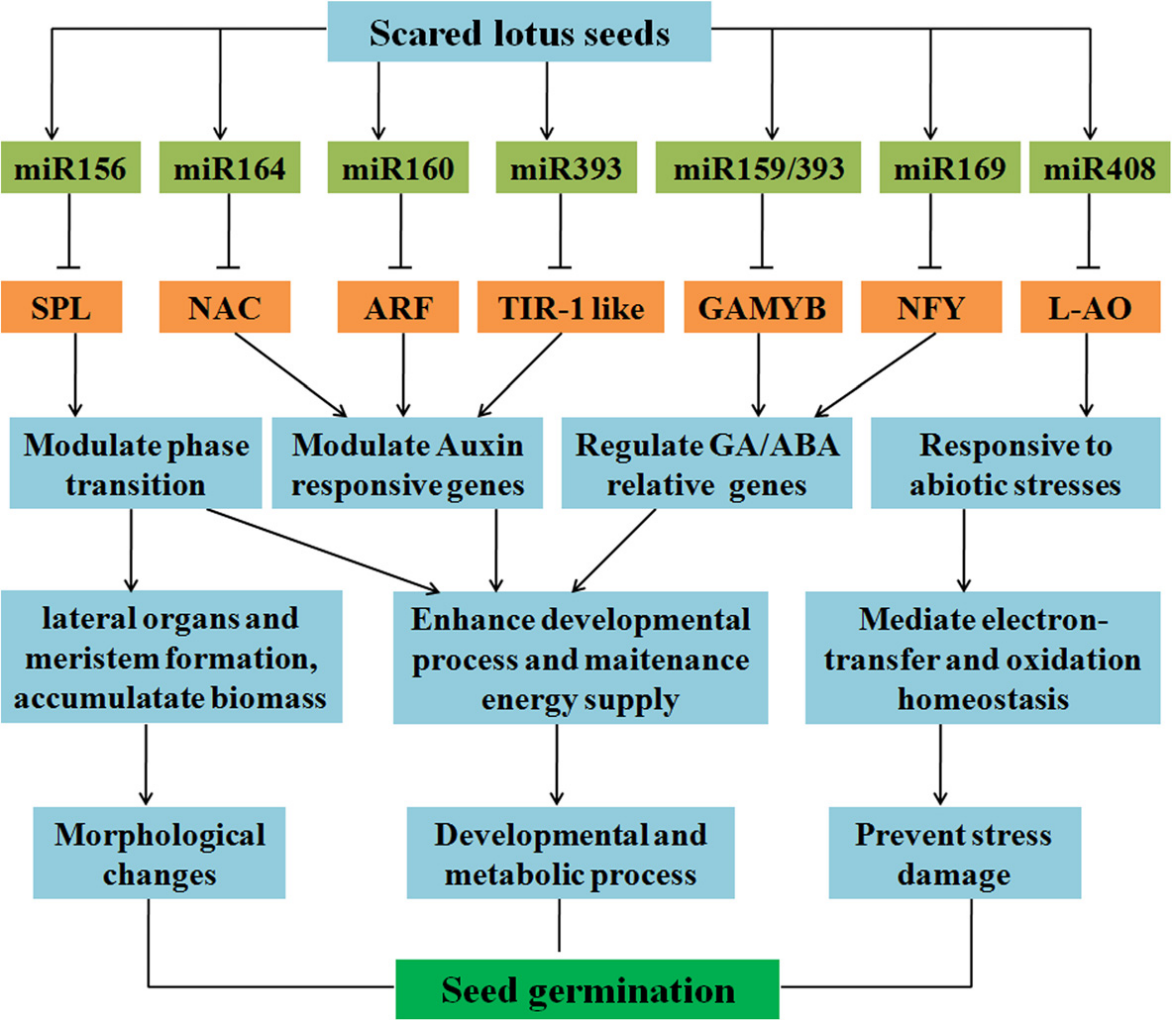
The potential regulatory network for miRNAs in the seed germination of sacred lotus

Taken together, we proposed potential roles for miRNAs playing during the early stages of seed germination in the sacred lotus (Fig. 8). These miRNA mediated the regulation of gene expression in many processes, including morphological changes, developmental process, metabolism and responsive to stress process (Fig. 8). These results indicated that diverse and complex miRNAs were involved in the seed germination. Further functional research and target analyses of the conserved and novel miRNAs could provide additional clues to the different regulation of gene expression during seed germination. The results in this study will lay a foundation for further investigating of long living of sacred lotus.

## Conclusions

In this study, combined with small RNA sequencing and degradome sequencing, 145 known miRNAs and 78 novel miRNAs with 2580 targets were identified during seed germination of sacred lotus. GO and KEGG pathway analyses showed that many target genes enriched in regulation and metabolism. Compared with other angiosperm species, some miRNA families were present or absent in monocots and core eudicots, indicating the functional divergence and evolution in angiosperm. These results suggested that many miRNAs were involved in the regulation of seed germination of sacred lotus and provided insights into the evolutionary gains and losses of miRNAs in plants.

## Methods

### Plant materials

Sacred lotus (*N. nucifera* Gaertn.) seeds used here are maintained in our laboratory. The mature seeds of 3 years old were germinated in distilled water under 16-h light photoperiod at 28 °C in a phytotron. The seeds at 0, 12, 24, 36 and 72 h after imbibition was collected and immediately frozen in liquid nitrogen, then stored in -80 °C until further use.

### RNA isolation and small RNA library construction

Total RNA was extracted from germinating seeds at 0, 12, 24, 36 and 72 h after imbibition using TRIZOL reagent (TianGen, China) according to the manufacturer’s instructions. At least five germinating seeds were mixed for each sample. RNA-free Dnase I (Promega) was then used to remove DNA contamination from total RNA for 30 min at 37 °C. RNA quantity was determined by a Qubit Fluometer. The RNA purity and integrity was detected by Agilent 2100 Bioanalyzer.

Small RNAs of 18 ~ 30 nt were purified from the 5 μg of total RNA by 15 % polyacrylamide gel electrophoresis (PAGE) as described previously [51]. A pair of Solexa adaptors was ligated to the 5′ and 3′ termini of the small RNAs. And then reverse transcription was performed by Super-Script II Reverse Transcriptase (Invitrogen) and amplified using 15 PCR cycles to produce sequencing library. The final quality of the cDNA libraries were examined with the Agilent 2100 Bioanalyzer. The small RNA fragments were finally sequenced by Hiseq2, 500 platforms.

### Bioinformatics analysis of the miRNAs

The raw reads from the Illumina sequencing were first filtered to remove low-quality reads which including reads in length <18 nt, 5′ adaptor contaminates, reads with polyA, and reads without 3′ adaptor. And then adaptor sequences were trimmed to get clean reads. All unique sequences were aligned against the sacred lotus genome (http://lotus-db.wbgcas.cn/) to map the sequences using SOAP [52, 53].

Subsequently, the clean sequences were used to search the RepeatMasker and Rfam database to annotate repeats, rRNA, tRNA, scRNA, snoRNA and snRNA. After removing the sequences belonging to rRNA, tRNA, scRNA, snoRNA, snRNA and repeat sequence tags, the remaining sequences were used to BLAST against miRbase 20.0 (http://www.mirbase.org) to identify conserved miRNA [54]. The remaining unannotated small RNA tags were used to predict novel miRNAs using the miRNA prediction algorithm MIREAP (http://sourceforge.net/projects/mireap). This program was utilized to identify novel miRNA candidates by exploring the secondary structures, Dicer cleavage sites and the minimum free energy (MFE) of the unannotated small RNA tags that could be mapped to the genome. The parameters for identifying potential miRNAs or pre-miRNAs as described previously [5, 33]. And the secondary structures of potential precursor miRNAs were constructed by MFOLD 3.2 [55].

To identify the expression patterns of miRNAs among the five different stages, the read count of each identified miRNA was normalized to reads per million (RPM) using the following formula: normalized expression = count of miRNA/total count of clean reads × 10^-6^. If one miRNA have no reads in a sample, the expression was arbitrarily set to 0.01 for further calculation [56]. Fold change of log2 scale value and *P* value were calculated using the formula as described [57]. The expression of |fold change| >2 and *P* value <0.01 was as significant differential expression. All the significantly differential expression profiles were clustered into different groups by Cluster 3.0 with default parameters.

### Degradome library construction and data analysis

The degradome library was constructed as described previously [13]. Poly (A) RNA was extracted from total RNA of mixture of the five samples (1 μg of total RNA for each) using the Oligotex kit (Qiagen). With 5′-monophosphates, polyadenylated transcripts were ligated to RNA adapters which consisted of a *Mme* I recognition site at its 3′ end. Then, first-strand cDNA was reverse-transcribed using oligo d (T) and amplified for five PCR cycles. The PCR product was purified and digested with *Mme* I and was then ligated to a 3′ double DNA adapter. After amplifying 18 PCR cycles, the PCR product was gel-purified for deep sequencing. The sliced miRNA targets were identified according to the CleaveLand pipeline [13]. The potential targets were as followed: alignments with scores less than four (G: U pairs scored 0.5), and no mismatches were found at the site between the 10th and 11th nucleotides of the miRNAs.

The target genes were annotated using the information from Lotus-DB (http://lotus-db.wbgcas.cn/) [47]. The functional classification and pathways based on the three database: KOG (Clusters of Orthologous Groups of proteins), KO (KEGG Ortholog database) and GO (Gene Ontology), using BLAST with a cutoff E-value of 10^−5^. GO enrichment analysis was implemented by the TopGO R package and GO terms with corrected FDR < 0.05 were considered significantly enrichment [58].

### Identification of potential miRNA editing sites

The miRNA editing sites located on the known miRNA sequences were identified according the method as described previously [35]. The RNA editing level was calculated by the ratio of the reads support the mismatch in the site to the total reads detected on this site. The common and specific miRNAs with RNA editing during the different time points were also analyzed. Genomic DNA and total RNA were isolated from germinating seeds of sacred lotus to validate miRNA editing. Precursor miRNA sequences were amplified from DNA, and the corresponding mature miRNA sequences were also amplified from cDNA which was reverse-transcribed using stem-loop RT-PCR. Then the PCR products were purified, cloned and sequenced with a minimum of six clones for DNA products as well as at least twelve clones for miRNA PCR products. All primers used in this study are listed in Additional file 2: Table S10.

### Detection of potential miRNA using stem-loop RT-PCR and qRT-PCR

Total RNA was extracted from sacred lotus germinating seeds at different stages. Then, RNase-free DNase I (Promega) was used to remove the DNA contamination by incubating at 37 °C for 30 min. For each miRNA, approximately 2 μg of total RNA was reverse-transcribed using miRNA-specific stem-loop primers in a 20 μl of reaction volume using a Fermentas RevertAid First Strand cDNA Synthesis Kit (Fermentas, USA) [59]. The reactions were incubated for 30 min at 16 °C, followed by pulsed RT of 60 cycles at 30 °C for 30s, 42 °C for 30s and 50 °C for 1 s and finally the reactions were terminated at 70 °C for 5 min [59]. The cDNA for the miRNA target was generated using 2 μg of total RNA and OligodT18 primer with the RevertAid First Strand cDNA Synthesis Kit (Fermentas, USA). The novel miRNAs were cloned using specific primers and the PCR products were detected by gel electrophoresis. All the primers were listed in Additional file 2: Table S10.

Real time qRT-PCR analysis of the miRNA and their targets was performed using the FastStart Universal SYBR Green Master Mix (Roche) on the StepOne plus PCR platform (Applied Biosystems). The qRT-PCR reactions were conducted with the following protocol: 95 °C for 10 min, followed by 40 cycles of 95 °C for 15 s and 56 °C for 30s and 72 °C for 15 s. Scared lotus *NnEF1a* (GI: 226897264) was used as an endogenous control. To avoid non-specific amplification, melting curve was carried out for each PCR product. The expression level of the miRNAs and their targets in different samples were calculated by comparative 2^-△△^CT method [60].

### Availability of supporting data

All the sequencing data were deposited in the NCBI Short Read Archive (SRA) database under the accession number SRP070743.

## Additional files

Additional file 1: Figure S1. Size distribution of small RNAs in all of the five libraries from germinating seeds at 0 h, 12 h, 24 h, 36 h and 72 h. **Figure S2.** Venn chart for total sRNA (A) and unique sRNAs (B) between the adjacent pairwise libraries. **Figure S3.** The secondary structures of novel miRNA precursors in sacred lotus. The mature miRNAs are in red and miRNA* in blue. ("." represent baase mismatches, "("represent base matches). **Figure S4.** Target plats (T-plots) of identified known and novel miRNA targets using degradome sequencing. The red lines indicate signatures consistent with miRNA-directed cleavage. **Figure S5.** GO classification of target transcripts for all identified miRNAs in sacred lotus. **Figure S6.** KOG function classification of the miRNA targets in sacred lotus. (PDF 3161 kb) Additional file 2: Table S1. Detailed information of the known miRNAs identified in sacred lotus during seed germination. **Table S2.** Detailed information of the novel miRNAs identified from sacred lotus during seed germination. **Table S3.** The expression level of all known miRNAs of the germinating seeds at 0 h, 12 h, 24 h, 36 h and 72 h. **Table S4.** The significantly differential expression level of the known miRNAs of the germinating seeds at 12 h, 24 h, 36 h and 72 h in comparison with 0 h. **Table S5.** The targets of known miRNA identified in sacred lotus during the seed germination. **Table S6.** The targets of novel miRNA predicted in sacred lotus during the seed germination. **Table S7.** GO enrichment analysis for all the target genes. **Table S8.** KEGG pathway enrichment analysis for the targets of differentially expressed miRNAs. **Table S9.** The common and specific miRNA editing in sacred lotus during the seed germination. **Table S10.** Primers of miRNAs and targets in sacred lotus for qRT-PCR and miRNA editing in this study. (XLSX 203 kb)

## Abbreviations

ABA: Abscisic acid
ARF: Auxin response factor
DE: Differentially expressed
FBX: F-Box
GA: Gibberellins
GO: Gene Ontology
HSP: Heat shock protein
KEGG: Kyoto Encyclopedia of Genes and Genomes
KOG: Eukaryotic Orthologous Groups
MFE: Minimum free energy
miRNA: MicroRNA
NAC: NAM, ATAF, and CUC
NF-Y: Nuclear factor Y
PAGE: Polyacrylamide gel electrophoresis
PCD: Programmed cell death
PPR: Pentatricopeptide repeat
qRT-PCR: Quantitative reverse transcription polymerase chain reaction
ROS: Reactive oxygen species
RPM: Reads per million
SOAP: Short oligonucleotide alignment program
SPL: SQUAMOSA promoter-binding protein
TCP: TEOSINTE BRANCHED/CYCLOIDEA/PCF

## References

[1] Bartel DP (2004). MicroRNAs: Genomics, Biogenesis, Mechanism, and Function. Cell.

[2] Voinnet O (2009). Origin, biogenesis, and activity of plant microRNAs. Cell.

[3] Chen XM (2004). A microRNA as a translational repressor of APETALA2 in Arabidopsis flower development. Science.

[4] Millar AA, Gubler F (2005). The Arabidopsis GAMYB-like genes, MYB33 and MYB65, are microRNA-regulated genes that redundantly facilitate anther development. Plant Cell.

[5] Yan J, Zhang HY, Zheng YZ, Ding Y (2015). Comparative expression profiling of miRNAs between the cytoplasmic male sterile line Meixiang A and its maintainer line Meixiang B during rice anther development. Planta.

[6] Chuck G, Cigan AM, Saeteurn K, Hake S (2007). The heterochronic maize mutant Corngrass1 results from overexpression of a tandem microRNA. Nat Genet.

[7] Vaucheret H, Vazquez F, Crete P, Bartel DP (2004). The action of ARGONAUTE1 in the miRNA pathway and its regulation by the miRNA pathway are crucial for plant development. Gene Dev.

[8] Hu JH, Sun LL, Zhu ZX, Zheng Y, Xiong W, Ding Y (2014). Characterization of conserved microRNAs from five different cucurbit species using computational and experimental analysis. Biochimie.

[9] Gutierrez L, Mongelard G, Flokova K, Pacurar DI, Novak O, Staswick P (2012). Auxin controls Arabidopsis adventitious root initiation by regulating jasmonic acid homeostasis. Plant Cell.

[10] Vidal EA, Araus V, Lu C, Parry G, Green PJ, Coruzzi GM (2010). Nitrate-responsive miR393/AFB3 regulatory module controls root system architecture in Arabidopsis thaliana. Proc Natl Acad Sci U S A.

[11] German MA, Pillay M, Jeong DH, Hetawal A, Luo SJ, Janardhanan P (2008). Global identification of microRNA-target RNA pairs by parallel analysis of RNA ends. Nat Biotechnol.

[12] Zhou M, Gu LF, Li PC, Song XW, Wei LY (2010). Degradome sequencing reveals endogenous small RNA targets in rice (*Oryza sativa* L. ssp. indica). Front Biol.

[13] Liu N, Tu LL, Tang WX, Gao WH, Lindsey K, Zhang XL (2014). Small RNA and degradome profiling reveals a role for miRNAs and their targets in the developing fibers of Gossypium barbadense. Plant J.

[14] Zheng Y, Jagadeeswaran G, Gowdu K, Wang N, Li SH, Ming R (2013). Genome-wide analysis of microRNAs in sacred lotus, *Nelumbo nucifera* (Gaertn). Trop Plant Biol.

[15] Pan L, Wang XL, Jin J, Yu XL, Hu JH (2015). Bioinformatic identification and expression analysis of *Nelumbo nucifera* microRNA and their targets. Appl Plant Sci.

[16] Wu ZH, Gui ST, Quan ZW, Pan L, Wang SZ, Ke WD (2014). A precise chloroplast genome of *Nelumbo nucifera* (Nelumbonaceae) evaluated with Sanger, Illumina MiSeq, and PacBio RS II sequencing platforms: insight into the plastid evolution of basal eudicots. BMC Plant Biol.

[17] Pan L, Quan ZW, Hu JH, Wang GY, Liu SN, He Y (2011). Genetic diversity and differentiation of lotus (*Nelumbo nucifera*) accessions assessed by simple sequence repeats. Ann Appl Biol.

[18] Yang M, Xu L, Liu YL, Yang PF (2015). RNA-Seq Uncovers SNPs and Alternative Splicing Events in Asian Lotus (*Nelumbo nucifera*). PLoS One.

[19] Wang Y, Fan GY, Liu YM, Sun FM, Shi CC, Liu X (2013). The sacred lotus genome provides insights into the evolution of flowering plants. Plant J.

[20] Ming R, VanBuren R, Liu YL, Yang M, Han YP, Li LT (2013). Genome of the long-living sacred lotus (*Nelumbo nucifera* Gaertn.). Genome Biol.

[21] Shen-Miller J, Turek J, William SJ, Tholandi M, Yang M (2013). Centuries-Old Viable Fruit of Sacred Lotus *Nelumbo nucifera* Gaertn var. China Antique. Trop Plant Biol.

[22] Shen-Miller J, Berger R (1995). Exceptional seed longevity and robust growth: ancient Sacred Lotus from China. Am J Bot.

[23] Kaur H, Petla BP, Kamble NU, Singh A, Rao V, Salvi P, Ghosh S, Majee M (2015). Differentially expressed seed aging responsive heat shock protein OsHSP18.2 implicates in seed vigor, longevity and improves germination and seedling establishment under biotic stress. Front Plant Sci.

[24] Shen-Miller J, Schopf JW, Harbottle G, Cao RJ, Ouyang S, Zhou KS (2002). Long-living lotus: germination and soil {gamma}-irradiation of centuries-old fruits, and cultivation, growth, and phenotypic abnormalities of offspring. Am J Bot.

[25] Chu P, Chen HH, Zhou YL, Li Y, Ding Y, Jiang LW (2012). Proteomic and functional analyses of *Nelumbo nucifera* annexins involved in seed thermotolerance and germination vigor. Planta.

[26] Han C, Yang PF (2015). Studies on the molecular mechanisms of seed germination. Proteomics.

[27] He DL, Wang Q, Wang K, Yang PF (2015). Genome-Wide Dissection of the MicroRNA Expression Profile in Rice Embryo during Early Stages of Seed Germination. PLoS One.

[28] Song QX, Liu YF, Hu XY, Zhang WK, Ma B, Chen SY (2011). Identification of miRNAs and their target genes in developing soybean seeds by deep sequencing. BMC Plant Biol.

[29] Wang L, Liu H, Li D, Chen H (2011). Identification and characterization of maize microRNAs involved in the very early stage of seed germination. BMC Genomics.

[30] Barakat A, Wall K, Leebens‐Mack J, Wang JYJ, Carlson JE, dePamphilis CW (2007). Large‐scale identification of microRNAs from a basal eudicot (*Eschscholzia californica*) and conservation in flowering plants. Plant J.

[31] Albert VA, Barbazuk WB, Der JP, Leebensmack J, Ma H, Palmer JD (2013). The Amborella genome and the evolution of flowering plants. Science.

[32] Jones-Rhoades MW, Bartel DP, Bartel B (2006). MicroRNAs and their regulatory roles in plants. Annu Rev Plant Biol.

[33] Bi F, Meng X, Ma C, Yi G (2015). Identification of miRNAs involved in fruit ripening in Cavendish bananas by deep sequencing. BMC Genomics.

[34] Li T, Ma L, Geng YK, Hao CY, Chen XH, Zhang XY (2015). Small RNA and degradome sequencing reveal complex roles of miRNAs and their targets in developing wheat grains. PLoS One.

[35] Yi R, Zhu ZX, Hu JH, Qian Q, Dai JC, Ding Y (2013). Identification and expression analysis of microRNAs at the grain filling stage in rice (*Oryza sativa* L.) via deep sequencing. PLoS One.

[36] Zheng Y, Wang SP, Sunkar R (2014). Genome-wide discovery and analysis of phased small interfering RNAs in Chinese sacred lotus. PLoS One.

[37] Das SS, Karmakar P, Nandi AK, Sanan-Mishra N (2015). Small RNA mediated regulation of seed germination. Front Plant Sci.

[38] Liu PP, Montgomery TA, Fahlgren N, Kasschau KD (2007). Repression of AUXIN RESPONSE FACTOR10 by microRNA160 is critical for seed germination and post-germination stages. Plant J.

[39] Mallory AC, Bartel DP, Bartel B (2005). MicroRNA-directed regulation of Arabidopsis AUXIN RESPONSE FACTOR17 is essential for proper development and modulates expression of early auxin response genes. Plant Cell.

[40] Navarro L, Dunoyer P, Jay F, Arnold B, Dharmasiri N, Estelle M (2006). A plant miRNA contributes to antibacterial resistance by repressing auxin signaling. Science.

[41] Reyes JL, Chua NH (2007). ABA induction of miR159 controls transcript levels of two MYB factors during Arabidopsis seed germination. Plant J.

[42] Alonso-Peral MM, Li JY, Li YJ, Allen RS, Schnippenkoetter W, Ohms S (2010). The microRNA159-regulated GAMYB-like genes inhibit growth and promote programmed cell death in Arabidopsis. Plant Physiol.

[43] Li DT, Wang LW, Liu X, Cui DZ, Chen TT, Zhang H (2013). Deep sequencing of maize small RNAs reveals a diverse set of microRNA in dry and imbibed seeds. PLoS One.

[44] Xie KB, Wu CQ, Xiong L (2006). Genomic organization, differential expression, and interaction of SQUAMOSA promoter-binding-like transcription factors and microRNA156 in rice. Plant Physiol.

[45] Wang JW, Czech B, Weigel D (2009). miR156-regulated SPL transcription factors define an endogenous flowering pathway in *Arabidopsis thaliana*. Cell.

[46] Shamimuzzaman M, Vodkin L (2012). Identification of soybean seed developmental stage-specific and tissue-specific miRNA targets by degradome sequencing. BMC Genomics.

[47] Hu JH, Zhang HY, Ding Y (2013). Identification of conserved microRNAs and their targets in the model legume *Lotus japonicus*. J Biotech.

[48] Mu J, Tan H, Hong S, Liang Y, Zuo J (2013). Arabidopsis transcription factor genes NF-YA1, 5, 6, and 9 play redundant roles in male gametogenesis, embryogenesis, and seed development. Mol Plant.

[49] Penfield S, Josse E, Kannangara R, Gilday AD, Halliday KJ, Graham IA (2005). Cold and light control seed germination through the bHLH transcription factor SPATULA. Curr Biol.

[50] Wang K, Deng J, Damaris RN, Yang M, Xu LM, Yang PF. LOTUS-DB: an integrative and interactive database for *Nelumbo nucifera* study. Database (Oxford). 2015;2015,bav023. 10.1093/database/bav023 PMC4383347 25819075

[51] Hafner M, Landgraf P, Ludwig J, Rice A, Ojo T, Lin C, Holoch D, Lim C, Tushcl T (2008). Identification of microRNAs and other small regulatory RNAs using cDNA library sequecing. Methods.

[52] Li R, Li Y, Kristiansen K, Wang J (2008). SOAP: short oligonucleotide alignment program. Bioinformatics.

[53] Kozomara A, Griffiths-Jones S (2011). miRBase: integrating microRNA annotation and deep-sequencing data. Nucleic Acids Res.

[54] Zuker M (2003). Mfold web server for nucleic acid folding and hybridization prediction. Nucleic Acids Res.

[55] Chen L, Wang T, Zhao M, Tian Q, Zhang WH (2012). Identification of aluminum-responsive microRNAs in *Medicago truncatula* by genome-wide high-throughput sequencing. Planta.

[56] Audic S, Claverie JM (1997). The significance of digital gene expression profiles. Genome Res.

[57] Addo-Quaye C, Eshoo TW, Bartel DP, Axtell MJ (2008). Endogenous siRNA and miRNA targets identified by sequencing of the Arabidopsis degradome. Curr Biol.

[58] Alexa A, Rahnenfuhrer J, Lengauer T (2006). Improved scoring of functional groups from gene expression data by decorrelating GO graph structure. Bioinformatics.

[59] Varkonyi-Gasic E, Wu R, Wood M, Walton EF, Hellens RP (2007). Protocol: a highly sensitive RT-PCR method for detection and quantification of microRNAs. Plant Methods.

[60] Livak KJ, Schmittgen TD (2001). Analysis of relative gene expression data using real-time quantitative PCR and the 2(-Delta Delta C (T)) Method. Methods.

