# Supplementary material for: Small RNA and degradome profiling reveals miRNA regulation in the seed germination of ancient eudicot Nelumbo nucifera
Source: BMC Genomics. 2016 Aug 26;17(1):684. doi: 10.1186/s12864-016-3032-4 (PMC5002175; doi:10.1186/s12864-016-3032-4)
Supplement: Additional file 1: Figure S1. — Size distribution of small RNAs in all of the five libraries from germinating seeds at 0 h, 12 h, 24 h, 36 h and 72 h. Figure S2. Venn chart for total sRNA (A) and unique sRNAs (B) between the adjacent pairwise libraries. Figure S3. The secondary structures of novel miRNA precursors in sacred lotus. The mature miRNAs are in red and miRNA* in blue. ("." represent baase mismatches, "("represent base matches). Figure S4. Target plats (T-plots) of identified known and novel miRNA targets using degradome sequencing. The red lines indicate signatures consistent with miRNA-directed cleavage. Figure S5. GO classification of target transcripts for all identified miRNAs in sacred lotus. Figure S6. KOG function classification of the miRNA targets in sacred lotus. (PDF 3161 kb) [file 12864_2016_3032_MOESM1_ESM.pdf]

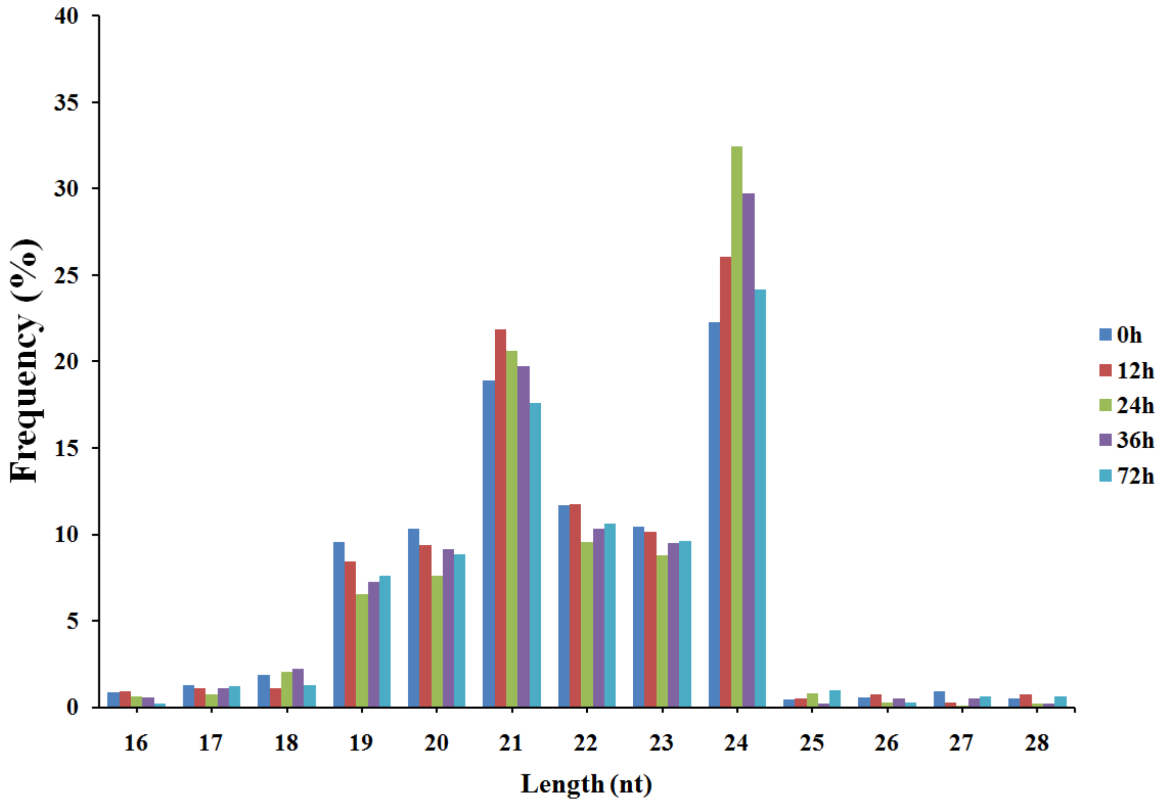

Additional file 1: Figure S1 Size distribution of small RNAs in all five libraries from germinating seed at 0h, 12h, 24h, 36h and 72h.

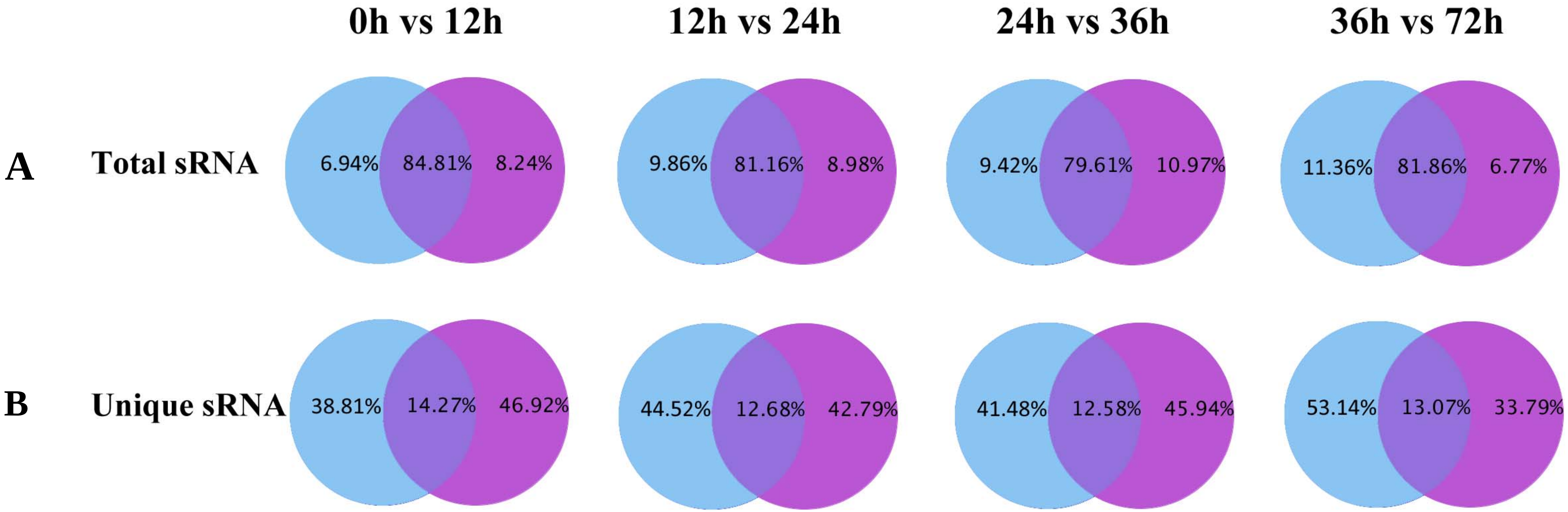

Additional file 1: Figure S2 **Venn chart for total sRNA (A) and unique sRNAs (B) among adjoining stages.**

Additional file 1: Figure S3 The secondary structures of sacred lotus novel miRNA precursors. The mature miRNAs are in red and miRNA* in blue. ("." represent baase mismatches, "(" represent base matches).

novel_mir_1

TCGATGATGGTCTTACGGGTGTCTTCACCTCCGGAGTTGAAGAACATTATCGGAGATTCGTCGTATTTCCATCGGAGGTGAAGGCCACCCGCTAGATCTTCATCGAT

(((((((.(((((..(((((((((((((((((((.(((....)))......((((((.......)))))).))))))))))))).))))))..))))).))))))).

**********TCTTACGGGTGTCTTCACCTC**

novel_mir_10

ATCGGGAGGCTGCGGAATGGACGGGTCGGAAACAAAATTCTCGTCTGTGGGATTGTTTTTTGTTTGCTTCCAACCCCGCCCATTCCATAGTTTTCCGA

.(((((((((((.(((((((.((((..((((((((((..(..((((...)))).)..))))))....))))...)))).))))))).)))))))))))

*************GGAATGGACGGGTCGGAAACA**

CTTCCAACCCCGCCCATTCCAT********

novel_mir_102

ATAAAGGTAATGATCGGGGATGCACTTTCACTGATGGAAACCAAACTACCGGCATTTGATCTCAATCATTGGAATTTTTTTTTGTAAAATCTATATATATGATAT

ATACTTACATATGGTTTCAAAACATTTGCAAATGGGCTAAGAGGTTAAGTGTAAGGCCATTTAGTCAGGCAGTGCTTGATCATTATCCTAAA

.....(((((((((((((.((((.((...((((((((...........((.((((((((((((.....((((((((......(((((....(((((((...))))

))).)))))...))))))))..((((....))))......))))))))))))..))))).))))).))))).).))))))))))))).....

**********TGATCGGGGATGCACTTTCACTG**

****

novel_mir_103

CATTCCCCTCGAGGCAAGCCCGATCAAAGTCTATGTTAGGGTCTGAAATTCTTATTTCTCACATAAAAAATAATAATTTTTTATCAAAAAAATAAAAAATAATGA

TTTTATAACTTTCATTGCCTTTTAAGAAGATATTTTATTGCAATGAATGAATGAACAACATTAGCAAATAAGGGGTTCTCACATAAAAGGCAATGAAAGTTATAA

AATCATTATTTTTTATGTGAGACATAAGAATTTCAGACCCTAACATAGACTTGTTTGGGTTTGCCTCGAGGAGAGT

.((((.(((((((((((((((((.(((.(((((((((((((((((((((((((((.(((((((((((((((....)))))))))).......((((((((((((((

((((((((((((((((((((((((.((.(((.(((((((((((((..((......)).)))).)).))))))).))).))...))))))))))))))))))))))
))))))))))))))))))))))).)))))))))))))))))))))))))))))).))))))))))))))))).))))
****
****
TAGACTTGTTTGGGTTTGCCT*******

novel_mir_104

TCTGGATCTCTTGCCCATGAGAACTCTGCCCACATCTACCCACAACACATTACCTCTGTGAATTGGGAACATCTCCCCTACATAATACTATGTAGGGGGATGAGA
TATTCTCCATGTGTAACTGCAATGTAATGTGGCTAGACGTGGGCAGAGTTTGTATAGACAGGAGATCCAGAC
(((((((((((((.(.(((.(((((((((((((.((((.(((((..(((((...........((((((((((.((((((((((....))))))))))))))...
..))).)))...........)))))..))))).)))).))))))))))))).))).).))))))))))))).
**********TTGCCCATGAGAACTCTGCCC**
****

novel_mir_105

TGCCTTTAGGTAGTAGAATGCCTGTGTCATACTTCCATGTTTTAAAACATTGCAAGGAATAGCTTTTAGACCTTAGTAACATACCAATGTTTTAAAACGCGAAGG
CGTGACACGAGGTGTTCCACTACGTAGAGGCG
.(((((((.(((((.(((((((((((((((.((((..(((((((((((((((.((((..((.....))..))))..........))))))))))))))).)))).
.))))))).))))))))).))))).)))))).
**********TAGTAGAATGCCTGTGTCATACT**
****

novel_mir_106

AAACGGCTTGAGAGAGCTTTCTTCAGTCCACTCATGGATGGGCTAAGGTGTTGAATTAGCTGCCGACTCATTCATTCAAACACTCAGTAGAAAGGGGATATTTTT
TACTACTGTGAATGTGTGAATGATGCGGGAGATAATTGCCATTCTTCCTCCTCTATGCTTGGACTGAAGGGAGCTCCCTTTTTCTGTT
.(((((...(((.(((((((((((((((((..((((((.(((..((((.....(((((.((.(((..((((((((.((..(((..(((((((((......)))))
))))...)))..)).)))))))))..))).)).))))).....))))..))))))))..))))))))))))))))).)))...)))))
****
GCTTGGACTGAAGGGAGCTCCC********

novel_mir_107

TGCGTTGTCTAGGCATTGAGAGTTATGGTATCTCAGTCACAACTTTGGAGGAGGTCTTCCTGAGAGTTGCTGGATGTGACATTGACAAAATTGAGCATATTGAGC
AGAATACGGGTACAAGTGCTGAACAGGTGTGTTTTTTGTTTCAGTTTCTCTCAGCTTTCTTTGGCCAATGGTGCGTAGGGCTCATGCCTTGATTATATA
......(((.((((((.(((..(((((.((((...((((........(((((((.....(((((((..((((((..........(((((...(((((((((...(
((..(((........))))))....))))))))))))))))))))..))))))))))))))))))....)))))))))..))))))))).))).......
****
AATGGTGCGTAGGGCTCATGCCT********

novel_mir_108

GAACGTCCCCCTTCGAGTTCCGCCAGTCAACGCAGATATCCTCCGGCGGCGTCGACTGGAACGATCGTCGGCGTCGACTGGCGGAACTCGAAGGGGGCAAGCT
......((((((((((((((((((((((.((((.((((((.(((((((....)).)))))..))).))).)))).))))))))))))))))))))))......
TCGACTGGCGGAACTCGAAG*******

novel_mir_11

AATGATTAATTGAGATTGATAGAATGTAGGCATAAGTGGTTGTGGATAACCACTTTTGCATTTTCTGCATTTTTTAGGAATTGATTGAAAATG
.....((((((((..((((.((((((((((((.(((((((((....))))))))).))).....)))))))))))))...)))))))).....
TTCTGCATTTTTTAGGAATTG********

novel_mir_110

AGATGCTCCAGACAGAGATGGAGGGATGGATCTAGTTTGCGAGCACAATGGGAGAAAGTGGGGATTCTGGAGCCTTCTCGCACACTAGATCCATCCCTCCATCTC
TGTCTAGATTC
.((..((..(((((((((((((((((((((((((((.((((((.........((((........))))........)))))).))))))))))))))))))))))
)))))))..))
***************AGATGGAGGGATGGATCTAGT***
****
***TAGATCCATCCCTCCATCTC
T**********

novel_mir_115

ACATCAGACTTGAGTTGGATTCTTCTCTGGTTTAACAATCTTTAAATCATTATTGAAGCGTAGTTTTGACTTTTCTGATTTAGTGATTTTTAAATCGGAGTTGAA

TCTTTGTGATGA
.(((((..........((((((..(((((((((((.((((.((((((((.....((((((......)).))))..)))))))).)))).)))))))))))..)))
)))...))))).
**TTTTTAAATCGGAGTTGAA
TC**********

novel_mir_118

GGAGTAGGGAAAGGCAGACAGAGCCGCCGGAACTTATTGGACTGGGTGCCTTGGGAAGTTCTATTACAACTGAGAGTTTAGAGACAGATCTCTCTCTCTCTCT
CGTATAAGTGCTGAACCTTCCCAAGGCCTCCCATGCCGATGATTTCCTGTGGTTCCTCCCTTTCCTTTTC Sample_N36-m0081 9
.(((.(((((((((.((...(((((((.((((.(((((((..((((.((((((((((((((.........((((((...(((((.(((....))).)))))))))
))..........))).)))))))))))..))))..))))))).)))).))))))))).))))))))))))
**************CAGACAGAGCCGCCGGAACTTATT***
****
****
***************************************TGATTTCCTGTGGTTCCTCCC**********

novel_mir_119

TGTTTCATGGTTTTCGAACTGCCATTGACCTAAGCGGTTGGATCGTTTGGCTTGGGAACCGTCCAGACAA
(((((.(((((((((((...((((.(((.((((....)))).)))..)))))))))))))))..))))).
**********TTTTCGAACTGCCATTGACCT***************************************

novel_mir_120

TTCTTTCTTCTTTGCTTCTCTTAAACAGTCTTCTCCCATGAAAAGCTGAATATGTTCTGTTGATGGCAGTGATGTCCCGCCGGCGATGAACGGCTTCATCTTCCA
GCCAGTAGGTTGCTGGTGGATCAGTTGCAGATGAAGCCGTTCGTCGCCGGCGGAATATCACTGCCATCAATAGAACGCATTAAGCTTTTCATGGGAGAAGACTGT
TTAAGAGAAGCAAAGAAGAAGGAAG
(((.(((.(((((((((((((((((((((((.(((((((((((((((((((((((((((((
.((((((...))))))))))........))))))))))))))))))))))))).))))))))))))))))))))))).))).)))))))))))))))))))))))
)))))))))))))))))))))))).
**********TTTGCTTCTCTTAAACAGTCT**

```
*********************************************************************************************************
*************************
```

novel_mir_121

```
ACTAGGTGTCGGATTGGTTTGTCTTTAATGTGTCAATCAGTGAGATCCTTAGAGGGGATCAAAACCTAGGTATGTTGGGACAGTACGAGAATTCATCTAAG
..((((((.....(((..(((((((.((((((((.....((..(((((((...)))))))...))...)))))))))))))))..))).....))))))..
**********************************************************************TATGTTGGGACAGTACGAGAA**********
```

novel_mir_123

```
TTTCATGATCTGAATTTACAGATCTGAGATTGCAAAATAGCAATTCAGACCTTGTAAATTCATGATCTGAAATTTCAGATCTGAGAT
.((((.(((((((((((((((.((((((.((((......)))))))))).).)))))))))).))))))))((((((....))))))
**********TGAATTTACAGATCTGAGATT********************************************************
*******************************************TTCAGACCTTGTAAATTCATG***********************
```

novel_mir_125

```
AAAAGTCTTCTGCATCGGTGACGGACACAAAACTTATTCATATTTATCCTCCATGATCCGGTAGCTGTCTCCTGTCTTATGGGCTTGGCTATGAAGTTCCATTTC
CATAGTTTCTGGCTGGATAGACAAGTCTTGTGTCCAACACCGATGCTAAAGACCATC
....((((..(((((((((..((((((((.((((.......(((((((.((((((..(((.((....)).)))..))))))(((.((((((((((((...)))).
)))))))...))).))))))).)))).))))))))..)))))))))..)))))....
*********************************************************************************************************
*************************TCTTGTGTCCAACACCGATGCT**********
```

novel_mir_127

```
TTTATTAAGCTCAATCCGGATTAAGTTATTTCCTCTTATATTTTGCTTGAGAGGATTGGTGTCGCAAGGTCCTTTTTGTGTTGCTACCAATCCTCTCAAGCCAAA
TGGTTTGAGCACTCCAAACCTGCACATAAACAAAAAAGAATAATAACCACAAAAAAGACCTTGAGATACCAATCCTCTCAAGCAAAATACAAGGGGAATAACTTA
ATCTGGATTAAGCTTAATAAAG
(((((((((((.(((((((((((((((((((((.(((.((((((((((((((((((((((((.((((((((.((((((((.(((..........((((((((...
.))))))))............)))......................))))))).))))))).)))))))))))))))))))))))))).)))))))))))))))
))))))))).))))))))))).
**********TCAATCCGGATTAAGTTATTTC*************************************************************************
```

****

****

novel_mir_14

AGTCCGACCGTCTGGTTTGAATGGACGGCCTGGCCTGACAAACAAATTAAAAAAATAAAAAAAATGGTCAAAATCGTTGAACGGGTTGGCAGTTCGACCACTTGG

GTCAATGGATTGTTGACTTTTTCAATAGTTTTTGGCCAAAGACTGGATTGGGGTAAAATAGAACCAGTTGCTTGGACATTCCTGCGCCGAATGGTCCAACCATCC

GGGTCAATGGCAGTTCGAAAACCTTGCTGGGGCC

.((((.(..((..(((((...(((((.((((((((((...........................(((((((((.(((((((.(((((((((((((((((.....)

)))...))))))))))))).))))))).).))))))))..((((((.((...........)).))))))..((((((((((.......)))).)))))).....)

))))))..))).))))).)))))..))).)))).

****

****

TCAATGGCAGTTCGAAAACCT*******

novel_mir_15

TTTCCCTTACGCTTAGTTTGGAAGGAATACAAATACAAATATGTACAGTGCAAATATATACAAAATAATACAAAATCCAAGATTTTGCGCTCTACATATTTGCAT

TTCTACTCCTTCCAAACTAAGCCTTATGGGGACC

..(((((...((((((((((((((((.((.((((.((((((((((.((((((((.............................)))))))).)))))))))).))

)).)).)))))))))))))))).....)))))..

****

*TCTACTCCTTCCAAACTAAGCCT**********

novel_mir_16

AAAGTCTTCTGCATCGGTGACGGACACAAAACTTATTCATATTTATCCTCCATGATCCGGTAGCTGTCTCCTGTCTTATGGGCTTGGCTATGAAGTTCCATTTCC

ATAGTTTCTGGCTGGATAGACAAGTCTTGTGTCCAACACCGATGCTAAAGACCAT

...(((((..(((((((((..((((((((.((((.......(((((((.((((((..(((.((....)).)))..))))))(((.(((((((((((...)))).)

))))))...))).))))))).)))).))))))))..)))))))))..)))))...

****

************************TCTTGTGTCCAACACCGATGC**********

novel_mir_18

```
GGGTTCAAATCTGCCGCCGCTCTACTTCTCTGTGAAGAAGATGAACACCGTCACCAGATTTGAACTGTTCATCTTCTTCACAGAGAAGCAGAGCGGCGGCAGATCTGACC
.(((.((.(((((((((((((((.(((((((((((((((((((((((...(((.......)))..))))))))))))))))))))))).))))))))))))))).)))))
*******************************************************************************ACAGAGAAGCAGAGCGGCGGC**********
```

novel_mir_2

```
TCCTACCCACAGCACACTACAGCTGTACATTGGGAACATCTCCCATGAATGGTAACATGTAAATACGGAAGATGTTCCCAATGTACAGCTGTAGTGTCCTGTGGGTAGGT
.(((((((((((.((((((((((((((((((((((((((((((((((........)))).......)).))))))))))))))))))))))))))).)))))))))))).
*******************************************************************************AATGTACAGCTGTAGTGTCCT**********
```

novel_mir_22

```
AGATGAAGGCAAGGCCTTCATTCAAATTCCTAAGCAGTGTGGAACTTGAGACAACACTCATTAGTGAATGTAACACTTGAGTGTTGTCCCAAGTCTCACGTTTCT
CTGGAATTAGAATGAAGGCCTTGCCTTCATC
.((((((((((((((((((((((.((((((..((.(((((((.(((((.(((((((((((..((((.......))))))))))))))))))).))))).))))))).))
..)))))).))))))))))))))))))))))
*********************************************************************************************************
CTGGAATTAGAATGAAGGCCT**********
```

novel_mir_27

```
GGGTTTTGGAGTTAGGGGACGTTTCAGTCCAACCAAGATGGTTTCGGGTTGATCTCTGCTGCTGAATCGTCGGTTCATGAACACATCTGTCGGCACGGAGAAGAT
GTGTTTTTGATGAGAGGATTCAGGAGCTGAGTTCGCTTATGGCTGTCTCGCCTTGGACTGAAGGGTCACCTATTCTGAATGCC
.(((((((((((.(((.(((.(((((((((((...((((((((..((((.((.(((.(((.(((((((.((...(((.((((((((((.((......))..))))
)))))).)))...)).))))))).))).))).))))))..))))))))....))))))))))).))).))))))))))).)))
*********************************************************************************************************
****************************************************TTGGACTGAAGGGTCACCTAT**********
```

novel_mir_28

```
AGAAAGTGCAATAGCCTGTAGTAGACCGCCTATCAGACATAGAACCGGCCTAGTCAGCATCAGAATACCCTTCTATTTCAAATTTTCCGCCGCTCTTGTATATCA
GACTGCGACCCAGAGCCCCTTTTAAATACCTCACAATTCTCAGAACTGCACTCCAATGAGGAATCAGAGGTTTCTGCATGAATTGGCTTACCACACTAACCGCAA
ATGAGATGGCTGGTCTAGTAATGGTTAGGTAGTTCAG
```

...((.(((..(((((..((.(((((((((.(((...(((.....(((...((((((((((((((..........................(((((((((.....
...))))....))))).........(((((....((((((..............))))))....)))))))))).)))..))))))...........)))...
))).)))))).)))))).))..))))).))).))...
**********ATAGCCTGTAGTAGACCGCCT**
****
****

novel_mir_3

CAGATCTGCCGGAATCAAATCGGTGATAAGTCGGGGTTGACAAGAAACCAGCAGATCCAACTTATCACCGATTTGCTTTCGAATCAGGTCCA
..((((((.(((((.((((((((((((((((.(((((((.........))))...))).)))))))))))))))).)))))...))))))..
TTATCACCGATTTGCTTTCGA*******

novel_mir_30

TCAGTCAGAGCAAAATCCGTTCGAACAGCCTTTATAGCCTTGCATCGGGAATATCTATGATGGCTGTTCGAATGGACTTTGCTCTGACTGAA
((((((((((((((.(((((((((((((((.(((((((((......))).....)))))).))))))))))))))).)))))))))))))).
**********CAAAATCCGTTCGAACAGCCT***
GCTGTTCGAATGGACTTTGCT*******

novel_mir_31

GTTCAAATCTGTCGCCGCTCTACTTCTCTGTGAAGAAGATGAACACCCTCACCAAGTTGAACTGTTCATCAGATTTGAACTGTTCATCTTCTTCAGAGAAGCAGA
GCGGCGGCAGATCTGATC
..(((.(((((((((((((((.(((((((..((((((((((((((..................(((((.......)))))))))))))))))))))))))).)))
)))))))))))).)))..
***CAGAGAAGCAGA
GCGGCGGC**********

novel_mir_32

GCTTTGTAATTAAGGTCGAACGAGGAAGAGAGAAAAACACCCATCATGGTCATGGGTAATTTCGATCAGATGAAATTAAGGTGAGTGATTTCCATGTTTTGCTTG
TCAAGTGAACAGCCTTTAGCAGATCGCCCTCTGCTTCGTTGTCTGTTCGACCTTGAACATATGGG
.((.(((..((((((((((((((.((((((((.......(((((.......)))))(((((((........))))))).(((((.((.......(((((.((((.

..)))))))))........))..))))))))).)))).))....))))))))))))..))).)).

****

**********************************TTCGTTGTCTGTTCGACCTTG**********

novel_mir_33

CTCGGGAGTAGTGGGAATGGAGGGCTGGGACAATTCTCCTTTCCTATGCTCCTATCCTTCACTCTTTCTTTCCTGGAGGAGGGCCAAGGCCTAGGAGATCGACGA

ATTTTCCCACACCGCCCATTCCTACTACCTCCGA

.(((((.(((((((((((((.((..(((((.(((((((.(((((((.(((.....(((((.((((.........)))))))))....))).))))))).)).))

))).)))))..))..))))))))))))).)))))

****

TTTCCCACACCGCCCATTCCTA********

novel_mir_34

TAGGGATAGTTTGGGTATGGATGGGTGGGAAAGAGAACGTCAGGATTTTTTAGAAATCTCCTCTCTCTTTCCGATCCCACCCATACCTAAGCTCTCCCT

.(((((.(((((((((((((.(((((.(((((((((..(...(((((((...))))))).)..))))))))).).)))).))))))))))))).)))))

*************GGTATGGATGGGTGGGAAAGA***

TTTCCGATCCCACCCATACCTA*******

novel_mir_35

ACGGAGGCCTTCGTTCCCCAGTTTACTCTTGTGCAGACTGCAGAAGGCTTCCATGCTCTGCTCTAACAGGAGTAACCTGGAGAACGACGCCCTCTGTC

(((((((.(.((((((.((((.((((((((((..(((..(((((..((......)))))))))).)))))))))).)))).)))))).).))))))).

**********TCGTTCCCCAGTTTACTCTTG***

GGAGTAACCTGGAGAACGACG*******

novel_mir_36

TGAGGTTTGGGTGAAATGGAGCTCCCTTCTTTCCAACATAGTGAGGAATAGGGTGTTTACATCTGCTGGTTCATGAATACCTTTGGGTGCGCATTTCAAAGGGGT

ACGCGCAGTTTAAGGGTGGGCATGAACCAGGAGATGAGATTGCCTTCTTCTTTTCTATCTTGGATTGAAGGGAGCTCCAATGACGTCT

.((.(((.........(((((((((((((..(((((.((((.((((((.((((((.((.(((((.((((((((((..((((((((((((((((((((...)))))

..))))).))))))))))..)))))))))).))))).)).))))))).))))).)))).)))))..))))))))))))).)))..))).)).

****

CTTGGATTGAAGGGAGCTCC********

novel_mir_37

ACGGAGGCCTTCGTTCCCCATTTTACTCTTGTGCAGAAGGCTTCCATGCTCTGCTCTAACAGGAGTAACCTGGAGAACGACGCCCTCTGTC
(((((((.(.((((((.(((..(((((((((((((((..((......)))))))....))))))))))..))).)))))).).))))))).
**********TCGTTCCCCATTTTACTCTTG**
GGAGTAACCTGGAGAACGACG******

novel_mir_39

AAACACTACTGGGTCCCTAGTGTCTGGTTCACCTCGAGCCACTATGAAATCTTGCTAACTCTAAACGGAATGGATACCCTTAGGTTCACCATACTGGGTCATTGA
TGGCCATATTAAGTTAAATAGAAAGATTCATCTCAACCAAGTGGGGGATTGTTAGAATTCATTCAACCAAGAGTTCATAGTGGCTTGAGATGAATCAGACACTAG
GACCCAGTAGTGTCT
..((((((((((((((.((((((((((((((.((((((((((((((((.(((((.............((((((((...((..(((((.((.((((.(((....((
(((...((((......)))).......)))))...))).)))))))))))...)).))))))))...))))).))))))))))))))))).)))))))))))))))
)))))))))))))..
************GTCCCTAGTGTCTGGTTCACCTCG***
****
****
****
***AGATGAATCAGACACTAG
GACCC**********

novel_mir_40

AATCCGAGGCAGTATGCATTATACGTGTAATGCATGCTGCCTCGTAGATCTCCCGTCACTGCTCGTCGCCTTGCATGCATTATACGTGTAGTGCATGCTGCCTCG
TAGATC
.((((((((((((((((((((((((((((((((((((.((..((.((...............))))..))...)))))))))))))))))))))))))))))))))
..))).
TATACGTGTAGTGCATGCTGC**
******

novel_mir_41

CTTTGGCTTTTTTTTGCACAAAATGGCTGCCCTGAGACTTGAACCCTTGTTCTCATGTTTTCCAGTCCTGTGGCAAAAGCTATGGCTACAAAGGACATGTTATTG
TGGGGAGAACCGGGACCAGGAATGGAATTTTAAAATATGTAACATTGGCTCCATGGAGGTATGACTTGTCTTGTCTGCTATCCAGGGACCATTACTTGGTGTGAA
GGTAAAATCTCAGGCTGCCATGCATGAAAGGGTGGGTTTGACTCTGAAGGCAGCCACTTTGTGCTAAAAAATGCCAAAGG
(((((((.((((((.(((((((.((((((((.(.(((.((((((((.(.((.((((((....((((((((((((....)))))))((((((...........)))
))).(((((((...(((((((((((........................(((.((((((((.(((.......))))))).)))).)))))))).)))))).....
)))....)))).)))))....)))))).)).).)))))))).))).).)))))))).))))))).)))))).))))))).
**********TTTTTGCACAAAATGGCTGCC**
****
****

novel_mir_42

GTGGAACGCCTCGCGTCACACCTCCGCGTTTTAAACACTGGTTCAAATATGGCTAATGGAGTACATAAGACTGCAACAACTAGTGCTTCTCCTCATATGGGACCA
ATGTTTTAAAATGTGGAGGTGTAAACAAGGTGTTCCACT
(((((((((((...((.((((((((((((((((((((.((((((..((((((.....(((((((..................)))))))...)))))).))))))
.)).)))))))))))))))))))..)).))))))))))).
**********TCGCGTCACACCTCCGCGTTT**
****

novel_mir_43

TGTTGAGAGATCTTGCTCAAGACCGCGCAGCTGATTCATGTAACACTGCATCGTGGTAGGTGTCTATCGCTGCTCAAAAACTAGTGTGATGACAGTGAAGCCTGCCGGCCGGACGG
ACACCGATGACTCTTTCAACAACCGATCACTGATGGTAGCCCTCTACCTCGATGCGCTGTTCATGTGCAGGCTGGCTGTGTGGTCTTGCTCAAGATCTCACAGCAG
(((((.(((((((((..((((((((((((((((...((((.((((.(((((((.(((((((((((.((((((.(((...((....))..))))))))).(((....))).....))
)))))..(((.....)))...(((.(((...)))))).......)))).))))))).))))))))......).)))))))))))))))..))))))))).))))).
**********TCTTGCTCAAGACCGCGCAGC***
****

novel_mir_45

AGGAGAGGTTTCACCCACCACCGGCCATCTCCGGCAACCACTGGCAATAGTATACATTGTAACAGGTGTGCATTTTTTGCTCTCTTCTATCTACTCTATTTTTTT

ATATTTGCATTTTTGCCGATTTTTTCTTCTTACTGATCATCATATAAAACATGAAAATGGTAGCTTTTATTTCCTTCCGTGTTTACTGTTGTTGTTGGTCGCCGG
AAATGGCCGGCGGTGGGTGAAACCTCCCCTT
(((.(((((((((((((((.((((((((.((((((.((((.(((((((((((.((((......(((.(.(((.....))).).)))(((((..........((((
((((..(((....))).((((.............))))...)))))))).........)))))..............)))).))))))))))).)))).)))))
).)))))))).))))))))))))))).))).
**********TCACCCACCACCGGCCATCTC***
****
****

novel_mir_46

CCGGGTTCAAACAATGCAAAGCCCGTCTAACACACATCATATAAATCCACAGGTATGCGGATTTGTTCCTGATGAAAGGGTGAATATGCTATTCGGAGGGTGTTT
GAACGGGCCTTGCACTGACCGAACCCGA
.(((((((...((.(((((.(((((((.(((((.(((((.((((((((.((....)).))))))))...)))))......((((((...)))))).....))))).
).)))))).))))).))...))))))).
**TTT
GAACGGGCCTTGCACTGA**********

novel_mir_48

TGAACTCTTTAACTGTTCAGCGGGCTGTGAAATCGATGACAAAATTTTATGTCTATAATAAATTTATGGGCCCTATTATAAAAGATGCAGCCCCATGTATTTAAT
TACTGGTGTGAAACCATATAATTGTTTCCACAGCCTTCTGAACAGCTAAAACGTTC
.((((..((((.((((((((.((((((((.((.((((.......(((((((((..((((((((.(((((((..((((.....))))..).))))))).)))).))
))..))))))))).......)))).)).)))))))).)))))))).))))..))))
****
*************************TTCCACAGCCTTCTGAACAGC**********

novel_mir_49

AACGGCTTGAGAGAGCTTTCTTCAGTCCACTCATGGATGGGCTAAGGTGTTGAATTAGCTGCCGACTCATTCATTCAAACACTCAGTAGAAAGGGGATATTTTTT
ACTACTGTGAATGTGTGAATGATGCGGGAGATAATTGCCATTCTTCCTCCTCTATGCTTGGACTGAAGGGAGCTCCCTTTTTCTGT
.((((...(((.((((((((((((((((..((((((.(((..((((.....(((((.((.(((..((((((((.((..(((..((((((((......))))))

)))...)))..)).))))))))..))).)).))))).....))))..)))))))))..))))))))))))))))).)))...))))
****
GCTTGGACTGAAGGGAGCTCC********

novel_mir_50

GTAGCCCGCACTTGGTACAGGAACTTGGCCCCCGGGTCGATAAGATGCTGGGCCTAGTTCGTGCAGGTGTTCACCGCACACTTTGTCACTAGCAAGCGGTGCCCC
.......(((((((...((.(((((.(((((..(.(((.....))).).))))).))))).))))))))).((((((...((........))...))))))....
**********CTTGGTACAGGAACTTGGCCC**

novel_mir_52

TTCATGGTGATCATTTTGATCATGAGGGTACTTGTCAATGTTTTTGTGGAGCCAGCAAAGTGTTTTTTTACTGCCTTCAAATAATCCATTGCGCTTTCCTTTTCA
GGAATTGCACCATGGATTGCAATAGAAATGGTGCTCCTTATGATCATGAGACTCATGCA
..(((((...(((...((((((((((((((((.......((((((((((((.(((.((((.....)))).))).)))))..((((((((.(.((.(((((....)
))))..)).).))))))))...))))))))))))).))))))))))))))...)))))..
**********TCATTTTGATCATGAGGGTACT***
****

novel_mir_53

ATCCAATTGATGAGAAAACATGATATAAGGACAACATCACTCCAAAAACGCTTTGGAACATTCATATGAAGCATAATTGACTGAGCAATTACAAGTGAGTGTCGG
TTTTTCCGTTTAGCCACACCATTTTGTTGTGGGGTATAAGAACAAGAAGTTTGGTGATATACCCCATGGTGCTCAAAGTATTGCTGAAATGTTCCAAAGCGTTTT
TGGAGTGATGTTGTCCTTATATCATGTTTTCTCATCAATCGGATG
((((.((......((((..(((...((((((...(((((((.((((.((
((.........)))))))))).))))).(((((((((....(((.....)))....)))))))))..)))))).)))..))))..))))))))))))))))))))
))))))))))))))))))))))))))))))))))))))).)))).
**********TGAGAAAACATGATATAAGGA**
****
****

novel_mir_54

TTATGGAATTTGATGTCCTCGAATTGTTTGCCGTTGTTAGAGATGAGCACTCGGGGTAATCCAAAACAGAAAACTATATTCTTCCAGAAGAAGTTCTGCACACTT

CGCGCTGTGATCGTTGCGAGAGGTTCGACCTCCACCCACTTAGTGAAATAATCAATTGCTACCACTAAGAACCTCCTCTATCTTGTCGCTGGTGGAAACGGTCTG
AGTATATCGATCCCCCA
....(((..(((((((.((((.(((((((((((.((...((((((((.....(((((.........((((..(((...((((...))))..)))))))......(
((((.((....)).)))))(((((...)))))......(((((((.................))))))).)))))))).)))))..)).))))..))))))).))
)).))))))))))....
**********TGATGTCCTCGAATTGTTTGCC***
****
****

novel_mir_61
CAGCAACACTCGAGAAGAATTTCCGGATGAACAACTATACTCAATTCAGGAATATTCATCTCCTTGGTATGCTGA
(((((((....((((.((((((((((((((..........)).)))).)))).)))).)))).....)).))))).
**********CGAGAAGAATTTCCGGATGAAC***

novel_mir_62
ACTTGAGATGATTTGAGGGATCCAAATTTTGACTTTTTTATCTTTGTGCCCTTAACATTGGATAGATTGCTTTTTCTTTTTTCTCAATCAAGCAAATGATGAATA
ACTCGGATTCCCCTTCAAG
.((((((..((((((((..((.((...((((.(((...(((((.(((.......)))..)))))(((((...............))))))))))))...)).)).
.))))))))....))))))
***GCAAATGATGAATA
ACTCGGATT**********

novel_mir_65
GCTGGAAGTTTTTGGGATGGGAGCCTTGGGAAGCAGAGGCGAGTGAGAGACCGAGATAAATTCATTTTCTCTCGTTCTCTTTCTCTTCTTCTGCTATGCTTTTCT
TCCCAAGGCCTCCCATGCCGATGATTTCCCGTG
((.(((((((.((((.(((((((((((((((((((((((.(((.((((((.(((((.(((.....))).))))).)))))).))).))))))))..........
)))))))).))))))).)))).))))))).)).
**********TTTGGGATGGGAGCCTTGGGA**
****

novel_mir_66

GTGGTCCTTTATACCTGACTCCCGTCGTTGATAATTTCTGAGGTGTAATTTGTTGGCGTTAAATGAAATCTAGTCTTTTGGCTTCCAAATAAAAGAAATTACAAA
AAAAAAAAACTCATGAAGGATGTATCATTAATTAGTAGTTAAGTATCATTAAACTCATAATTTTAGCATCTGGTGGACGTGCCGGAGTGGTTATCGGGCATG
...((((..((.(((..(((((.((((((.((..((((((((.((((((((.((((.((((((.((.......)).))))))..)))......).))))))))..
.........)))).))))((((......(((((..((((((......)).))))..)))))...)))))..)).)))).)).))))))))))..))))...
****
GTGGACGTGCCGGAGTGGTT********

novel_mir_68

AGTGGTTGAGGTGTCGTCCAGCTTGAGCTTGATTCAAATTAGAGACGCGAGGTGTTGAGCTAAGGCCTCCCTTAGTTGACAAGGTGCTGAGTTGAGGTCTCCCTCAGTTG
TCGAGGTGCTGAGCCGAGACCTCCCTTAGTGACCGAGGTGTCAAGCCGATGTCTCCCTCAGTGGTTGAAGTGTCGTCTAGCTCAAGCTCGATTCGAATTAA
.....((((...((((...((((((((((.(((....(((..(..((((((((((.((((((((....))))))))))).(((((((((..((((((((.((((((..
.......))))))..)))))))).)))))).)))((((((((.....)))))))))))).)))..)..)))...))).))))))))))))))))).....
****
TGTCGTCTAGCTCAAGCTCGA********

novel_mir_69

CCATTCGAAGAAAATTTAGTTTCAGATCAGAGATTGGCAATGGAAAGTTTCTTCAATGTGGTTATGTTAAGCTTCTCTTTTTGTTTAGATGTTCTTATTGTTTTC
CTCTGTGGTGATGAAATATGAAAATTCTTCGCATG
.(((.(((((((..((((((((((.((((.(((........((((((......(((((.((.(((.((((((..........)))))))).)).))))))))))
)))).))))..)))))).))))..))))))).)))
****
TGTGGTGATGAAATATGAAAAT*******

novel_mir_7

AGAACGTCCCCCTTCGAGTTCCGCCAGTCAACGCAGATATCCTCCGGCGGCGTCGACTGGAACGATCGTCGGCGTCGACTGGCGGAACTCGAAGGGGGCAAGCTC
.......((((((((((((((((((((.((((.((((((.(((((((....)).)))))..))).))).)))).)))))))))))))))))))))).......
TCGACTGGCGGAACTCGAAGG********

novel_mir_70

TTCTTGATTTATGACTGAGATTTCTGTGCAGTGGCTTCTGGCCTACATTGGCCGTATTTCTGCAGAGGATTCCAATTATTGGTTGGGTG

..(((((((.((((.((.((.((((.(((((.......(((((......))))).....))))).)))).)))).)))).)))))))..

**********ATGACTGAGATTTCTGTGCAG**

novel_mir_71

GATATGGCCCAGGCCTGCAGTCAGGGAACACTAAACTTTTAAAAAGACTGGGTCAGGTTCAAAATTATAAGGATAGGCTAATGGTAAAAGTGAGGGGTACTGGTG

GGCCCAGTCTTATCTATGGGCTTACTTGCTGTTGGAGTCTTATTTCCAGGCCCATCCACTTTAAGTTTAAGACTGGGCCCACCAGTACCCCTCACTTCTACCGTT

GGCCCATCCTTATAATTTTGAACCTGACCCAGTCTTTTTAAAAGTTTAGTATTGGTTCCCTGACTGCAGGCCTAGGCCATAT

.((((((((.((.(((((((((((.((((((((((((((((((

((((((((((((.(((((((((..........(((((......))))))))))))........))..))))))))))))))))))))))))))))))).)))))))

)))).)))....))))))))))))))))))).))))))))

****

****

TTGGTTCCCTGACTGCAGGCC*******

novel_mir_72

TTCATCTTTTCGGCCATTCGTAAGGACATTTCTTCGAGATCTAGTGGGTCCAAGTCTAGGAACTTACTTCGAAGCTTGTCCATGTAGGATTCAAAGATGGG

.((((((((....((...(((..(((((...((((((.....(((((((((.......)).)))))))))))))..))))))))..))....)))))))).

TCGAAGCTTGTCCATGTAGGATT********

novel_mir_73

CATCTATCGGTTGAGAGCAACTGTGACTAGAGAGAAGAGTCTCCTTTGTATGCTCTGTTTCAGTTTTGCAGTTCAACAGAGAGAGA

..(((.((.((((((.(((((((.(((.((((...((((....)))).....))))))).))))..)))..)))))).)).)))..

**********TTGAGAGCAACTGTGACTAGA***

novel_mir_74

GCTACAAGGATCATCTGATGAGAAAACTGCAACAAAATGACATCTCATGATTTGTTGTATTTAAGTCTGATGATCTCTGTTTG

...(((.((((((((.(((..(((...(((((((((((((....))))..))))))))))))..))).)))))))).)))...

**********TCATCTGATGAGAAAACTGCAAC**

novel_mir_75

CGGAGACAGTTGTGTTACTGGAAATGTGGGAAATCACATGGGTGCATGGAGCCAACTGTTTCAGCTACATAACCCCACCACTTTTTGTGAAAGATCTTATCCACT
CATCTTACGAGTTTTTCCTTTGGCCTCTATCACACAAGGTTAGTAACCATTTTAGGTTAAACCTTTCTGTCCAGTAAGTGTTCACCCATCATGTTCGTGAATGCC
AAAGTATCGTGAGAGAGTCTTTCATTTTAGTATAGGCCCCTCCCACATCGGTCTATGACGCCTCTATCATCTC
.(((((.((..((((((.((((.((((((((........((((((.((((((.....))))))))......)))).(((((....((((((((.........((
(.((((((((.......((((((((.......(((.((((((..(((((......))))))))))..)))..........((((((...........))))))))
))))..))))))))))))))))))...)))...))....))))))))...))))))))))..)).)).))).
****
****
CCACATCGGTCTATGACGCCT********

novel_mir_76

TCGATGATGGTCTTACGGGTGTCTTCACCTCCGGAGTTGAAGAACATTATCGGAGATTCGTCGTATTTCCATCGGAGGTGAAGGCCACCCGCTAGATCTTCATCGAT
((((((.(((((..((((((((((((((((((.(((....)))......((((((.......)))))).)))))))))))))).)))))).)))))).))))))).
**********TCTTACGGGTGTCTTCACCTC**

novel_mir_77

TGGTGTTTCGATGGCATGTGTTGATGGGCTCTGTATCTCTCGCATTTCTTGTTATCTCTTGGTGACCCTTTTCGGATGTAAATGTTGGTGGTTGCTTTCTGTGCT
TAGTGGGGCCTTTGGGTGCTGCTTTTCTCGGCCATTCTACATGTCATTGATGCGTCG
.(((((.((((((((((((..((..((((....(((.((.((((((...((((((....))))))((.....))....)))))).)).))).((.......)).
.(((((.(((....))).)))))......))))..))))))))))))))).))))).
**********ATGGCATGTGTTGATGGGCTC**
****

novel_mir_78

TGCCACTGCTTTCTCAGTCGGGCACTCTGGCTTTTTCACTAAACTACCTAAAATGAAAGAGCCAGAGTGCCCGACTGAGAAAGCAGTGGCAA
((((((((((((((((((((((((((((((((((((((...............)))))))))))))))))))))))))))))))))))))).
**********TTCTCAGTCGGGCACTCTGGC***

novel_mir_8

TTTCCCCTCTTCCAACTCCCTCTCCGGCACCAACAATCGCTTCGACTAGTTCACCGTCACCAAGTACTCTCCAAATTAGAGAAGCAAGGTGCTCCTCTGATTTGAAATGA

AAAAACCAAACATCAAAACAAAATACGAGGGTTGGAGAGTACCTAGTGACGGTGAACTAGCTGGAGTGATTGCCGATGCCGAAGGGGGAGTTGAAAGAGGAAAAA
.....((((((.(((((((((((.(((((.(..((((((((((.((((((((((((((...((((((((((((((((((.((((...)))).))))))))))).......
...((((.....................)))).))))))))...))))))))))))))).))))))))))..).))))).))))))))))).))))))).....
**********TCCAACTCCCTCTCCGGCACC**
****

novel_mir_80
TACTGCGTTGTATGGTAGGCAGATTGTCTTAGTGGATCTAGACCGTTCTCTTCGATGGATACTGTGGGATGATGAATATCTTGTGCGAACGCAGCA
..((((((((((((((..((..((((((((..(..((((...((.......)).))))..)..))))))))))..))))..)))).))))))))..
GTGGGATGATGAATATCTTGTGC*******

novel_mir_81
TTTGTGTGAAAGTTATGAGTGGGCCACTTAGTCCAAAAGCCAAAGAGAGTGCTCAAGGAATTAAACTGATTTGTGGAAGCAAAGAATTCAATTGCAAGGCCACAT
GTGGCTAGGAAGGAGACTAGGCGTGTGACAAGTGGTATCAGAGCCTTTACTACCTC
...(((((((.(((.(((....((((((((.(((((....((...(((....)))..))..............(((((..((((.........))))....))))).
.((.((((.........)))).)))).))))))))))).))).))).))))).))...
****
*************************TGACAAGTGGTATCAGAGCCT**********

novel_mir_82
TTCAGCGGGCTGTGGAACCAATGATGAAGTTCCACGCCAAGTAATGAATTCATGGGCCCATGTATTTTATTCCTGACGTTGAGCCTTATCATTCGTTCCACAGTC
CGCTGAAC
(((((((((((((((((.((((((((.((((.(((.((.(.((((((.(((((....)))).).))))))).)).))).)))).)))))))).))))))))))
))))))).
**********TGTGGAACCAATGATGAAGTT**
****

novel_mir_83
TGGTTACGTGTCGATTCGAGCTGCTGGTTCCGAACTGGTACTTCGGCCTTTTAGTTCCGAACTGGCTTGAATACCAAGGTGGCTC
.((((((.(....(((((((((...(((((.((((((((......)))....))))).))))))))))))))....).)))))).

TTCCGAACTGGCTTGAATACC********

novel_mir_84

TTAAGCGAGACGGGATCAACTCTTCACATAGGGCGCATTACACCGATCTTATACATCCCACCATATTTATACAATATTATTCATTCATGGGAGATGTTATCAATG
CATAATGAGATGAACATGTAAAGTCGGTGTGAGGCGCCCTATGTGAGGAGCAGATCCCAATTCGCTTTA

..(((((((..((((((..(((((((((((((((((.((((((((((.(((((((((.(.((((......................))))).))))(((((.((.
....))..)))))...))))).)))))))))).)))))))))))))))))..))))))..)))))))..

**********CGGGATCAACTCTTCACATAG**
****

novel_mir_85

CTGCTGGTAGTCCGCTGCCAAGATGCTGAACAAGTTCTCTGTGAGCAGGTGCTCGAACTTAGAGTGTCGAGGTGCTGACTTGACTGGCAA

.(((((((((((.((.(((..((((((....((((((.....((((....))))))))))..))))))..))))).))))..))))))).

TAGAGTGTCGAGGTGCTGACT*******

novel_mir_87

TAGTGACAGGATCAGCCACTTTGTCGGACAAAGGCAATGATCGGGGATGCACTCTCACTAATGGAAACCAGGACTGCCGGCAACCGATTTCAATCTTTGAGATAT
GTTTTGCAAAGTATGTTTTATATGAGATATAAAAGTATATATATTATGGTTTGTAAAATGTTTGCAAAAATGTTTTAATTCCCAGGATCCATATAGGTAGAGAGT
GCAACACTGTTCCTTACAGCCGAGGGAGAGGTTCTCTCTTGTCGGGT

...((((((((..((((.((((.((((...((((....((.(((.(.((((((((((((.(((((..(..(((....(((...)))(((((((...)))))))((
((((((((((.((((...((((((...........))))))...)))).))))))))))))..(((....)))......)))..)..)))))...))).))))))
))).).)))))))))....)))).)))).))))...))))))))...

**********ATCAGCCACTTTGTCGGACAAAG**
****
****

novel_mir_9

ATAGAGAGAAAGAGAGATAGAGAGAAGGCGAAGGGAATTTAAATCCCACAACCCTTCGATTCCCTCTTTCTCTTTTTAGCTCTA

.(((((..(((((((((.((((.(((..(((((((................))))))).))).)))).)))))))))..)))))

CTTCGATTCCCTCTTTCTCTT*******

novel_mir_91

GATCAGATTTGCCGCCGCTCTGCTTCTCTGTGAAGAAGATGAACAGTTCAATCTGGTGAACAGTTCAACTTGGTGACGGTGTTCATCTTCTTCATAGAGAAGTAG
AGCGGCGGCAGATTTGAAC

..(((.(...(((.((((((...))).))).)))...).))))))))))))))))))))))))))
)))))))))))))))))..

**TAGAGAAGTAG
AGCGGCGGC**********

novel_mir_92

TTTCATGTTTTTCCACAGCTTTCTTGAACGTAAAGAGTCGAAGCCAATGACGAGAATGGTGTTGTAATTCAACTGTTCAAGTTCAAGATAGCTGTGGGAAGAGATGGAAG

((((((.(((((((((((((.((((((((...((.(((.(((..((((..(......)..))))...))).))).))...)))))))).))))))))))))).)))))).

**********TTCCACAGCTTTCTTGAACGT***

novel_mir_94

TAATCTAATCTGCCGAATGGATAAGACTGTTGCTGCCGAATTGGATAGGATGAGAACACTGGATCNATAAGATAAGATAATCTAATCTACTAACTTAATAAGATA
AGATAAGAATAACAAGATAGTCTAATCTAATCTGCCGAATGGATAA

..(((((.((.((.((.(((((.((((((((.(((((.....)).)))(((.((....))..)))....((((.(((...))).)))).....((((......))
)).............)))))))).))))).)).)).)).)))))..

**********TGCCGAATGGATAAGACTGTT**
****

novel_mir_96

GGCAATCTCCTCGAGAGAGGACTACTGTTGTCGATGATCTCCCAGTATTCGCTCCAAGCGCAGATGTAGACAGGAAGCATAATACTCCACGTAGCCGATCGTCGG
GGAAGTGGGTCCATATCATTCCTGTGATTGTGTTAGTTTGCAACTTGATACTCTGGTGGTTCTCTCATCCAGGTACGTTTGTA

.((((...(((.(((((((((((((((..(((((..((......))..)))...((((.((((((....(((((((..(((....(((((....((((...))))
....)))))....)))..)))))))..........))))))..))))..))..)))))))))))))).)).))).....)))).

**********TCGAGAGAGGACTACTGTTGT**
****

Additional file 1: Figure S4 Target plats (T-plots) of identified known and novel miRNA targets using degradome sequencing. The red lines indicate signatures consistent with miRNA-directed cleavage.

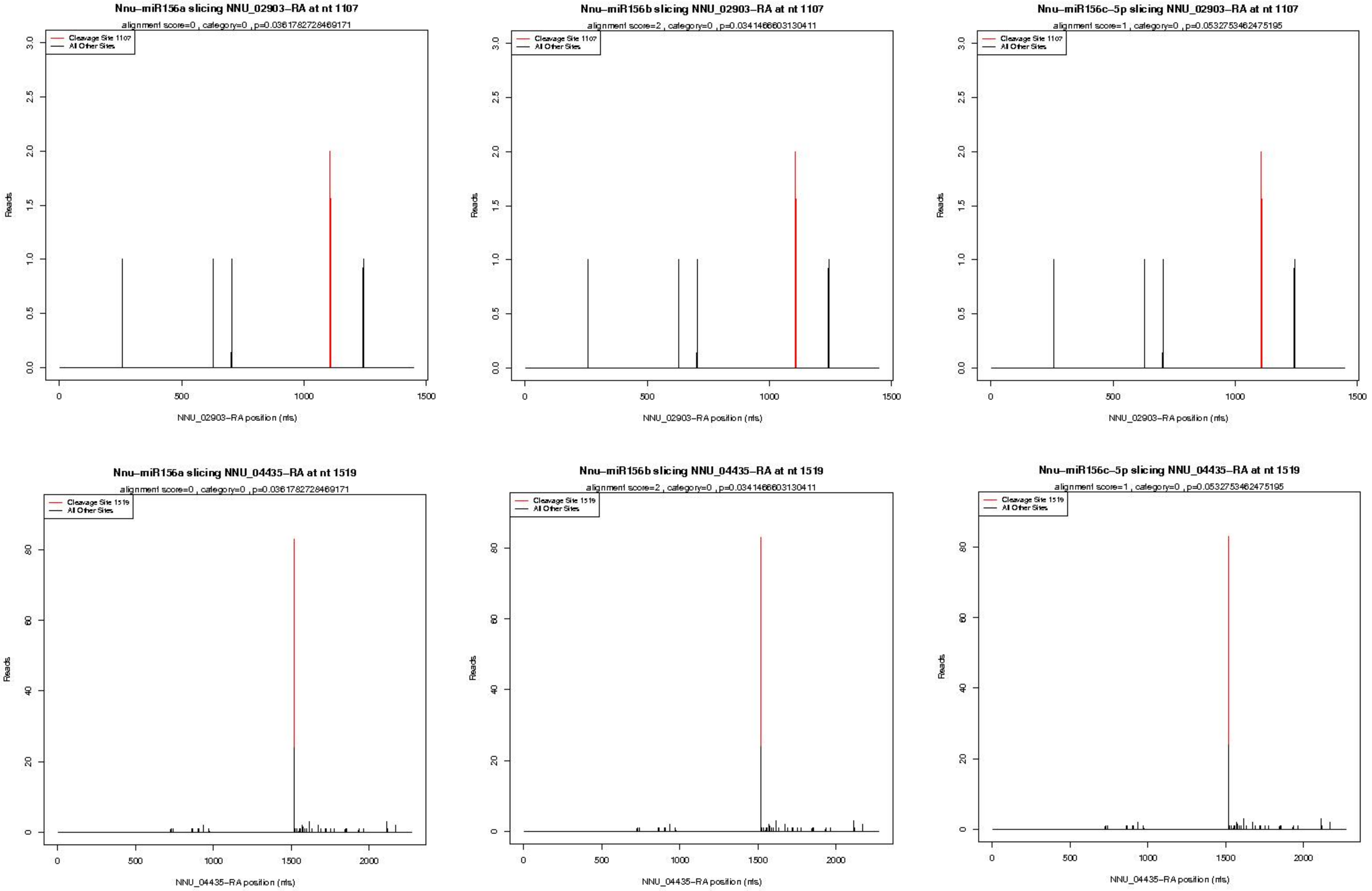

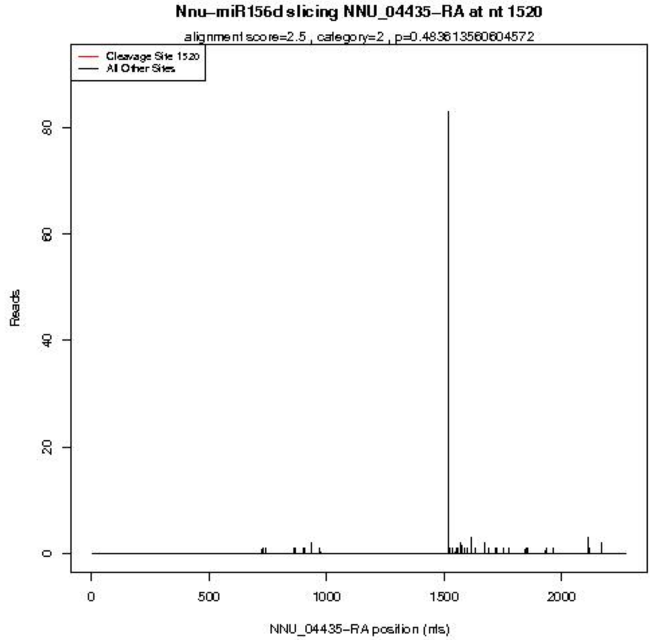
Nnu−miR156d slicing NNU_04435−RA at nt 1520
alignment score=2.5 , category=2 , p=0.483613560604572
Cleavage Site 1520
All Other Sites
Reads
NNU_04435−RA position (nts)

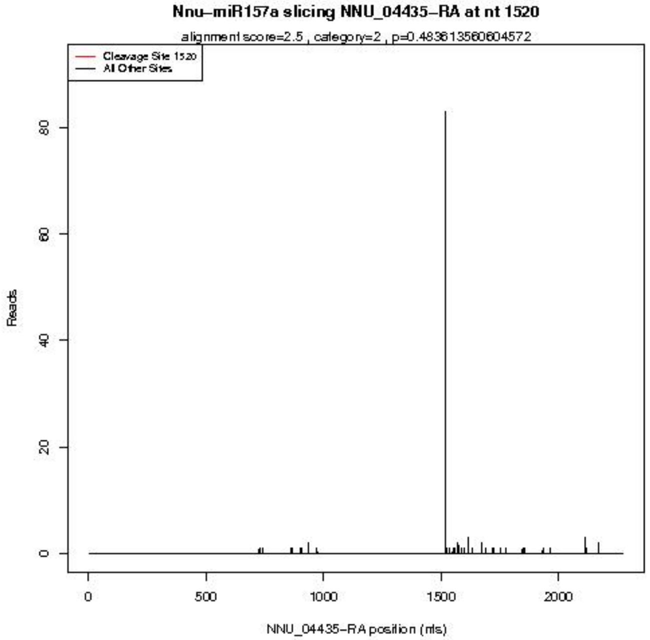
Nnu−miR157a slicing NNU_04435−RA at nt 1520
alignment score=2.5 , category=2 , p=0.483613560604572
Cleavage Site 1520
All Other Sites
Reads
NNU_04435−RA position (nts)

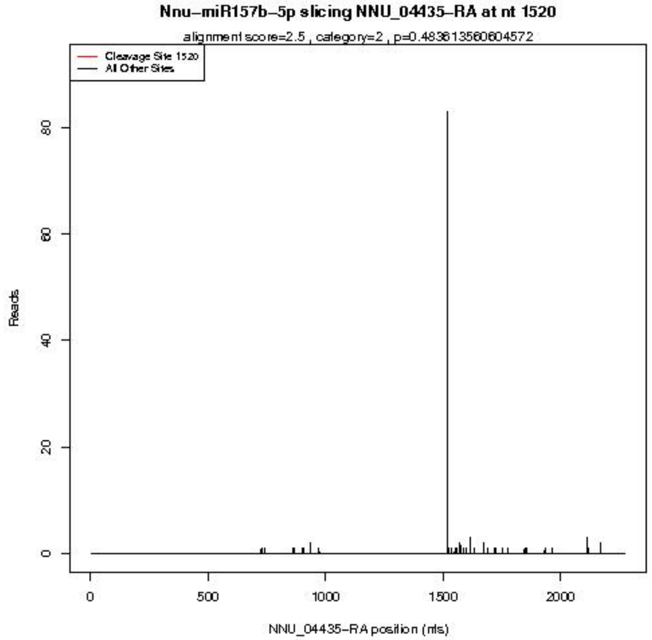
Nnu−miR157b−5p slicing NNU_04435−RA at nt 1520
alignment score=2.5 , category=2 , p=0.483613560604572
Cleavage Site 1520
All Other Sites
Reads
NNU_04435−RA position (nts)

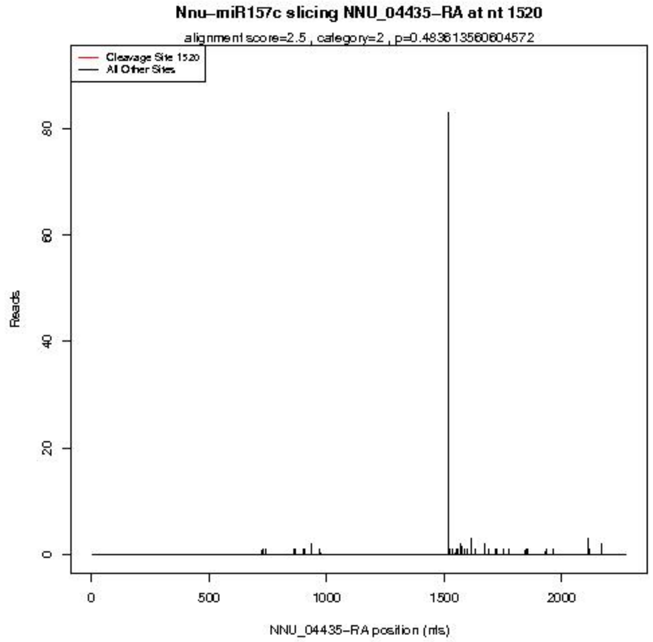
Nnu−miR157c slicing NNU_04435−RA at nt 1520
alignment score=2.5 , category=2 , p=0.483613560604572
Cleavage Site 1520
All Other Sites
Reads
NNU_04435−RA position (nts)

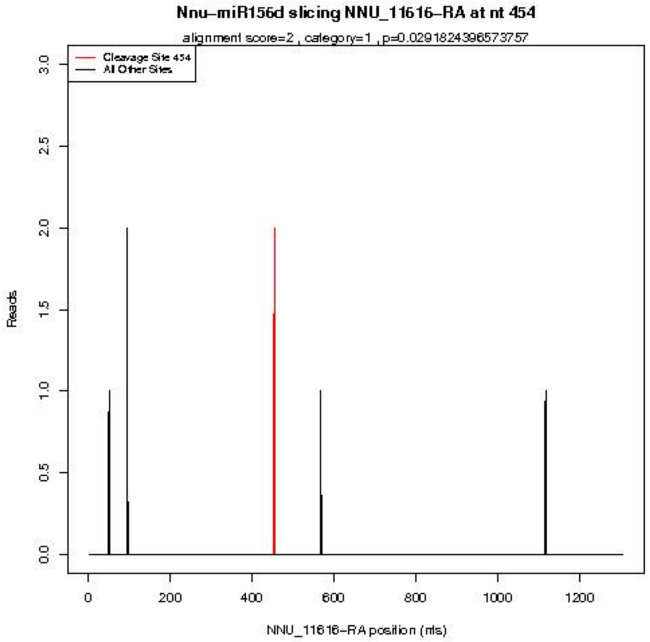
Nnu−miR156d slicing NNU_11616−RA at nt 454
alignment score=2 , category=1 , p=0.0291824396573757
Cleavage Site 454
All Other Sites
Reads
NNU_11616−RA position (nts)

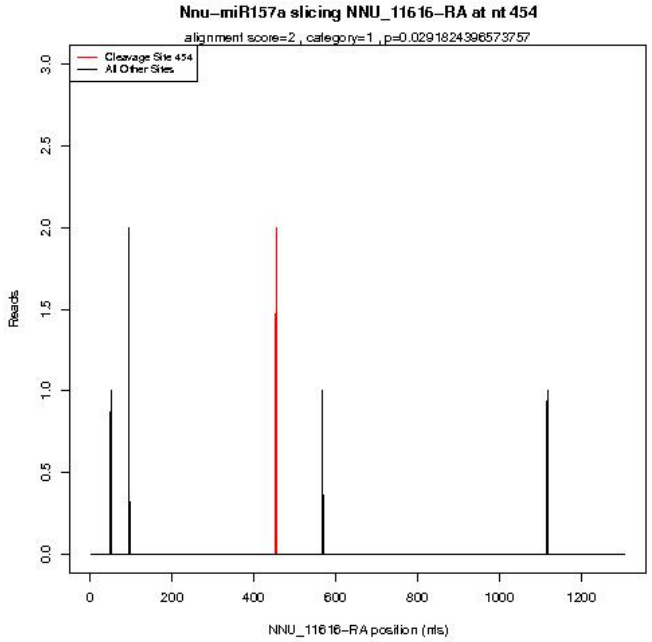
Nnu−miR157a slicing NNU_11616−RA at nt 454
alignment score=2 , category=1 , p=0.0291824396573757
Cleavage Site 454
All Other Sites
Reads
NNU_11616−RA position (nts)

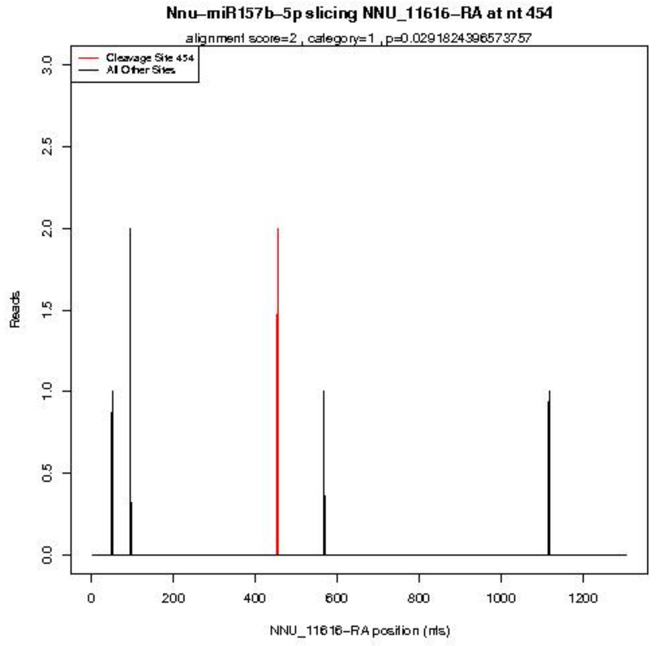
Nnu–miR157b–5p slicing NNU_11616–RA at nt 454
alignment score=2 , category=1 , p=0.0291824396573757
Cleavage Site 454
All Other Sites
Reads
NNU_11616–RA position (nts)

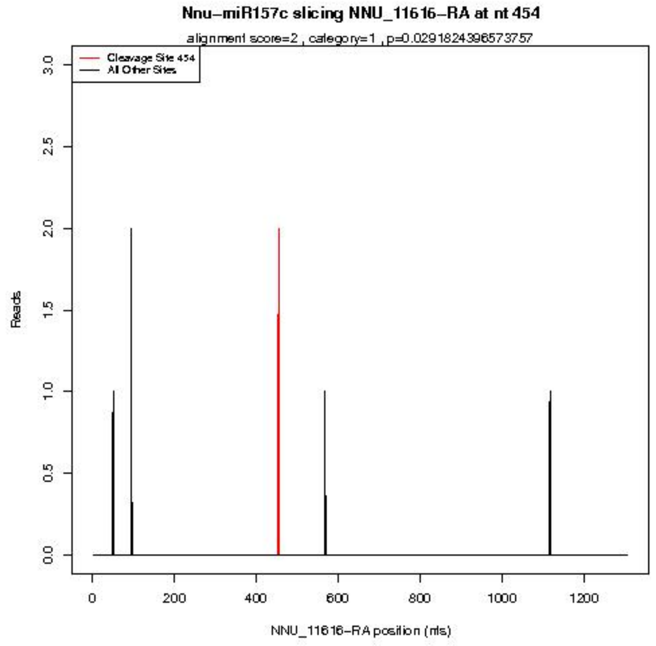
Nnu–miR157c slicing NNU_11616–RA at nt 454
alignment score=2 , category=1 , p=0.0291824396573757
Cleavage Site 454
All Other Sites
Reads
NNU_11616–RA position (nts)

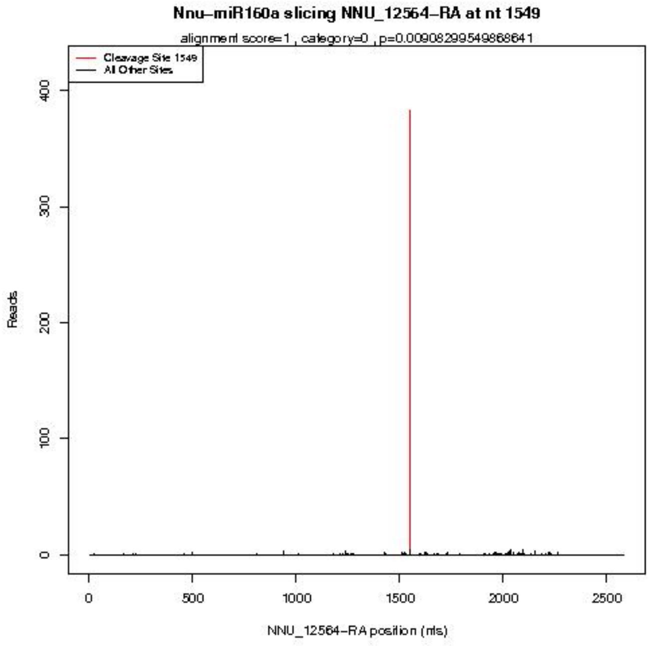
Nnu–miR160a slicing NNU_12564–RA at nt 1549
alignment score=1 , category=0 , p=0.00908299549868641
Cleavage Site 1549
All Other Sites
Reads
NNU_12564–RA position (nts)

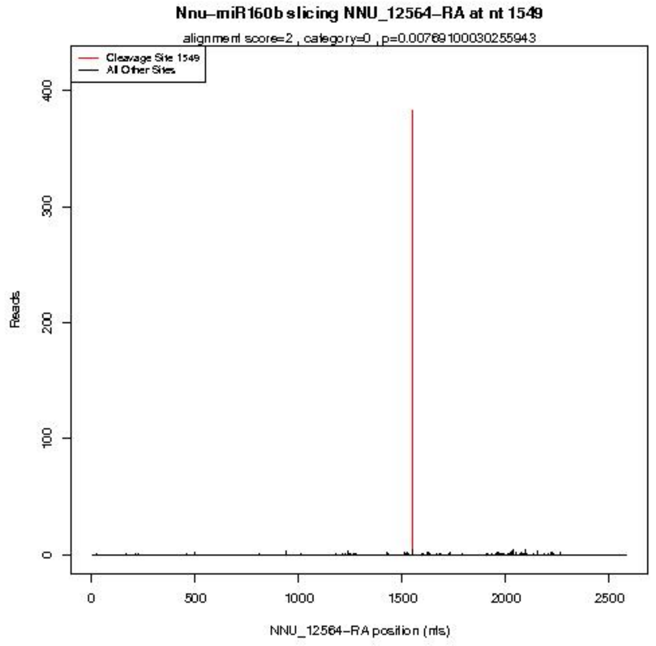
Nnu–miR160b slicing NNU_12564–RA at nt 1549
alignment score=2 , category=0 , p=0.00769100030255943
Cleavage Site 1549
All Other Sites
Reads
NNU_12564–RA position (nts)

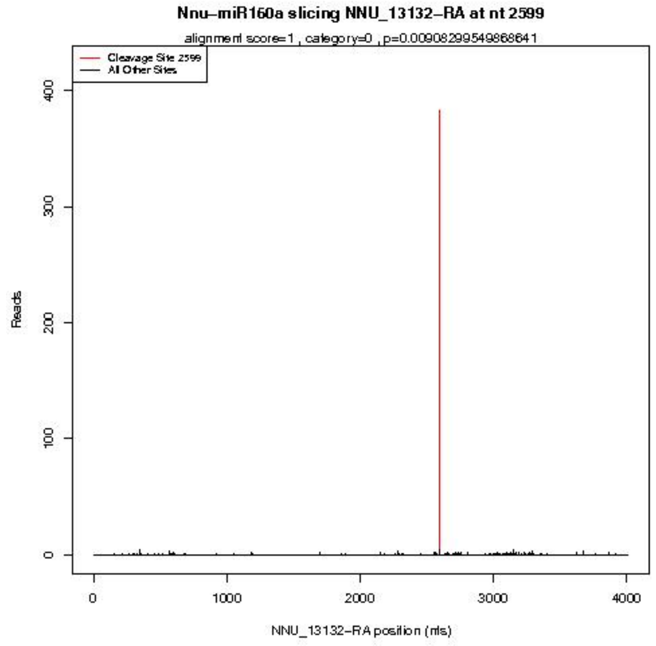
Nnu–miR160a slicing NNU_13132–RA at nt 2599
alignment score=1 , category=0 , p=0.00908299549868641
Cleavage Site 2599
All Other Sites
Reads
NNU_13132–RA position (nts)

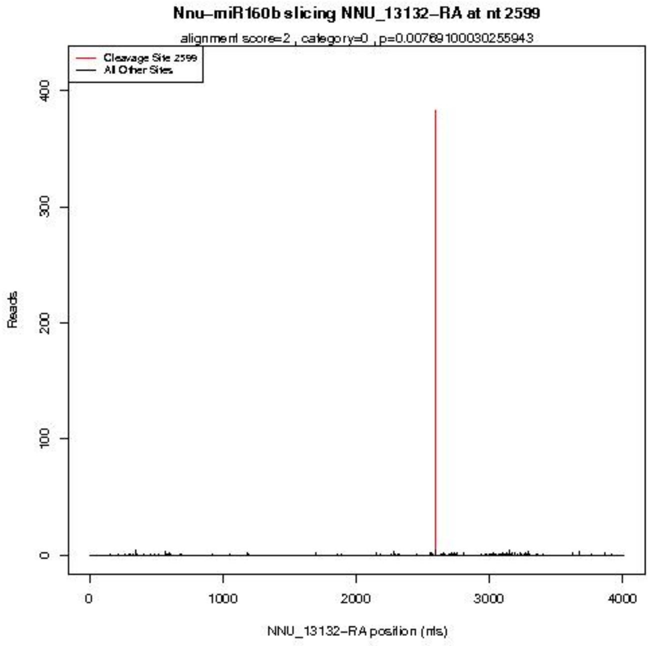
Nnu–miR160b slicing NNU_13132–RA at nt 2599
alignment score=2 , category=0 , p=0.00769100030255943
Cleavage Site 2599
All Other Sites
Reads
NNU_13132–RA position (nts)

Nnu-miR165a-5p slicing NNU_13147-RA at nt 2089

alignment score=1.5 , category=0 , p=0.0249512789812312

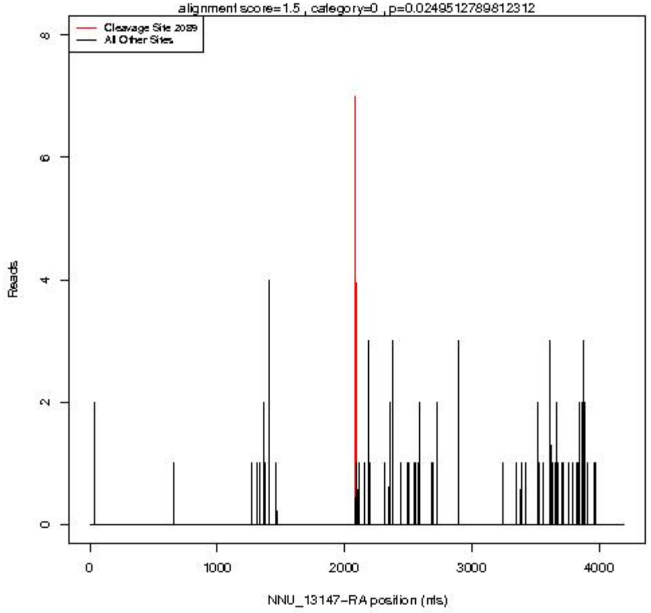

Nnu-miR166c slicing NNU_13147-RA at nt 2089

alignment score=3 , category=0 , p=0.0249512789812312

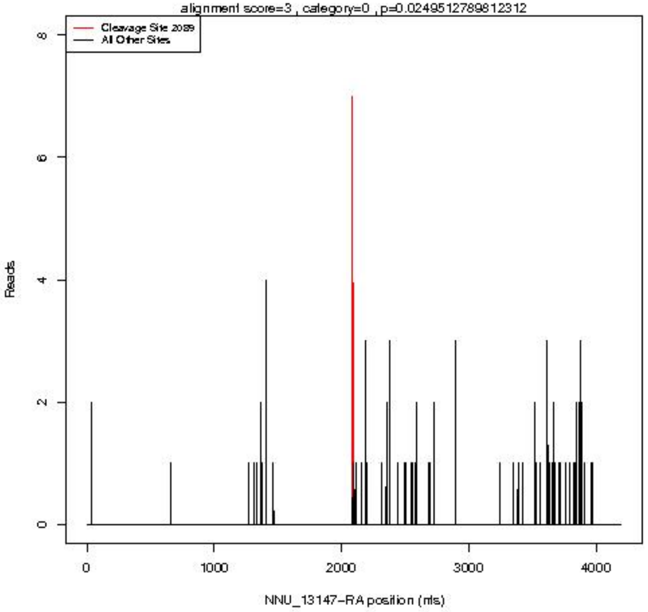

Nnu-miR164a-5p slicing NNU_25930-RA at nt 1344

alignment score=3 , category=4 , p=0.331183603537808

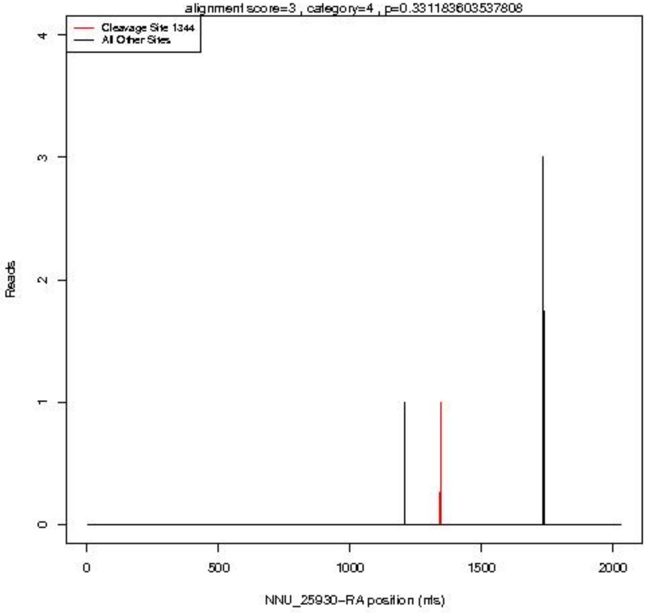

Nnu-miR165a-5p slicing NNU_26074-RA at nt 2321

alignment score=1.5 , category=0 , p=0.0249512789812312

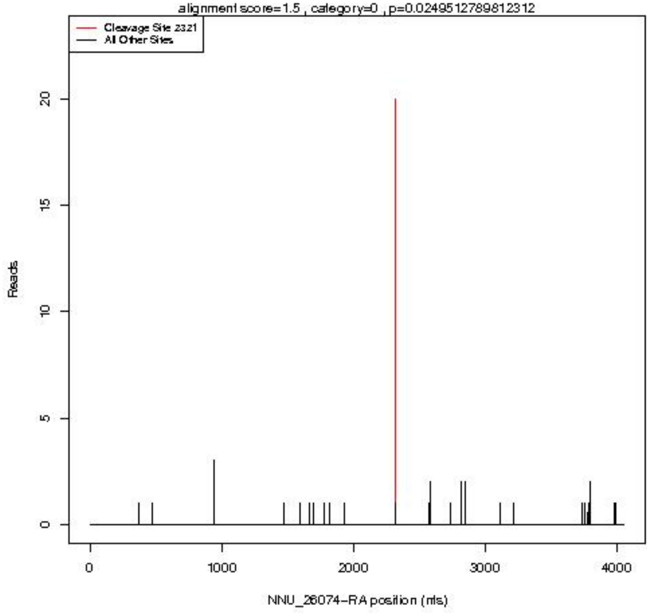

Nnu-miR166c slicing NNU_26074-RA at nt 2321

alignment score=3 , category=0 , p=0.0249512789812312

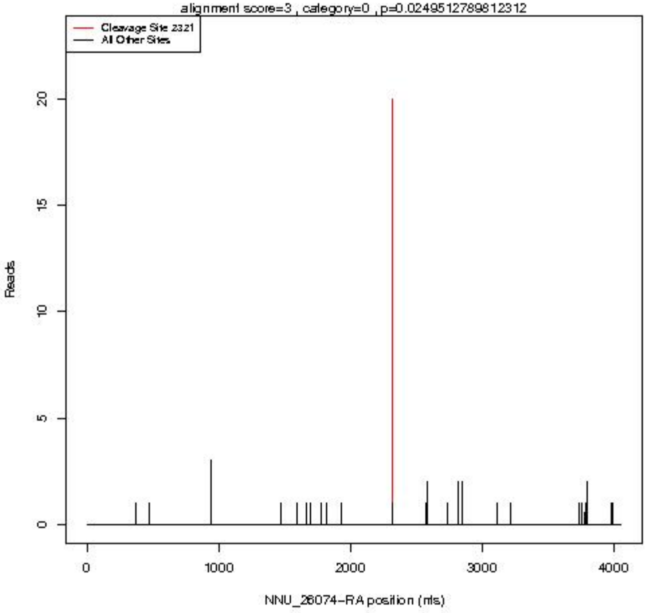

Nnu-miR169a slicing NNU_05128-RA at nt 1679

alignment score=2.5 , category=0 , p=0.00699426959672722

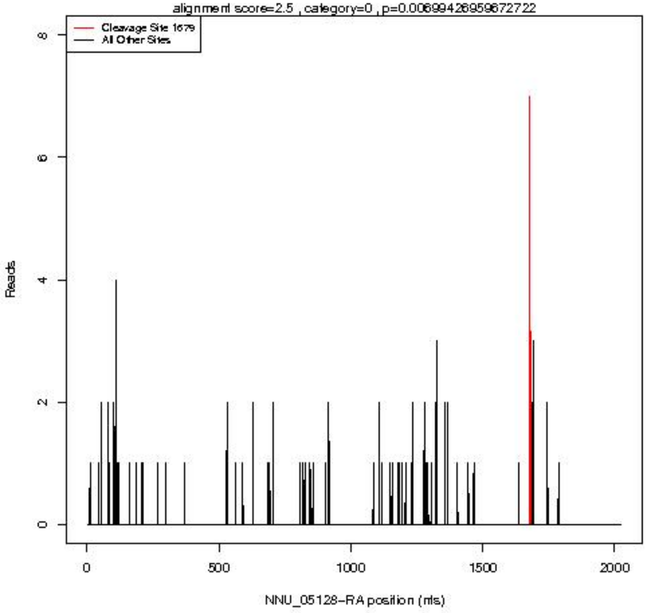

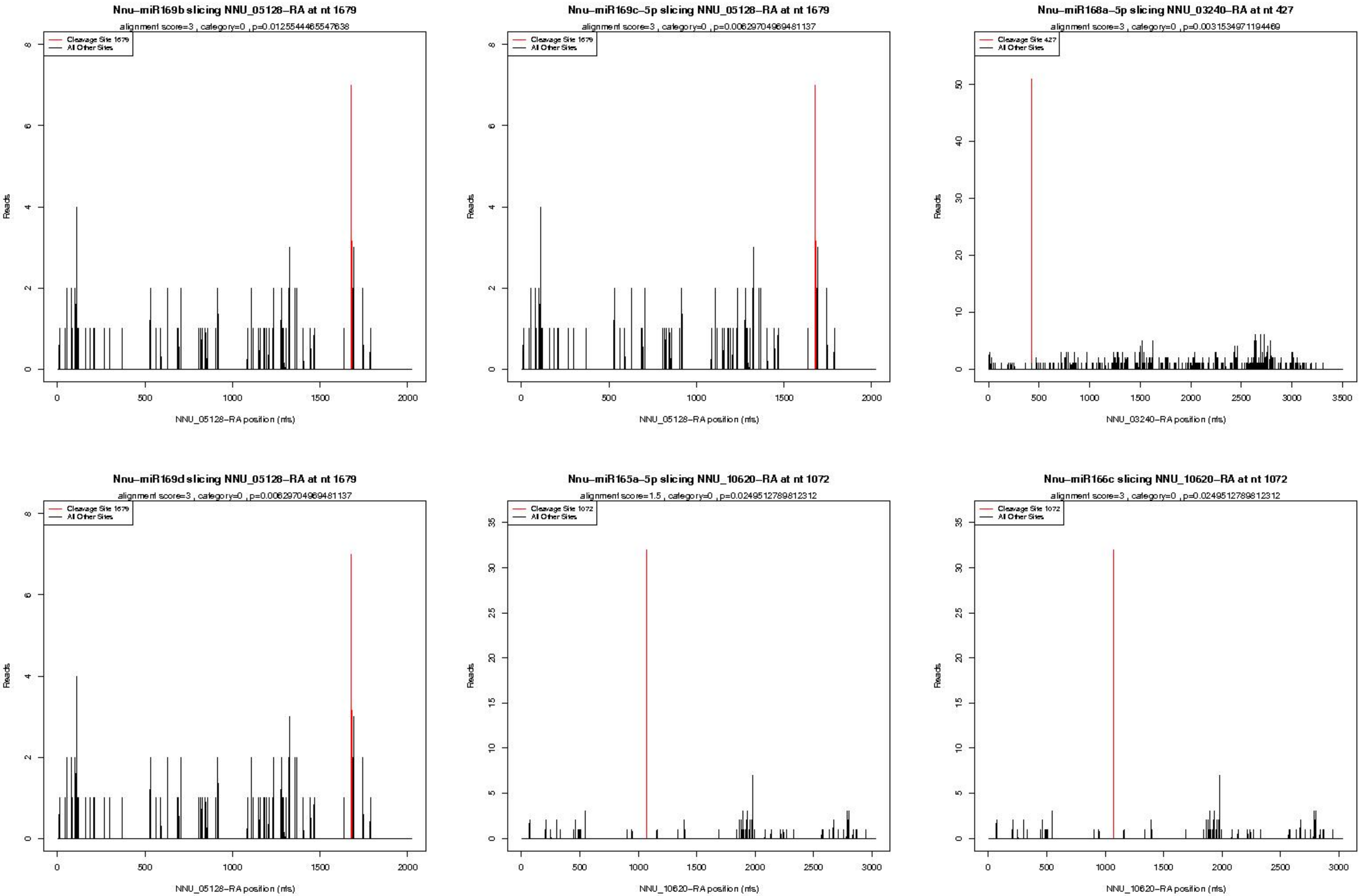
Nnu−miR169b slicing NNU_05128−RA at nt 1679
alignment score=3 , category=0 , p=0.0125544465547638
Cleavage Site 1679
All Other Sites
Reads
NNU_05128−RA position (nts)
Nnu−miR169c−5p slicing NNU_05128−RA at nt 1679
alignment score=3 , category=0 , p=0.00629704969481137
Cleavage Site 1679
All Other Sites
Reads
NNU_05128−RA position (nts)
Nnu−miR168a−5p slicing NNU_03240−RA at nt 427
alignment score=3 , category=0 , p=0.0031534971194469
Cleavage Site 427
All Other Sites
Reads
NNU_03240−RA position (nts)
Nnu−miR169d slicing NNU_05128−RA at nt 1679
alignment score=3 , category=0 , p=0.00629704969481137
Cleavage Site 1679
All Other Sites
Reads
NNU_05128−RA position (nts)
Nnu−miR165a−5p slicing NNU_10620−RA at nt 1072
alignment score=1.5 , category=0 , p=0.0249512789812312
Cleavage Site 1072
All Other Sites
Reads
NNU_10620−RA position (nts)
Nnu−miR166c slicing NNU_10620−RA at nt 1072
alignment score=3 , category=0 , p=0.0249512789812312
Cleavage Site 1072
All Other Sites
Reads
NNU_10620−RA position (nts)

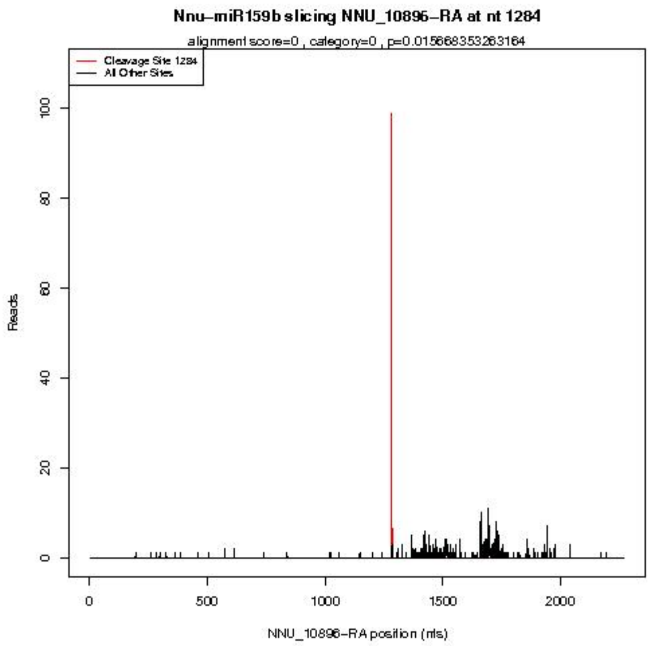
Nnu–miR159b slicing NNU_10896–RA at nt 1284
alignment score=0 , category=0 , p=0.015668353263164
Cleavage Site 1284
All Other Sites
Reads
NNU_10896–RA position (nts)

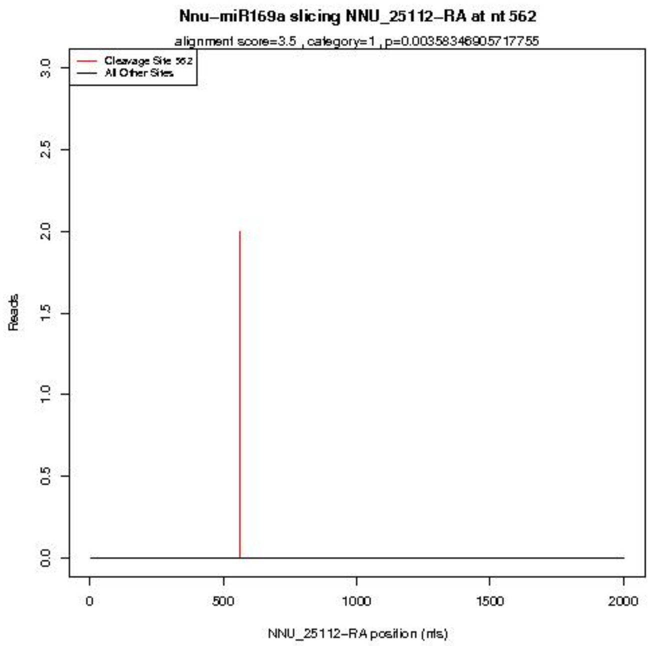
Nnu–miR169a slicing NNU_25112–RA at nt 562
alignment score=3.5 , category=1 , p=0.00358346905717755
Cleavage Site 562
All Other Sites
Reads
NNU_25112–RA position (nts)

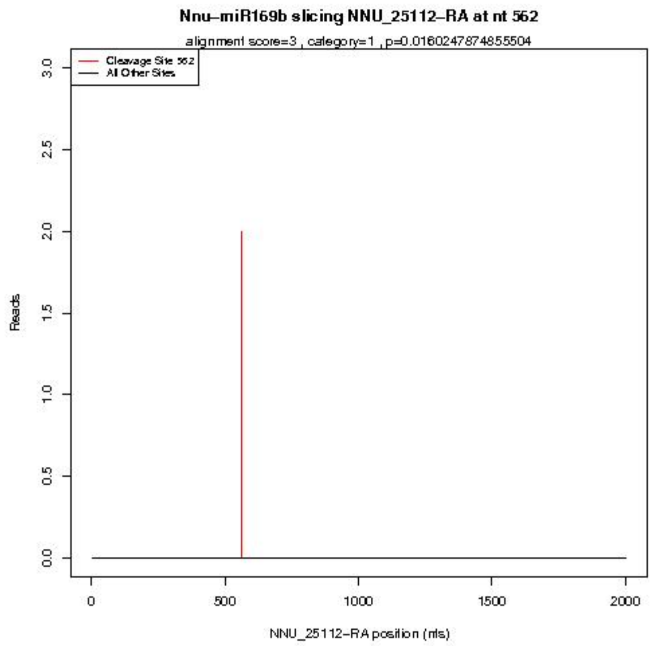
Nnu–miR169b slicing NNU_25112–RA at nt 562
alignment score=3 , category=1 , p=0.0160247874855504
Cleavage Site 562
All Other Sites
Reads
NNU_25112–RA position (nts)

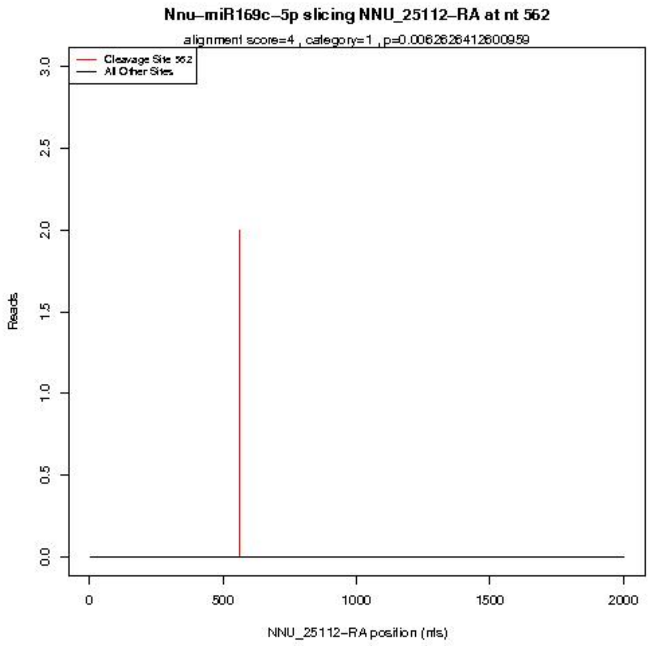
Nnu–miR169c–5p slicing NNU_25112–RA at nt 562
alignment score=4 , category=1 , p=0.0062626412600959
Cleavage Site 562
All Other Sites
Reads
NNU_25112–RA position (nts)

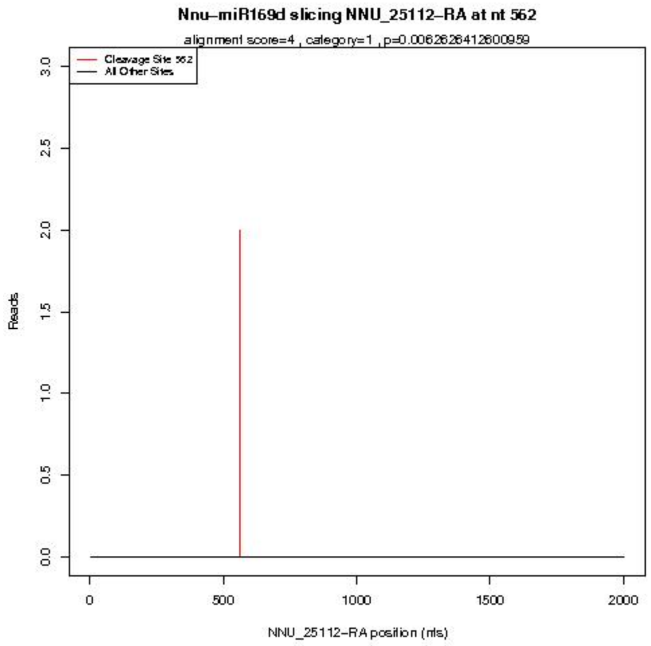
Nnu–miR169d slicing NNU_25112–RA at nt 562
alignment score=4 , category=1 , p=0.0062626412600959
Cleavage Site 562
All Other Sites
Reads
NNU_25112–RA position (nts)

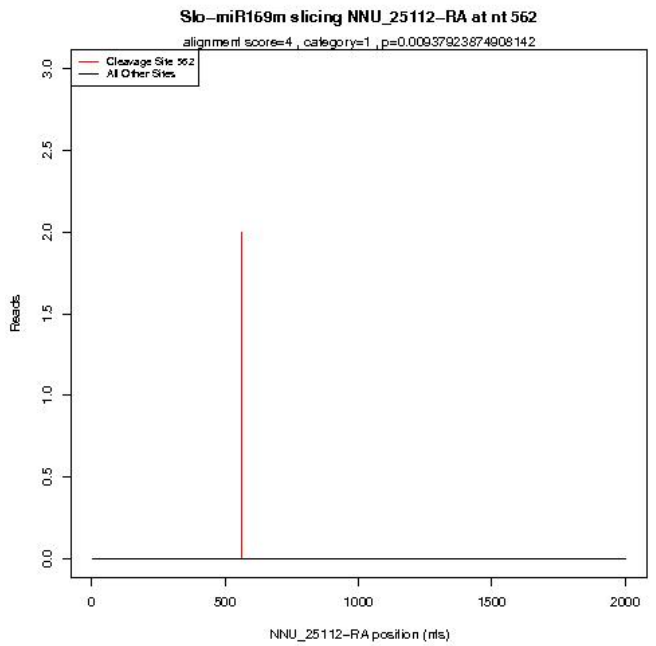
Slo–miR169m slicing NNU_25112–RA at nt 562
alignment score=4 , category=1 , p=0.00937923874908142
Cleavage Site 562
All Other Sites
Reads
NNU_25112–RA position (nts)

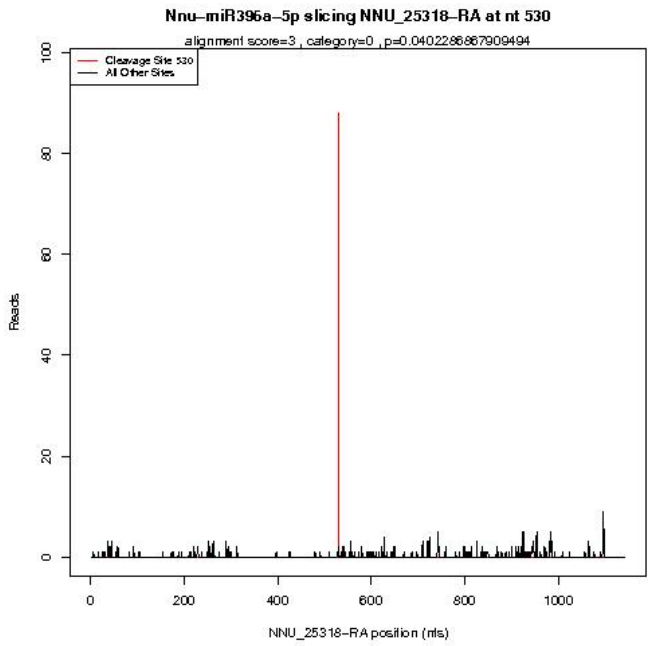
Nnu–miR396a–5p slicing NNU_25318–RA at nt 530
alignment score=3 , category=0 , p=0.0402286867909494
Cleavage Site 530
All Other Sites
Reads
NNU_25318–RA position (nts)

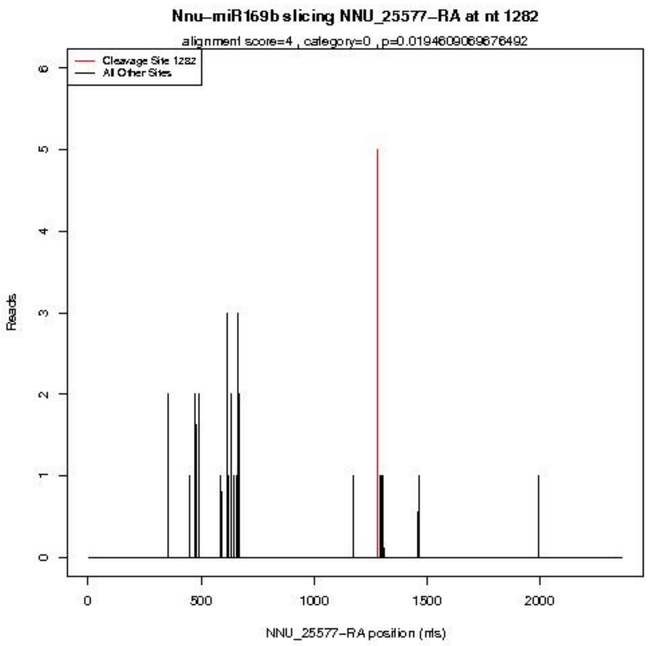
Nnu–miR169b slicing NNU_25577–RA at nt 1282
alignment score=4 , category=0 , p=0.0194609069676492
Cleavage Site 1282
All Other Sites
Reads
NNU_25577–RA position (nts)

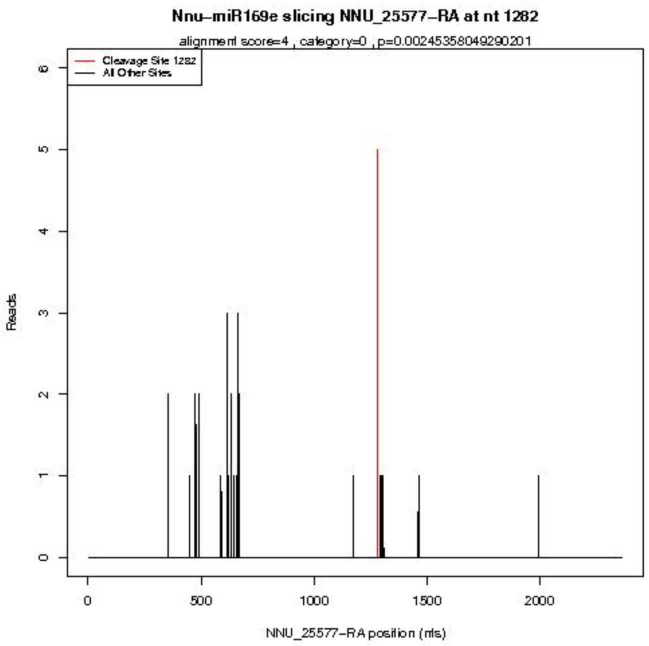
Nnu–miR169e slicing NNU_25577–RA at nt 1282
alignment score=4 , category=0 , p=0.00245358049290201
Cleavage Site 1282
All Other Sites
Reads
NNU_25577–RA position (nts)

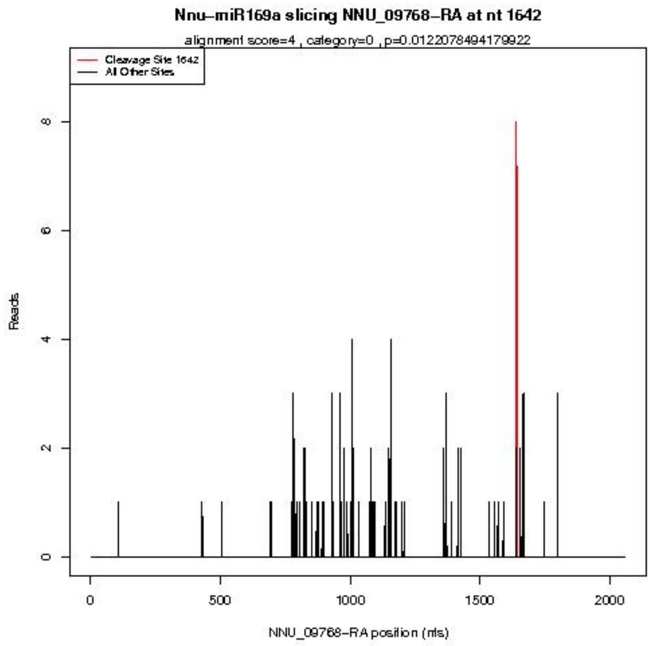
Nnu–miR169a slicing NNU_09768–RA at nt 1642
alignment score=4 , category=0 , p=0.0122078494179922
Cleavage Site 1642
All Other Sites
Reads
NNU_09768–RA position (nts)

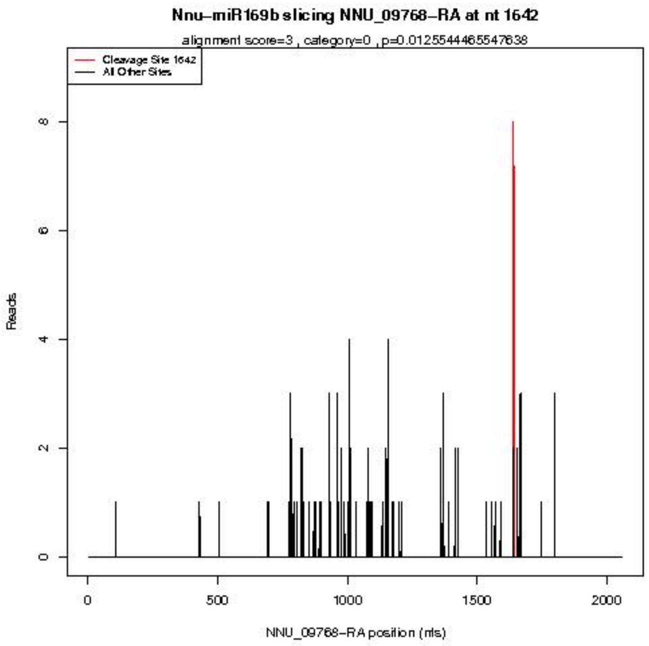
Nnu–miR169b slicing NNU_09768–RA at nt 1642
alignment score=3 , category=0 , p=0.0125544465547638
Cleavage Site 1642
All Other Sites
Reads
NNU_09768–RA position (nts)

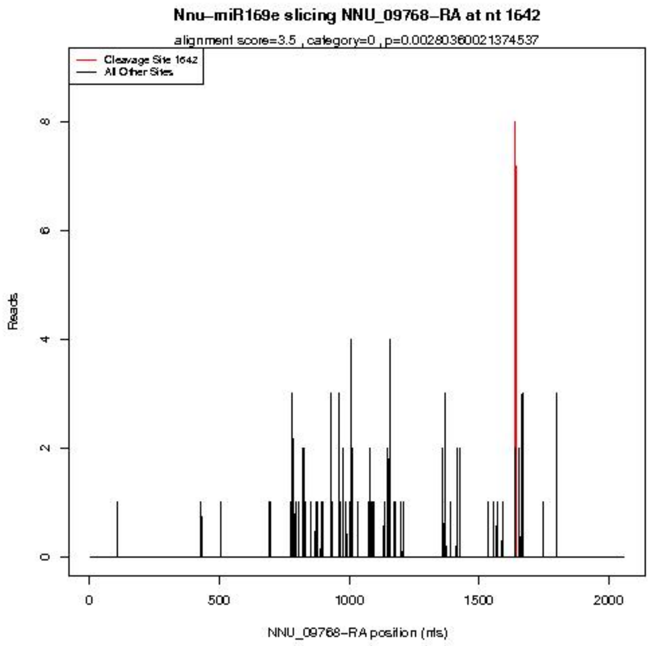
Nnu–miR169e slicing NNU_09768–RA at nt 1642
alignment score=3.5 , category=0 , p=0.00280360021374537
Cleavage Site 1642
All Other Sites
Reads
NNU_09768–RA position (nts)

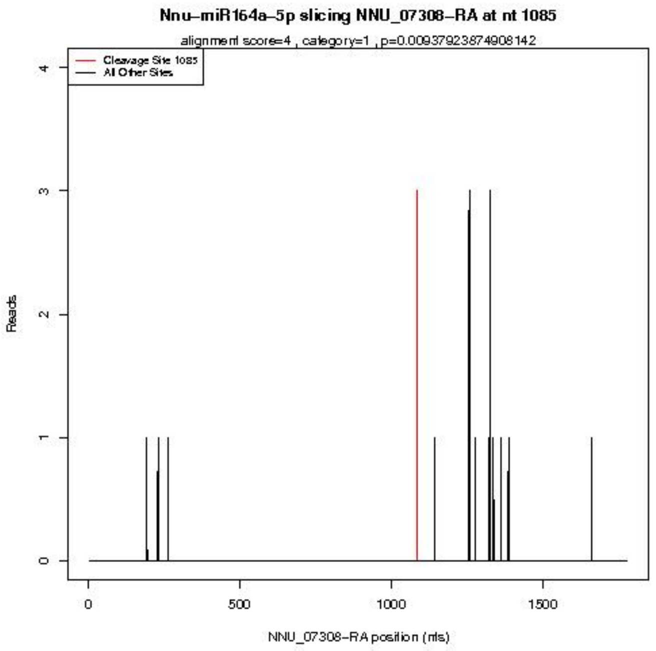
Nnu-miR164a-5p slicing NNU_07308-RA at nt 1085
alignment score=4 , category=1 , p=0.00937923874908142
Cleavage Site 1085
All Other Sites
Reads
NNU_07308-RA position (nts)

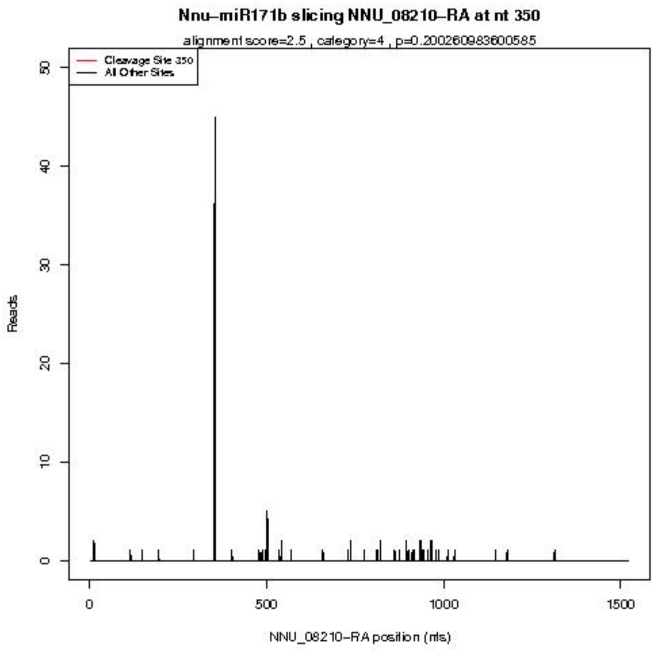
Nnu-miR171b slicing NNU_08210-RA at nt 350
alignment score=2.5 , category=4 , p=0.200260983600585
Cleavage Site 350
All Other Sites
Reads
NNU_08210-RA position (nts)

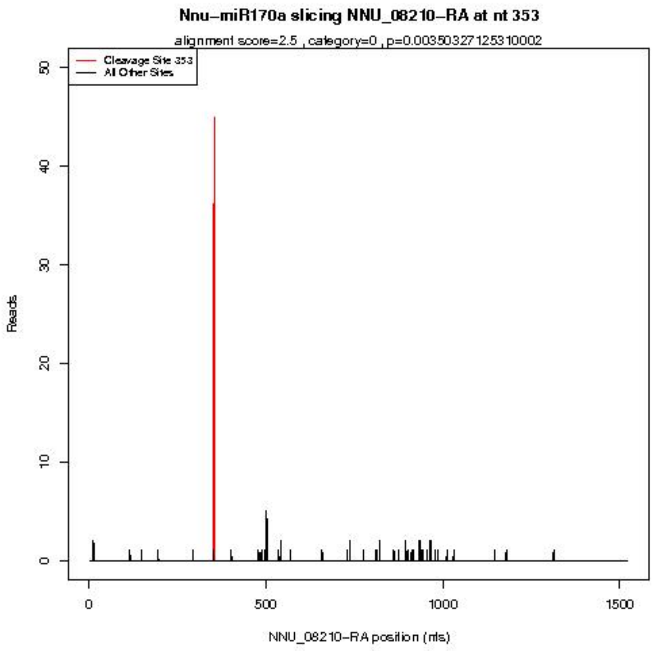
Nnu-miR170a slicing NNU_08210-RA at nt 353
alignment score=2.5 , category=0 , p=0.00350327125310002
Cleavage Site 353
All Other Sites
Reads
NNU_08210-RA position (nts)

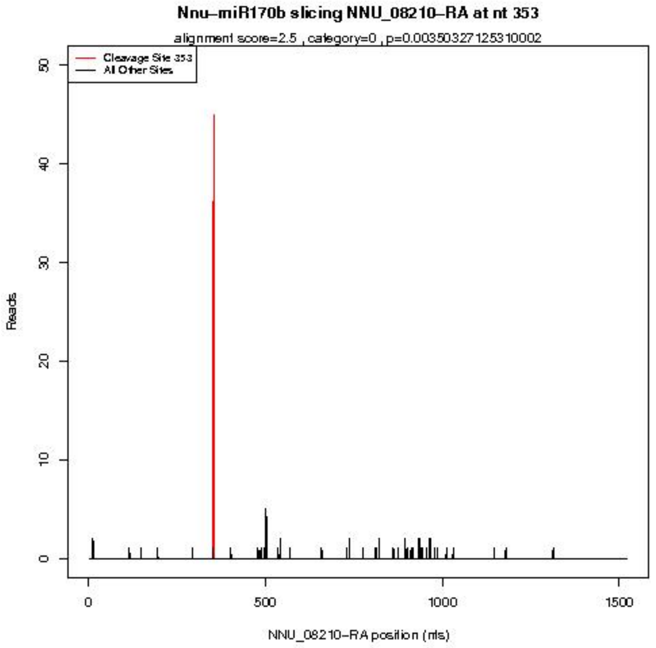
Nnu-miR170b slicing NNU_08210-RA at nt 353
alignment score=2.5 , category=0 , p=0.00350327125310002
Cleavage Site 353
All Other Sites
Reads
NNU_08210-RA position (nts)

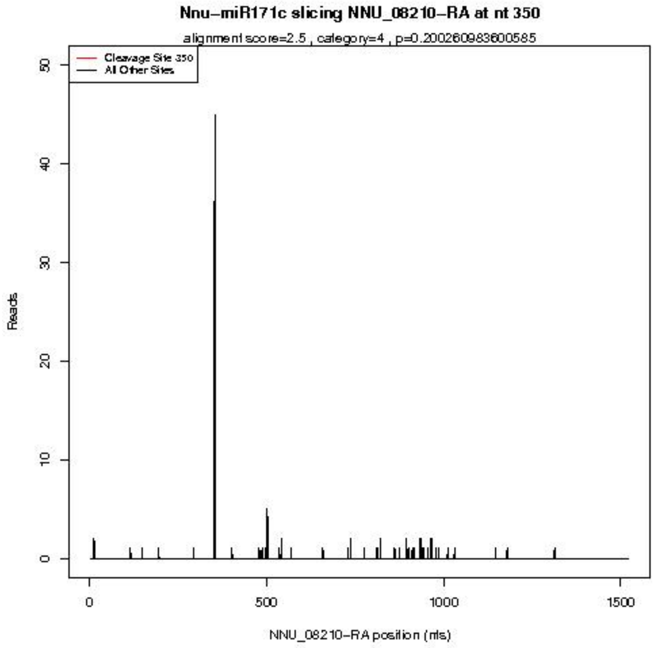
Nnu-miR171c slicing NNU_08210-RA at nt 350
alignment score=2.5 , category=4 , p=0.200260983600585
Cleavage Site 350
All Other Sites
Reads
NNU_08210-RA position (nts)

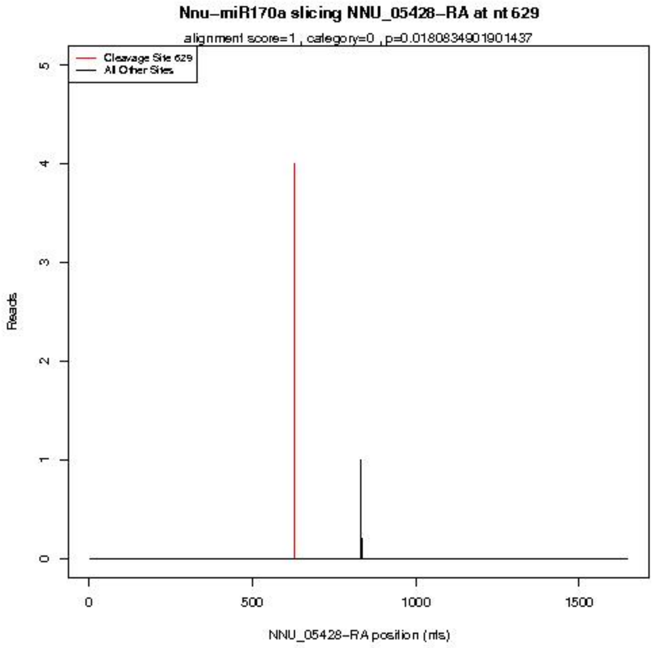
Nnu-miR170a slicing NNU_05428-RA at nt 629
alignment score=1 , category=0 , p=0.0180834901901437
Cleavage Site 629
All Other Sites
Reads
NNU_05428-RA position (nts)

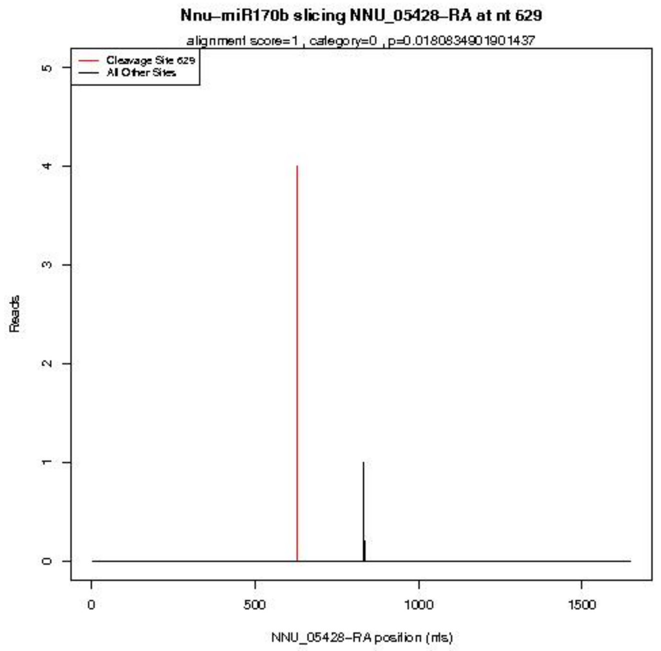
Nnu–miR170b slicing NNU_05428–RA at nt 629
alignment score=1 , category=0 , p=0.0180834901901437
Cleavage Site 629
All Other Sites
Reads
NNU_05428–RA position (nts)

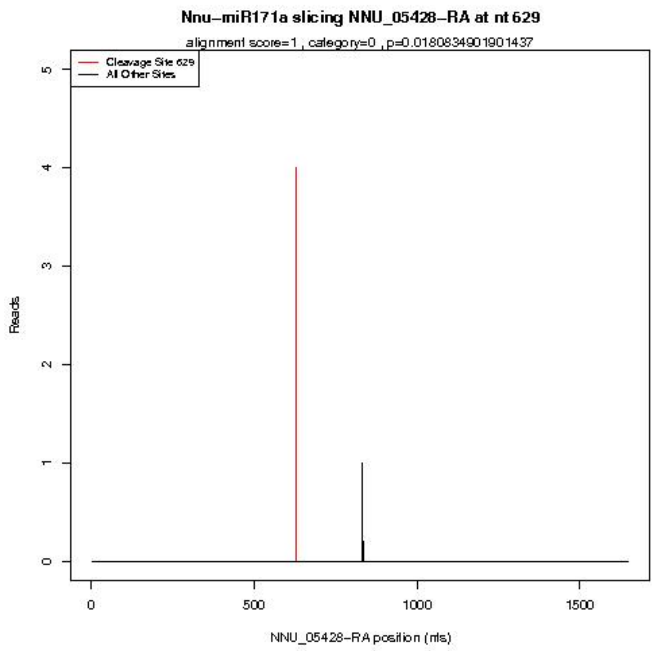
Nnu–miR171a slicing NNU_05428–RA at nt 629
alignment score=1 , category=0 , p=0.0180834901901437
Cleavage Site 629
All Other Sites
Reads
NNU_05428–RA position (nts)

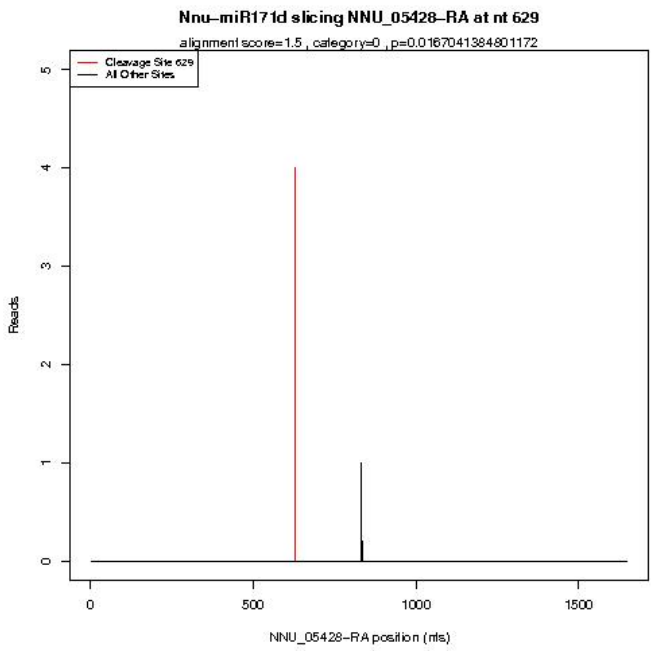
Nnu–miR171d slicing NNU_05428–RA at nt 629
alignment score=1.5 , category=0 , p=0.0167041384801172
Cleavage Site 629
All Other Sites
Reads
NNU_05428–RA position (nts)

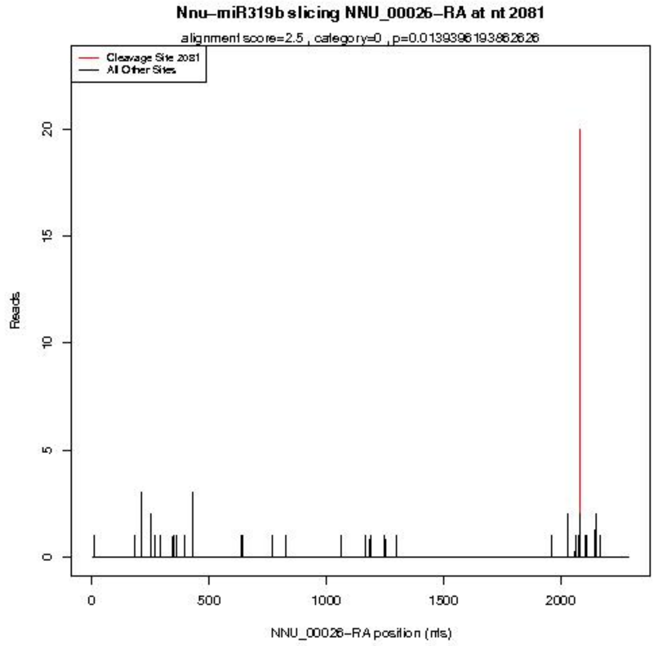
Nnu–miR319b slicing NNU_00026–RA at nt 2081
alignment score=2.5 , category=0 , p=0.0139396193862626
Cleavage Site 2081
All Other Sites
Reads
NNU_00026–RA position (nts)

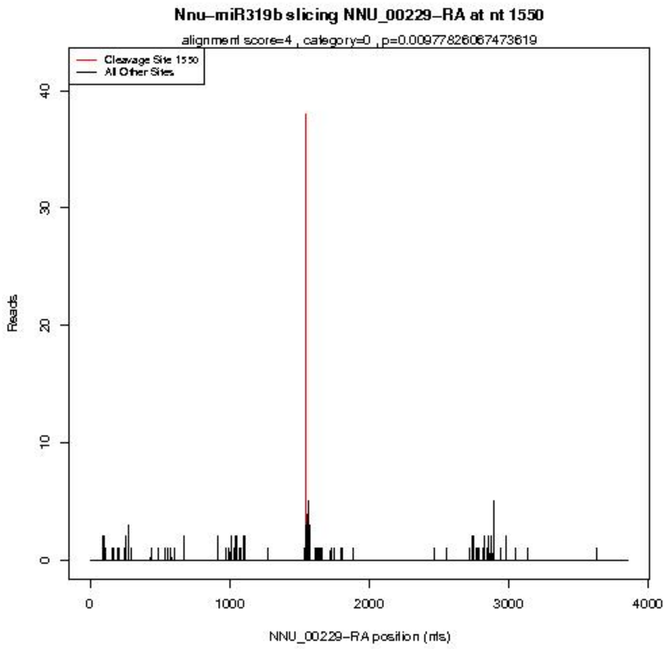
Nnu–miR319b slicing NNU_00229–RA at nt 1550
alignment score=4 , category=0 , p=0.00977826067473619
Cleavage Site 1550
All Other Sites
Reads
NNU_00229–RA position (nts)

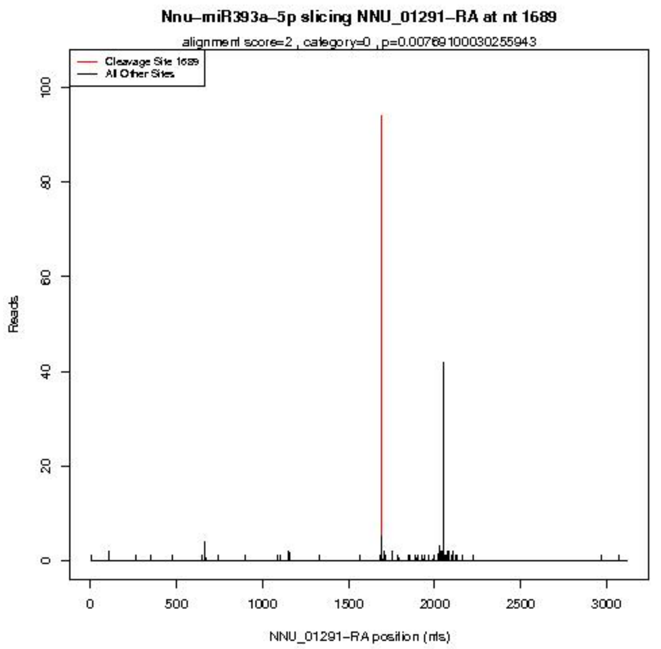
Nnu–miR393a–5p slicing NNU_01291–RA at nt 1689
alignment score=2 , category=0 , p=0.00769100030255943
Cleavage Site 1689
All Other Sites
Reads
NNU_01291–RA position (nts)

Nnu–miR393b–5p slicing NNU_01291–RA at nt 1689

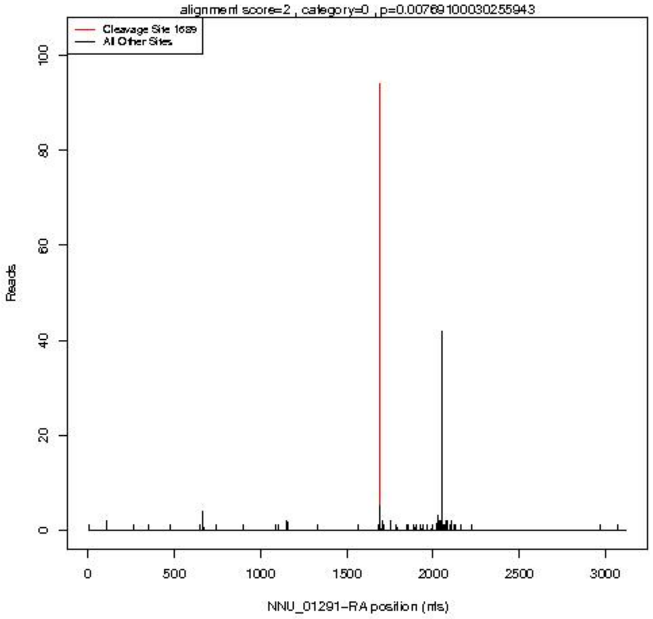

Nnu–miR393c slicing NNU_01291–RA at nt 1689

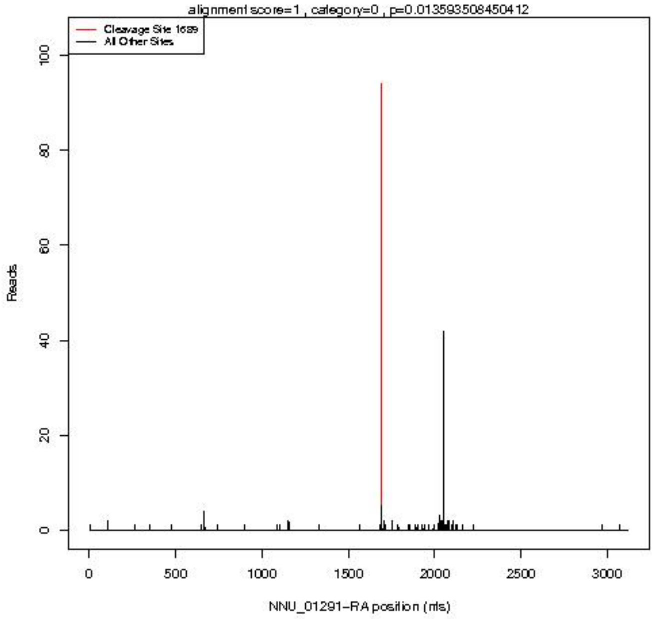

Nnu–miR393e slicing NNU_01291–RA at nt 1689

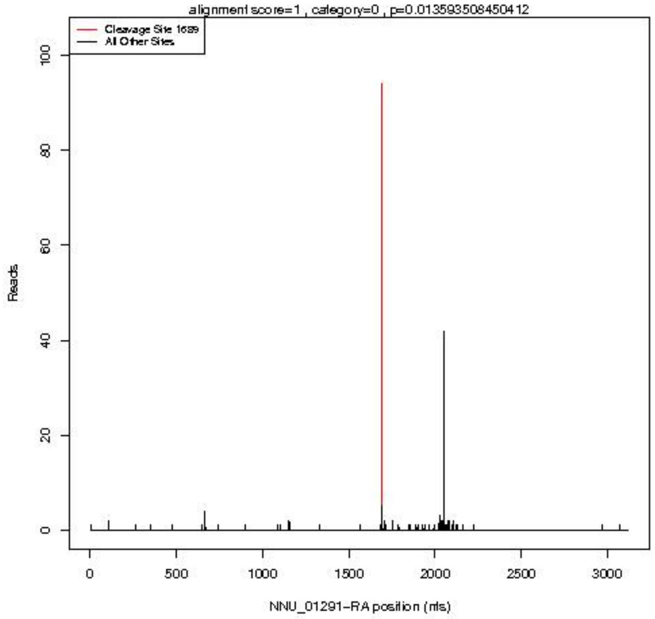

Nnu–miR319b slicing NNU_05145–RA at nt 1587

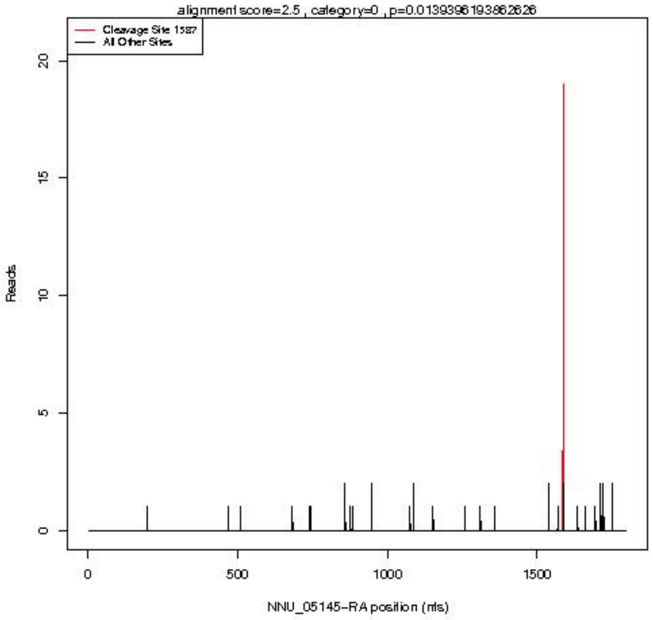

Nnu–miR390a–5p slicing NNU_06105–RA at nt 655

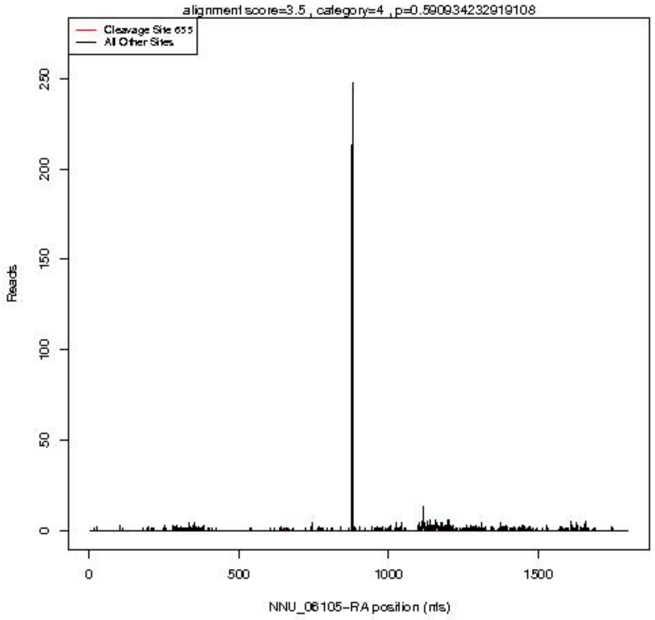

Nnu–miR393a–5p slicing NNU_06904–RA at nt 1987

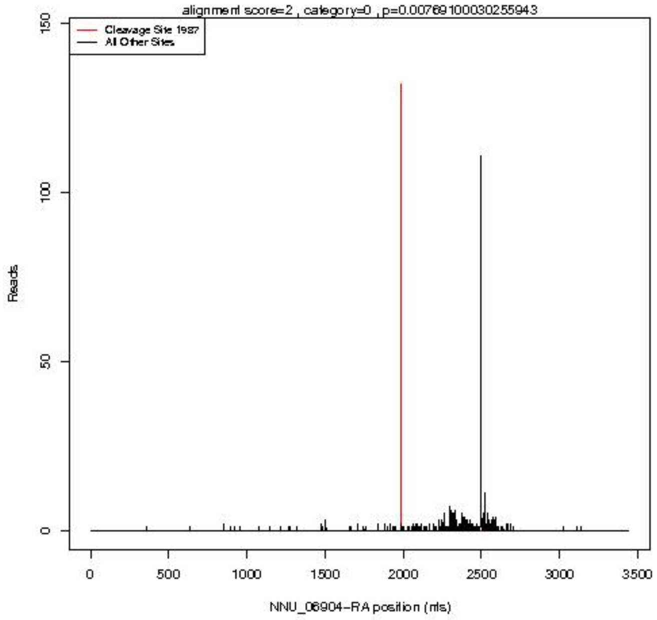

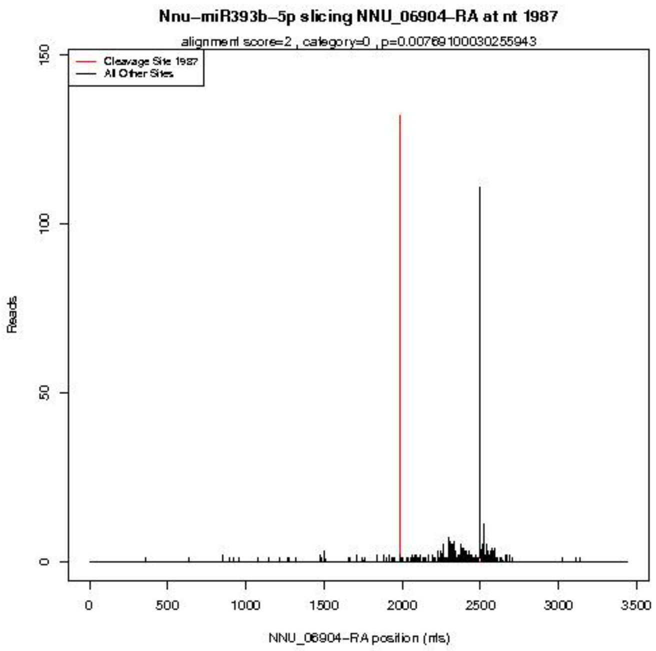
Nnu–miR393b–5p slicing NNU_06904–RA at nt 1987
alignment score=2 , category=0 , p=0.00769100030255943
Cleavage Site 1987
All Other Sites
Reads
NNU_06904–RA position (nts)

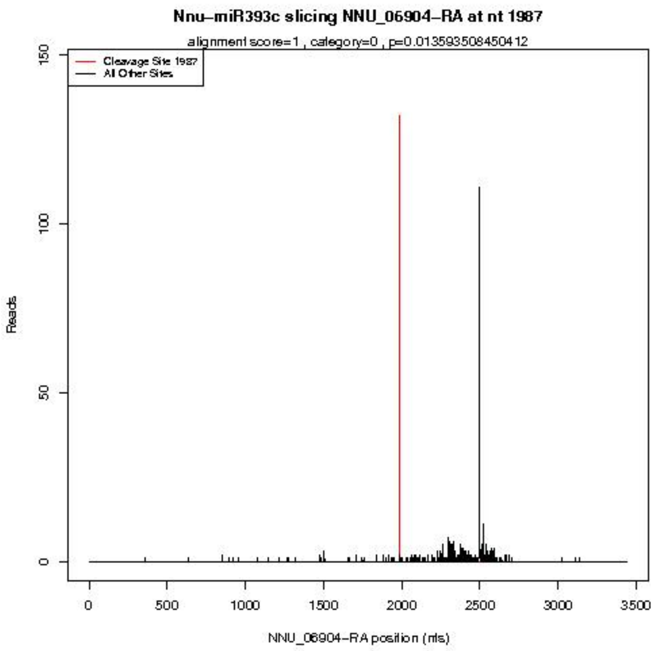
Nnu–miR393c slicing NNU_06904–RA at nt 1987
alignment score=1 , category=0 , p=0.013593508450412
Cleavage Site 1987
All Other Sites
Reads
NNU_06904–RA position (nts)

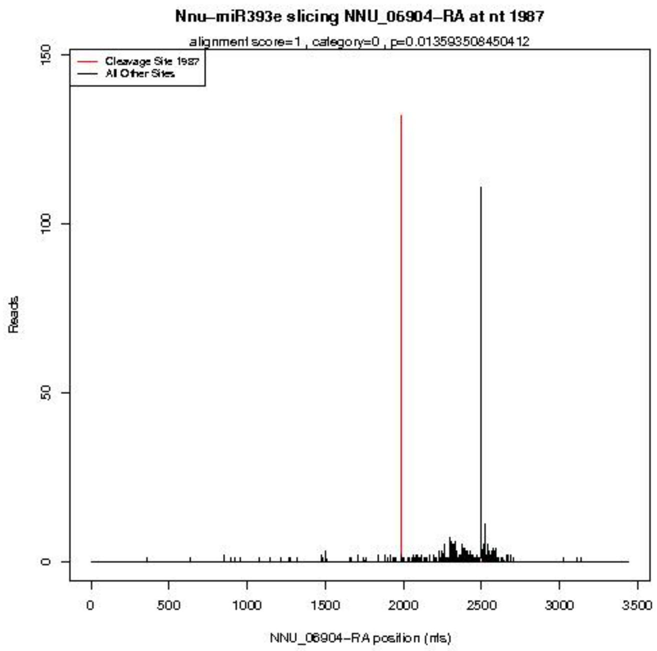
Nnu–miR393e slicing NNU_06904–RA at nt 1987
alignment score=1 , category=0 , p=0.013593508450412
Cleavage Site 1987
All Other Sites
Reads
NNU_06904–RA position (nts)

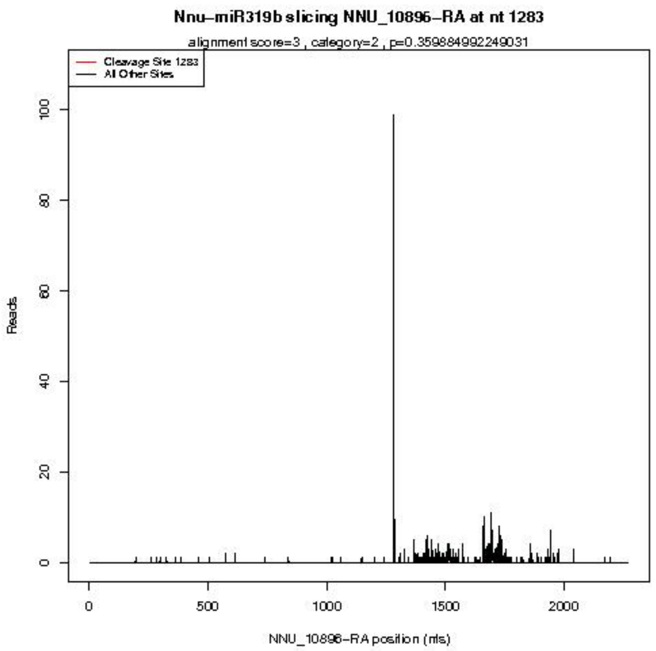
Nnu–miR319b slicing NNU_10896–RA at nt 1283
alignment score=3 , category=2 , p=0.359884992249031
Cleavage Site 1283
All Other Sites
Reads
NNU_10896–RA position (nts)

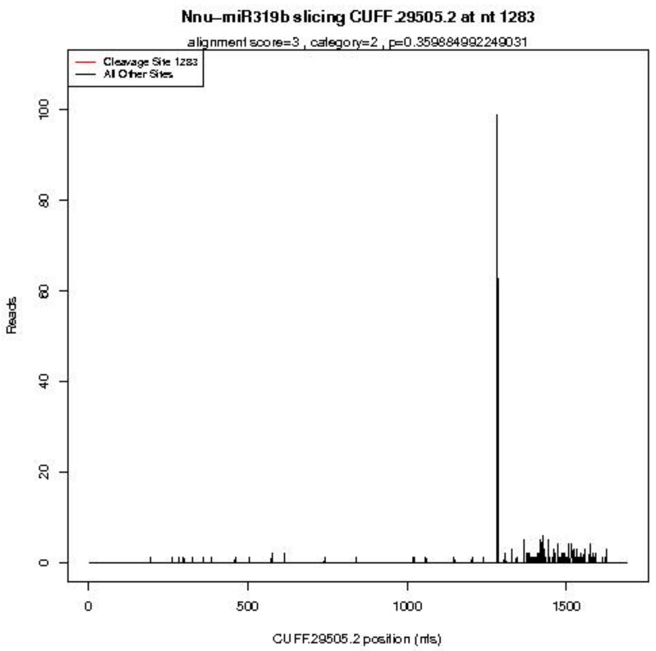
Nnu–miR319b slicing CUFF.29505.2 at nt 1283
alignment score=3 , category=2 , p=0.359884992249031
Cleavage Site 1283
All Other Sites
Reads
CUFF.29505.2 position (nts)

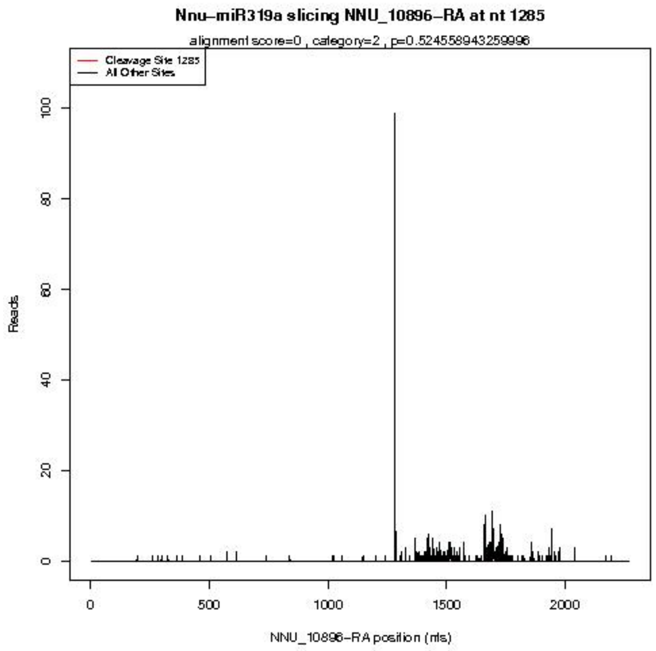
Nnu–miR319a slicing NNU_10896–RA at nt 1285
alignment score=0 , category=2 , p=0.524558943259996
Cleavage Site 1285
All Other Sites
Reads
NNU_10896–RA position (nts)

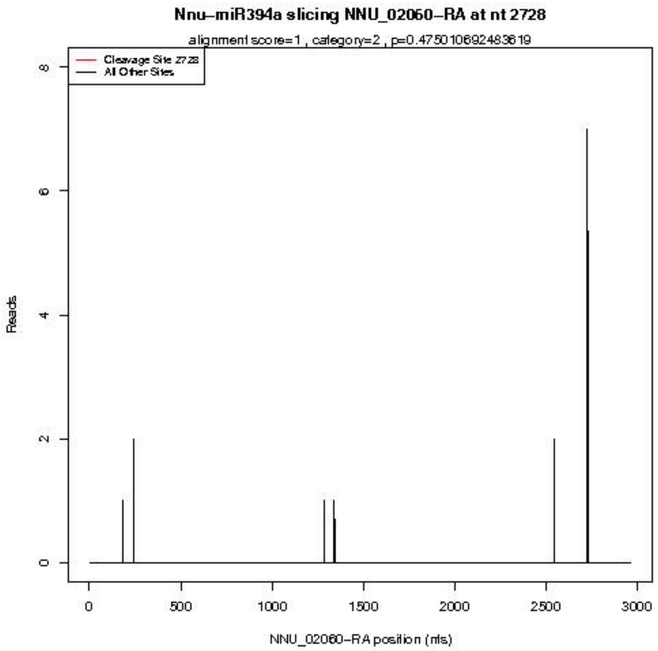
Nnu–miR394a slicing NNU_02060–RA at nt 2728
alignment score=1 , category=2 , p=0.475010692483619
Cleavage Site 2728
All Other Sites
Reads
NNU_02060–RA position (nts)

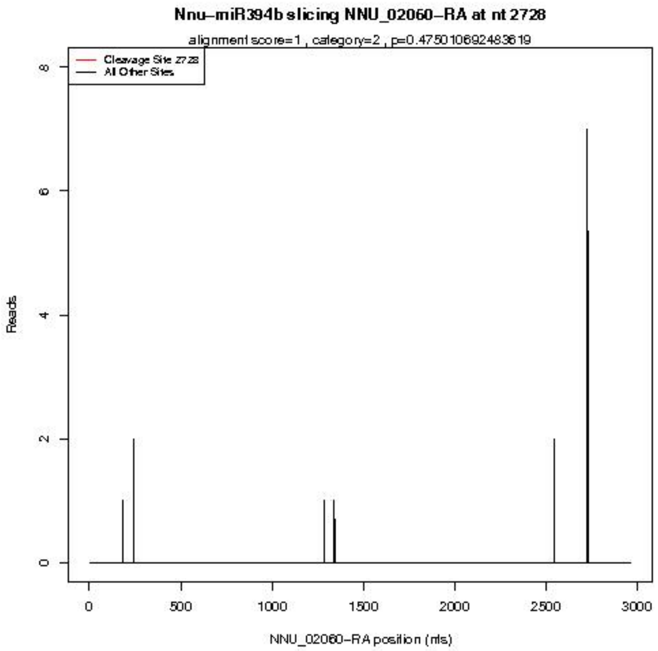
Nnu–miR394b slicing NNU_02060–RA at nt 2728
alignment score=1 , category=2 , p=0.475010692483619
Cleavage Site 2728
All Other Sites
Reads
NNU_02060–RA position (nts)

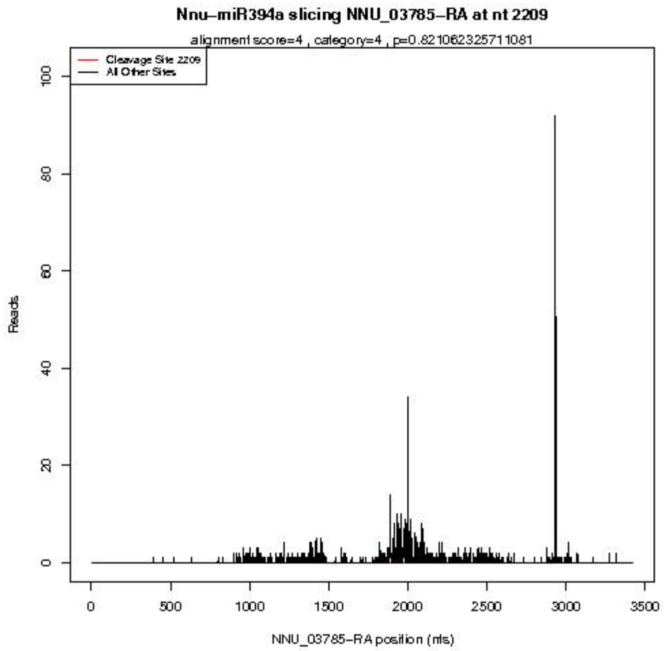
Nnu–miR394a slicing NNU_03785–RA at nt 2209
alignment score=4 , category=4 , p=0.821062325711081
Cleavage Site 2209
All Other Sites
Reads
NNU_03785–RA position (nts)

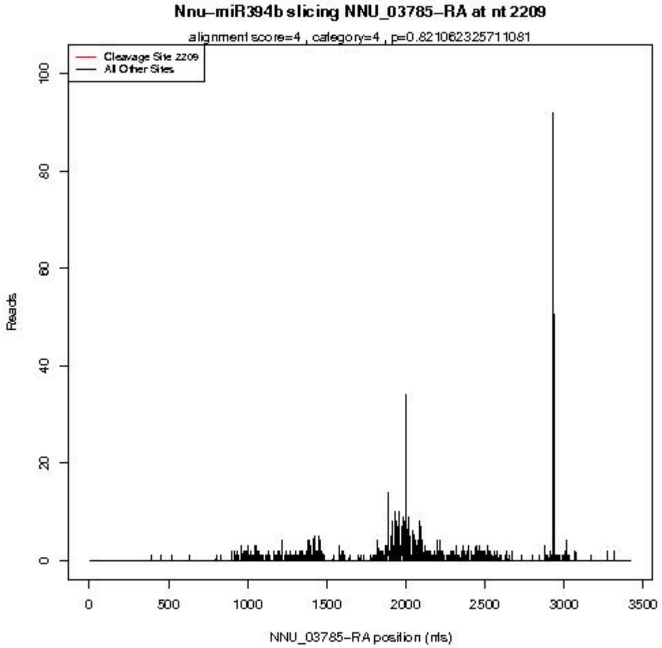
Nnu–miR394b slicing NNU_03785–RA at nt 2209
alignment score=4 , category=4 , p=0.821062325711081
Cleavage Site 2209
All Other Sites
Reads
NNU_03785–RA position (nts)

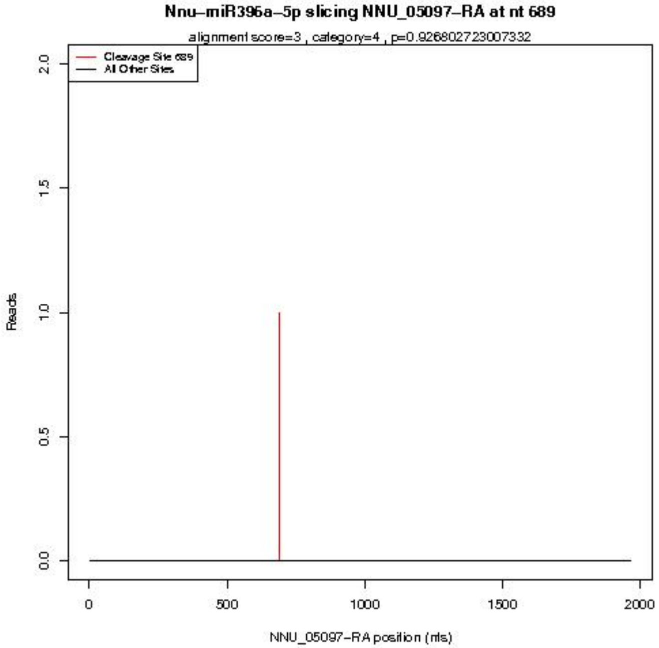
Nnu–miR396a–5p slicing NNU_05097–RA at nt 689
alignment score=3 , category=4 , p=0.926802723007332
Cleavage Site 689
All Other Sites
Reads
NNU_05097–RA position (nts)

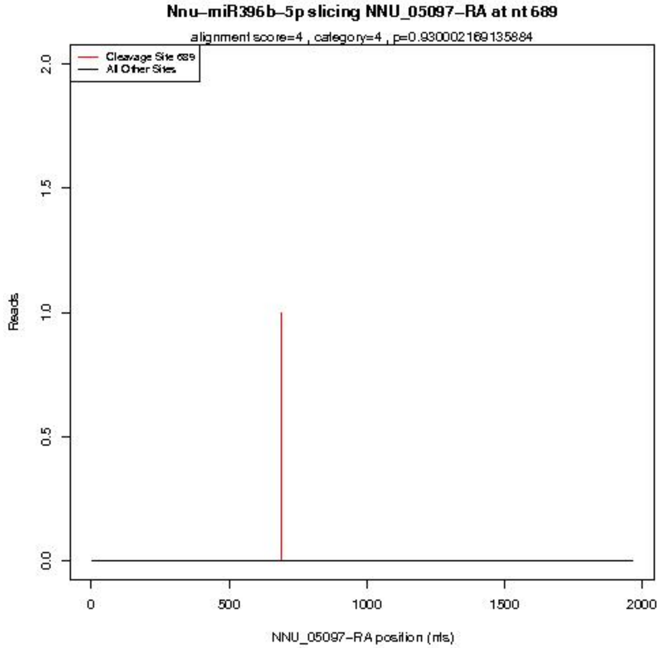
Nnu–miR396b–5p slicing NNU_05097–RA at nt 689
alignment score=4 , category=4 , p=0.930002169135884
Cleavage Site 689
All Other Sites
Reads
NNU_05097–RA position (nts)

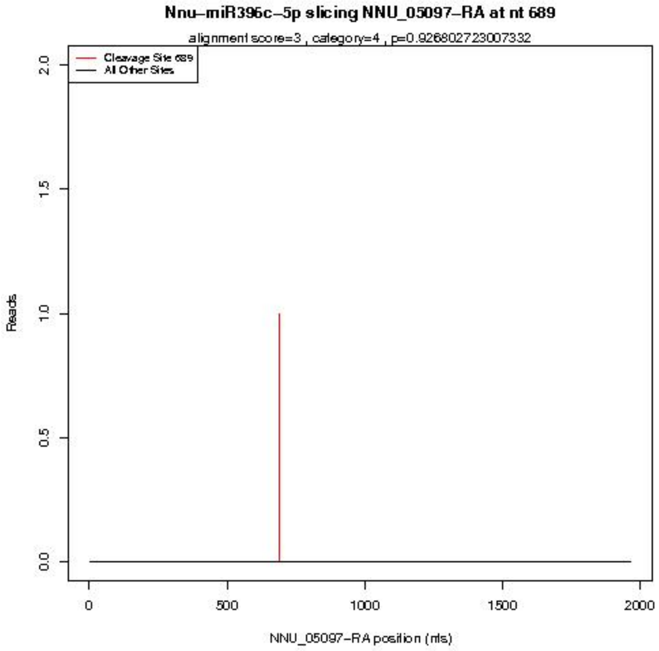
Nnu−miR396c−5p slicing NNU_05097−RA at nt 689
alignment score=3 , category=4 , p=0.926802723007332
Cleavage Site 689
All Other Sites
Reads
NNU_05097−RA position (nts)

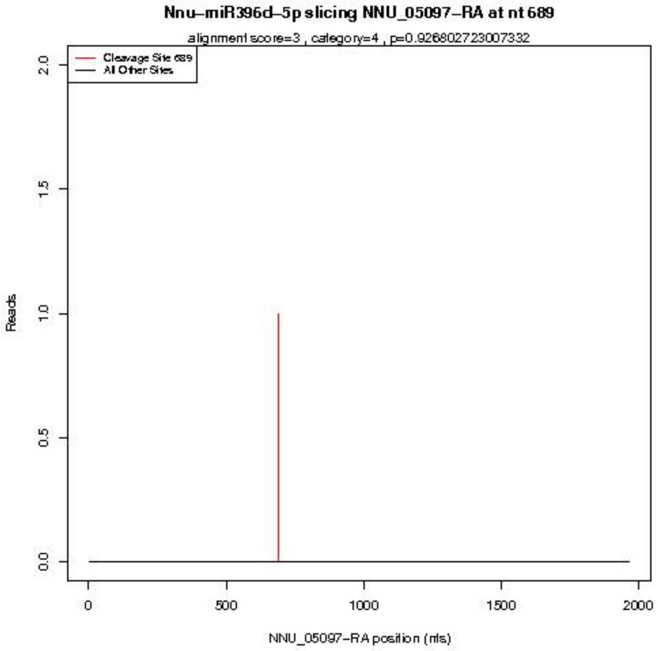
Nnu−miR396d−5p slicing NNU_05097−RA at nt 689
alignment score=3 , category=4 , p=0.926802723007332
Cleavage Site 689
All Other Sites
Reads
NNU_05097−RA position (nts)

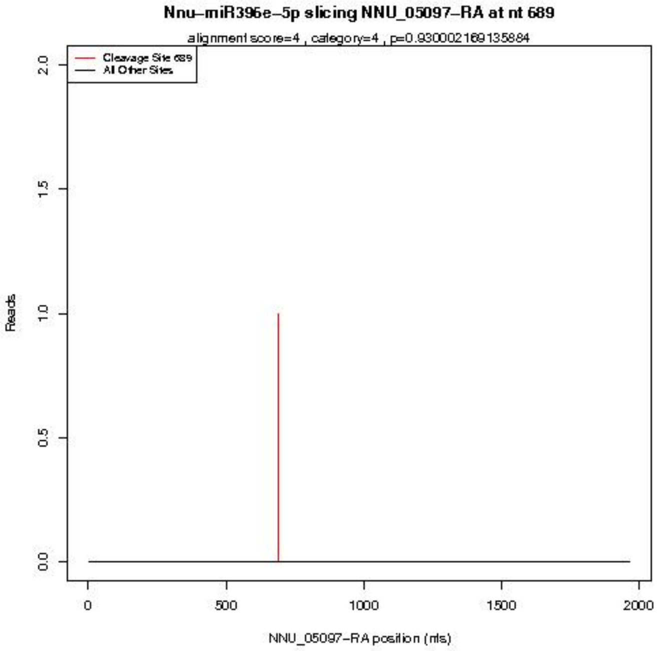
Nnu−miR396e−5p slicing NNU_05097−RA at nt 689
alignment score=4 , category=4 , p=0.930002169135884
Cleavage Site 689
All Other Sites
Reads
NNU_05097−RA position (nts)

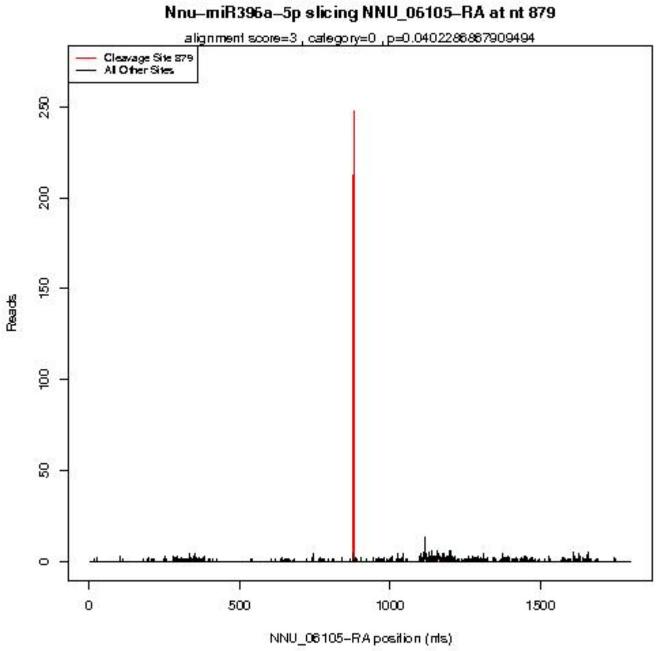
Nnu−miR396a−5p slicing NNU_06105−RA at nt 879
alignment score=3 , category=0 , p=0.0402286867909494
Cleavage Site 879
All Other Sites
Reads
NNU_06105−RA position (nts)

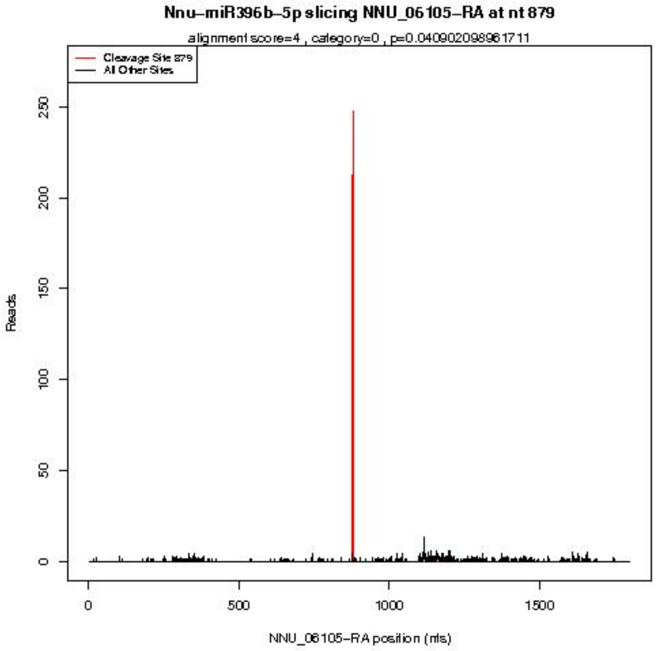
Nnu−miR396b−5p slicing NNU_06105−RA at nt 879
alignment score=4 , category=0 , p=0.040902098961711
Cleavage Site 879
All Other Sites
Reads
NNU_06105−RA position (nts)

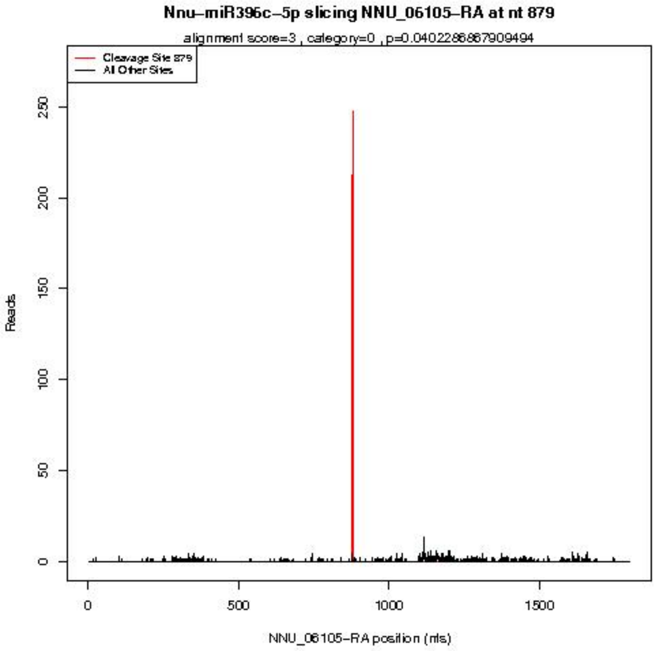
Nnu−miR396c−5p slicing NNU_06105−RA at nt 879
alignment score=3 , category=0 , p=0.0402286867909494
Cleavage Site 879
All Other Sites
Reads
NNU_06105−RA position (nts)

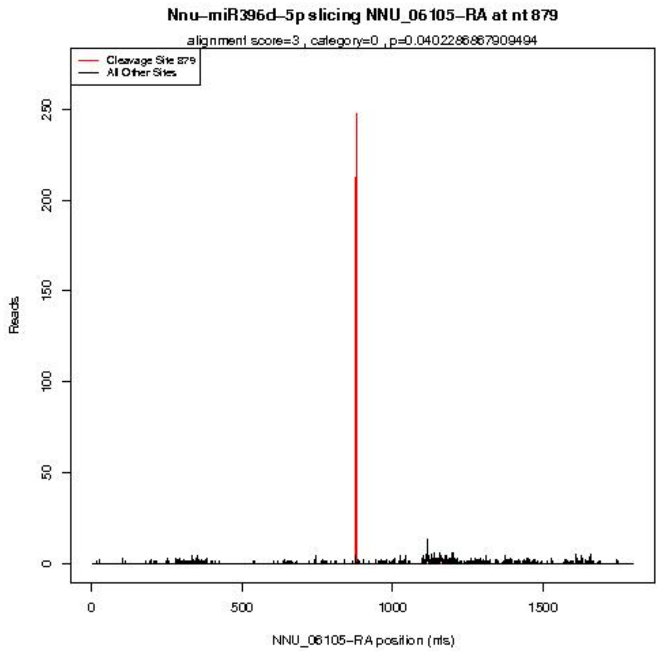
Nnu–miR396d–5p slicing NNU_06105–RA at nt 879
alignment score=3 , category=0 , p=0.0402286867909494
Cleavage Site 879
All Other Sites
Reads
NNU_06105–RA position (nts)

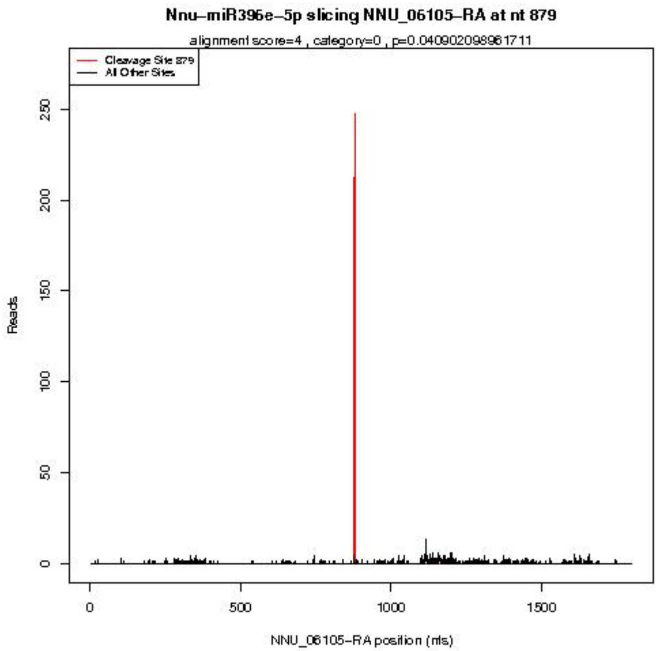
Nnu–miR396e–5p slicing NNU_06105–RA at nt 879
alignment score=4 , category=0 , p=0.040902098961711
Cleavage Site 879
All Other Sites
Reads
NNU_06105–RA position (nts)

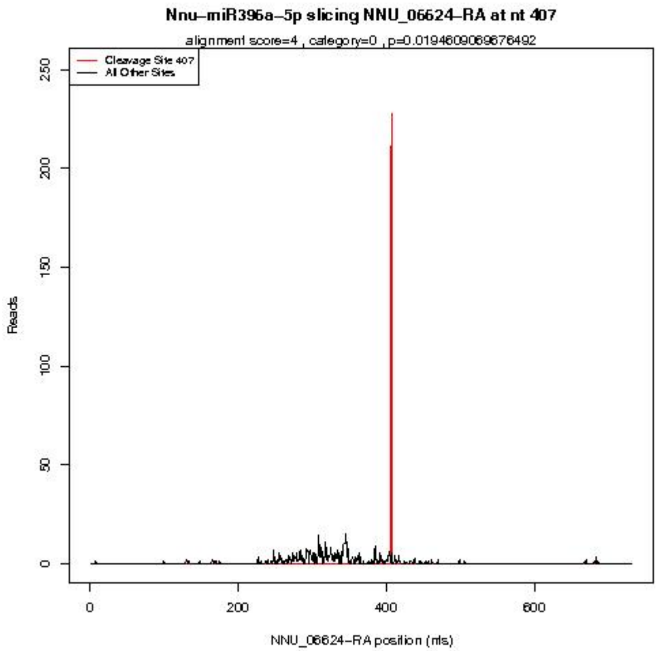
Nnu–miR396a–5p slicing NNU_06624–RA at nt 407
alignment score=4 , category=0 , p=0.0194609069676492
Cleavage Site 407
All Other Sites
Reads
NNU_06624–RA position (nts)

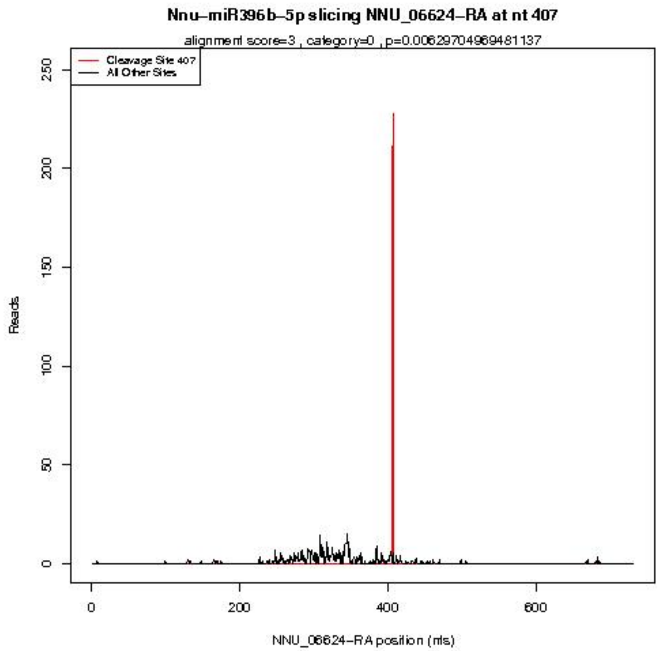
Nnu–miR396b–5p slicing NNU_06624–RA at nt 407
alignment score=3 , category=0 , p=0.00629704969481137
Cleavage Site 407
All Other Sites
Reads
NNU_06624–RA position (nts)

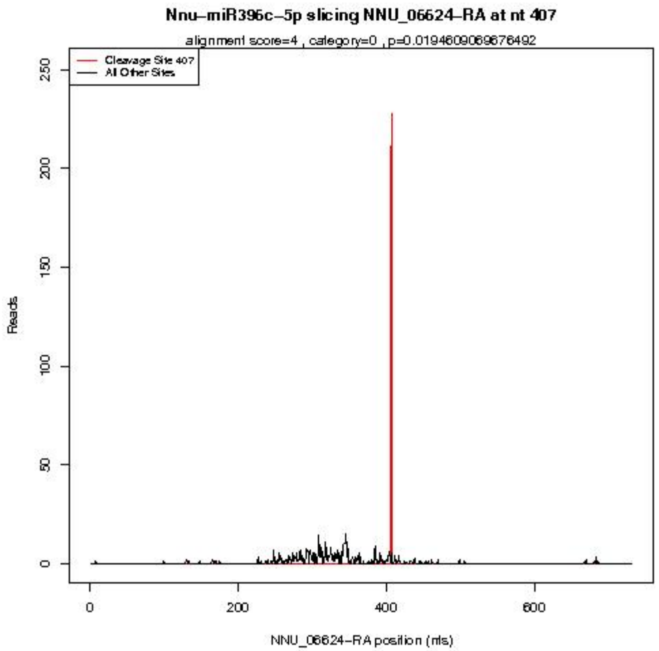
Nnu–miR396c–5p slicing NNU_06624–RA at nt 407
alignment score=4 , category=0 , p=0.0194609069676492
Cleavage Site 407
All Other Sites
Reads
NNU_06624–RA position (nts)

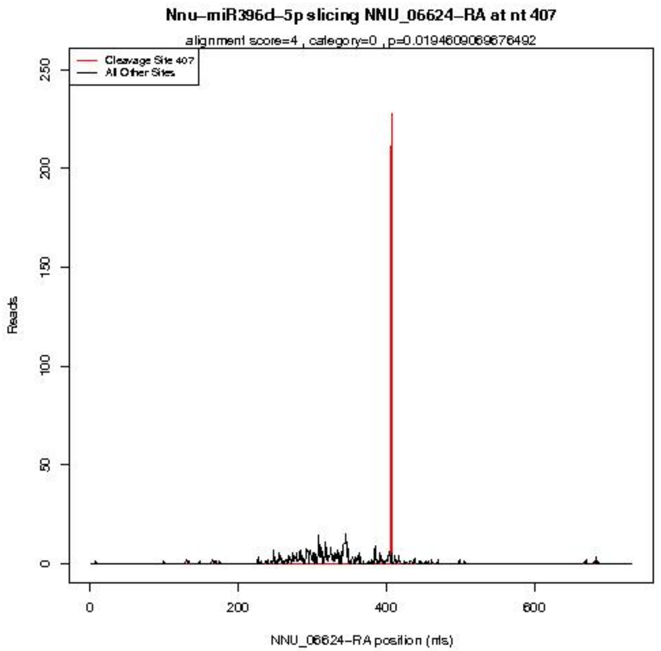
Nnu–miR396d–5p slicing NNU_06624–RA at nt 407
alignment score=4 , category=0 , p=0.0194609069676492
Cleavage Site 407
All Other Sites
Reads
NNU_06624–RA position (nts)

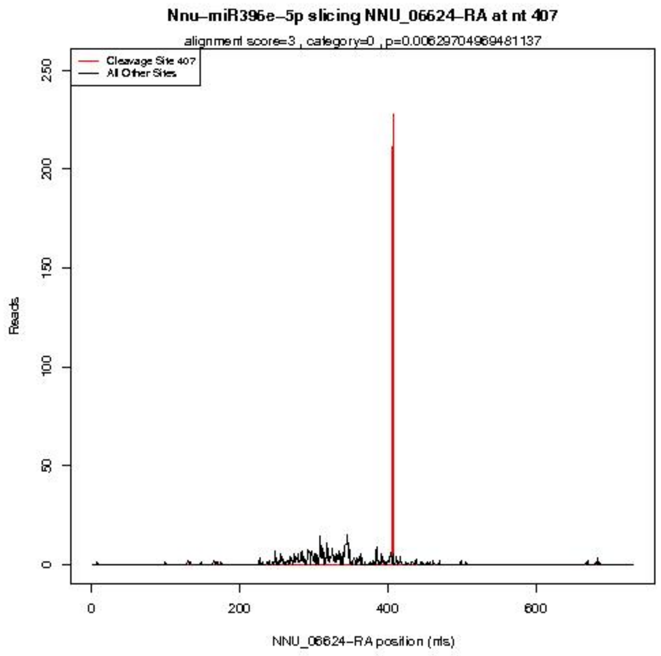
Nnu–miR396e–5p slicing NNU_06624–RA at nt 407
alignment score=3 , category=0 , p=0.00629704969481137
Cleavage Site 407
All Other Sites
Reads
NNU_06624–RA position (nts)

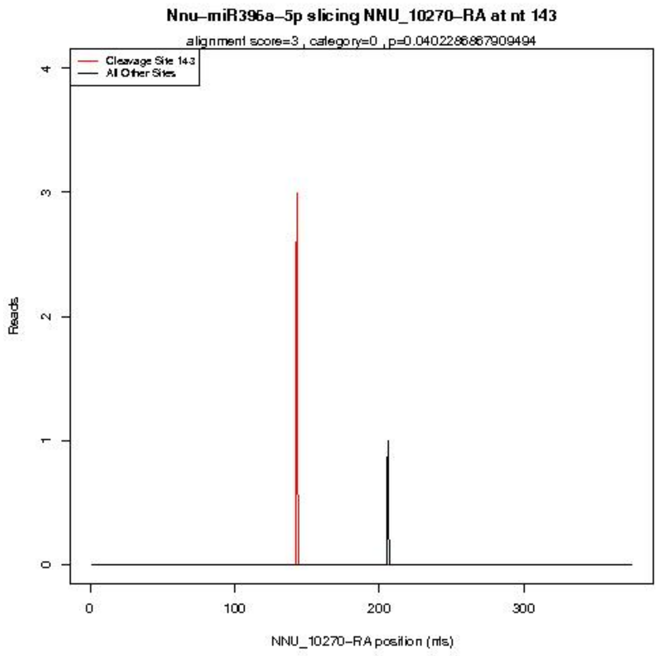
Nnu–miR396a–5p slicing NNU_10270–RA at nt 143
alignment score=3 , category=0 , p=0.0402286867909494
Cleavage Site 143
All Other Sites
Reads
NNU_10270–RA position (nts)

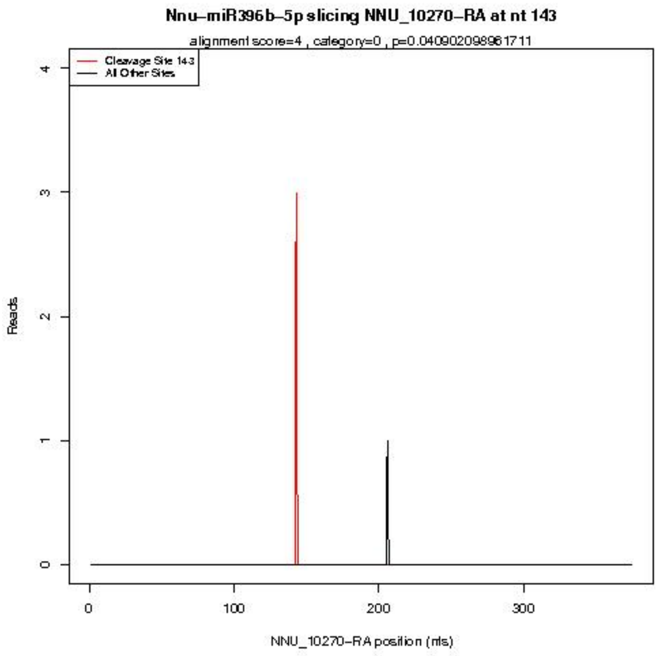
Nnu–miR396b–5p slicing NNU_10270–RA at nt 143
alignment score=4 , category=0 , p=0.040902098961711
Cleavage Site 143
All Other Sites
Reads
NNU_10270–RA position (nts)

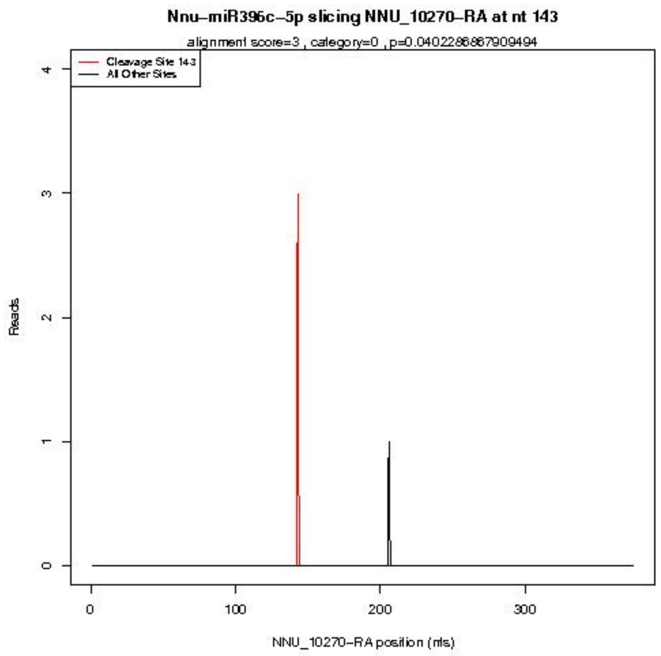
Nnu–miR396c–5p slicing NNU_10270–RA at nt 143
alignment score=3 , category=0 , p=0.0402286867909494
Cleavage Site 143
All Other Sites
Reads
NNU_10270–RA position (nts)

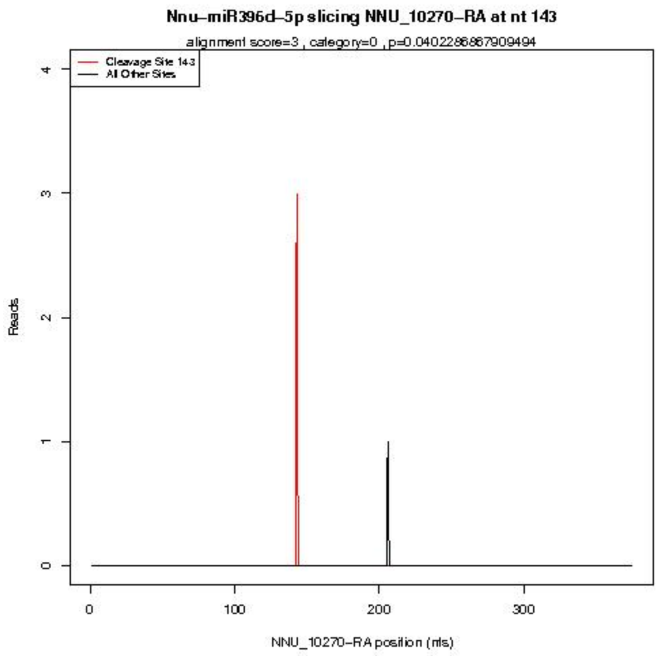
Nnu–miR396d–5p slicing NNU_10270–RA at nt 143
alignment score=3 , category=0 , p=0.0402286867909494
Cleavage Site 143
All Other Sites
Reads
NNU_10270–RA position (nts)

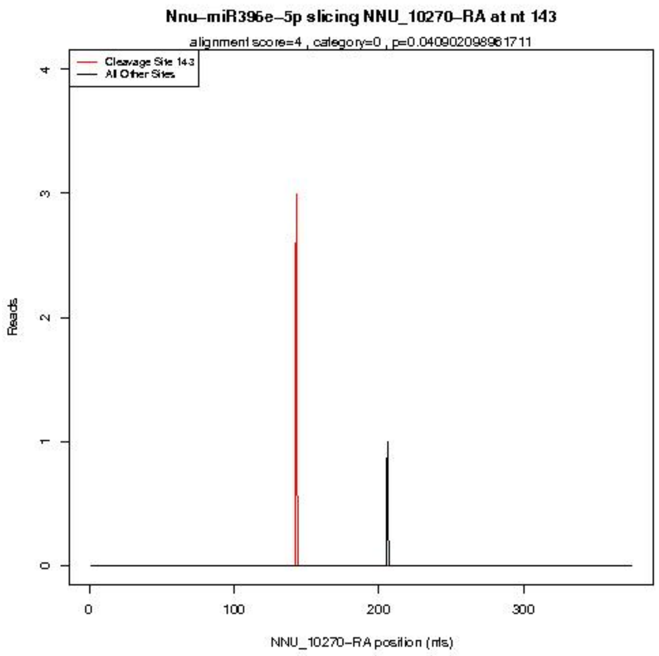
Nnu–miR396e–5p slicing NNU_10270–RA at nt 143
alignment score=4 , category=0 , p=0.040902098961711
Cleavage Site 143
All Other Sites
Reads
NNU_10270–RA position (nts)

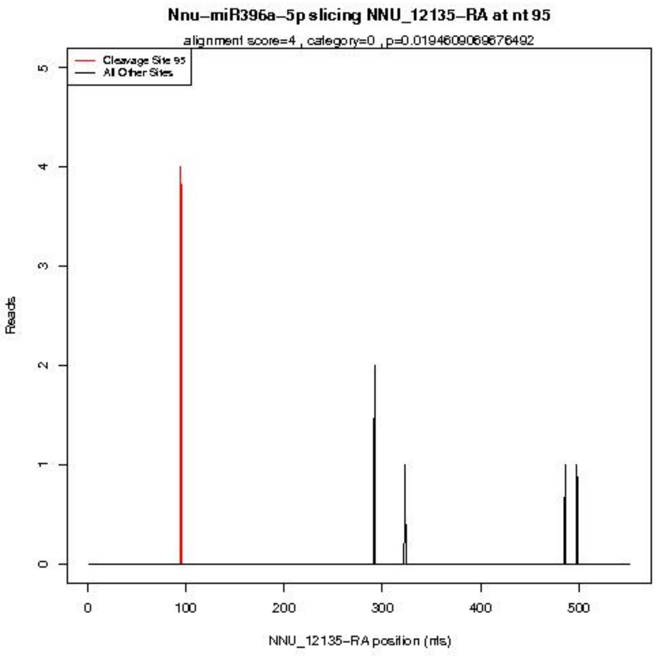
Nnu–miR396a–5p slicing NNU_12135–RA at nt 95
alignment score=4 , category=0 , p=0.0194609069676492
Cleavage Site 95
All Other Sites
Reads
NNU_12135–RA position (nts)

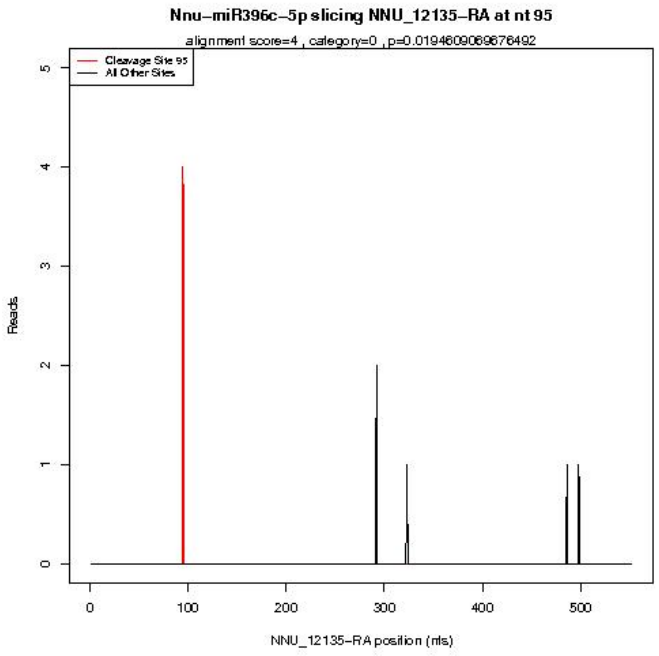
Nnu–miR396c–5p slicing NNU_12135–RA at nt 95
alignment score=4 , category=0 , p=0.0194609069676492
Cleavage Site 95
All Other Sites
Reads
NNU_12135–RA position (nts)

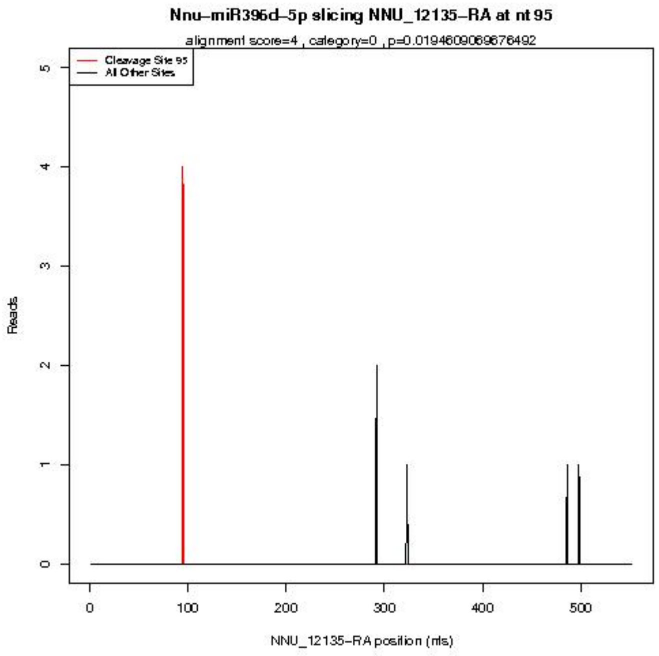
Nnu–miR396d–5p slicing NNU_12135–RA at nt 95
alignment score=4 , category=0 , p=0.0194609069676492
Cleavage Site 95
All Other Sites
Reads
NNU_12135–RA position (nts)

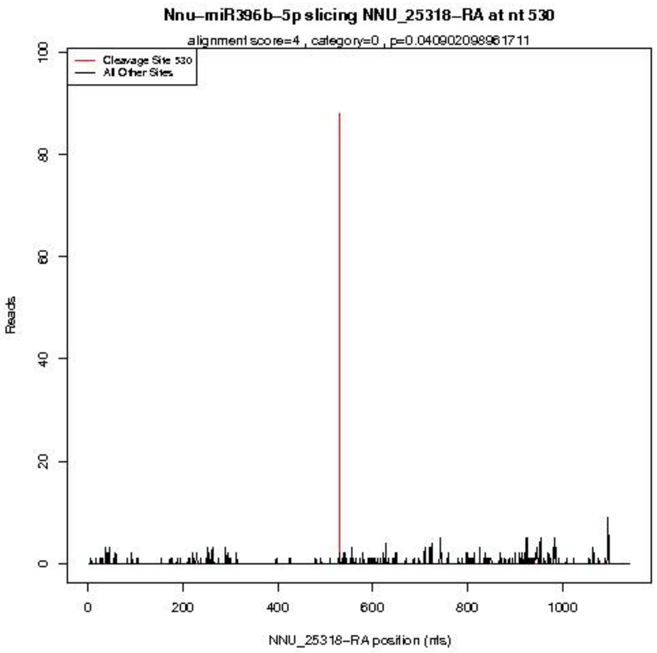
Nnu–miR396b–5p slicing NNU_25318–RA at nt 530
alignment score=4 , category=0 , p=0.040902098961711
Cleavage Site 530
All Other Sites
Reads
NNU_25318–RA position (nts)

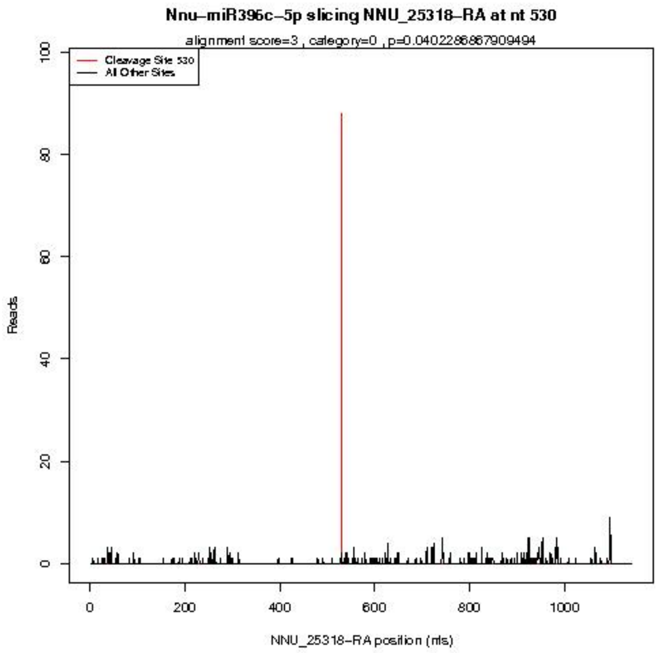
Nnu–miR396c–5p slicing NNU_25318–RA at nt 530
alignment score=3 , category=0 , p=0.0402286867909494
Cleavage Site 530
All Other Sites
Reads
NNU_25318–RA position (nts)

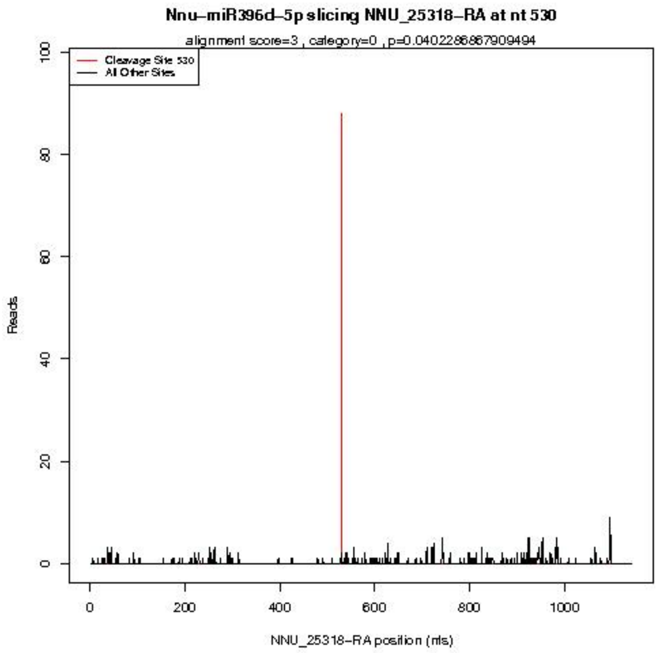
Nnu–miR396d–5p slicing NNU_25318–RA at nt 530
alignment score=3 , category=0 , p=0.0402286867909494
Cleavage Site 530
All Other Sites
Reads
NNU_25318–RA position (nts)

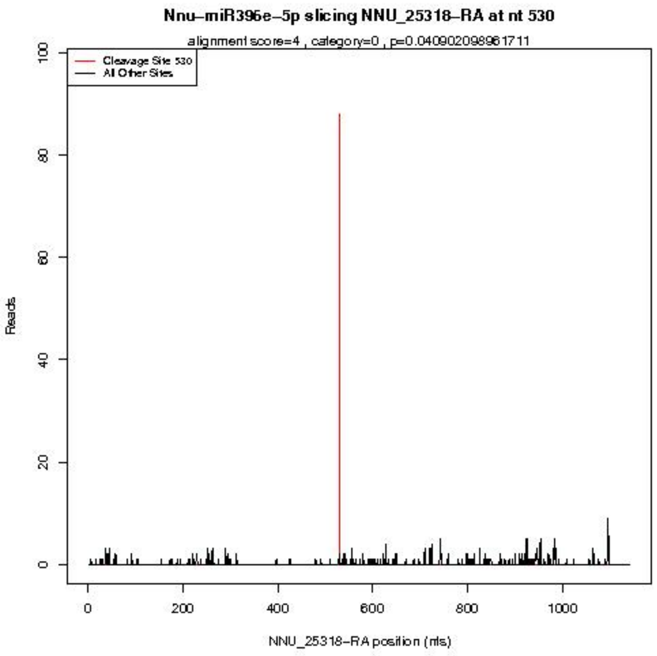
Nnu-miR396e-5p slicing NNU_25318-RA at nt 530
alignment score=4 , category=0 , p=0.040902098961711
Cleavage Site 530
All Other Sites
Reads
NNU_25318-RA position (nts)
0
20
40
60
80
100
0
200
400
600
800
1000

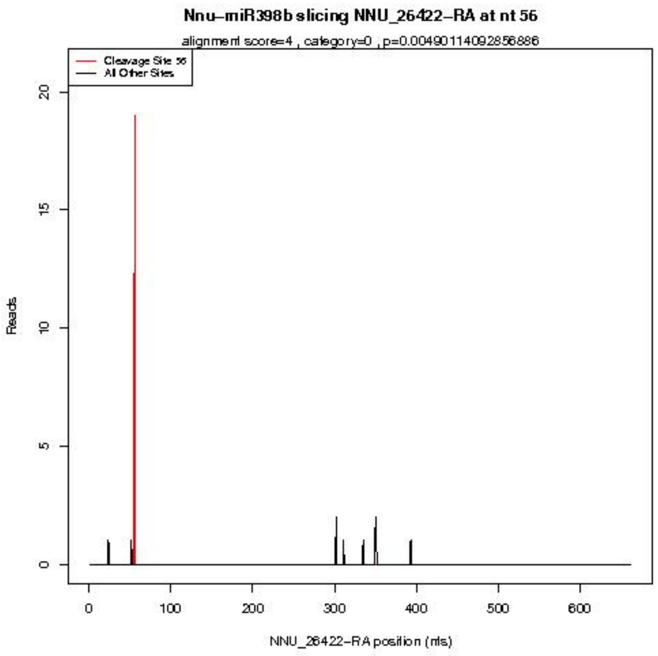
Nnu-miR398b slicing NNU_26422-RA at nt 56
alignment score=4 , category=0 , p=0.00490114092856886
Cleavage Site 56
All Other Sites
Reads
NNU_26422-RA position (nts)
0
5
10
15
20
0
100
200
300
400
500
600

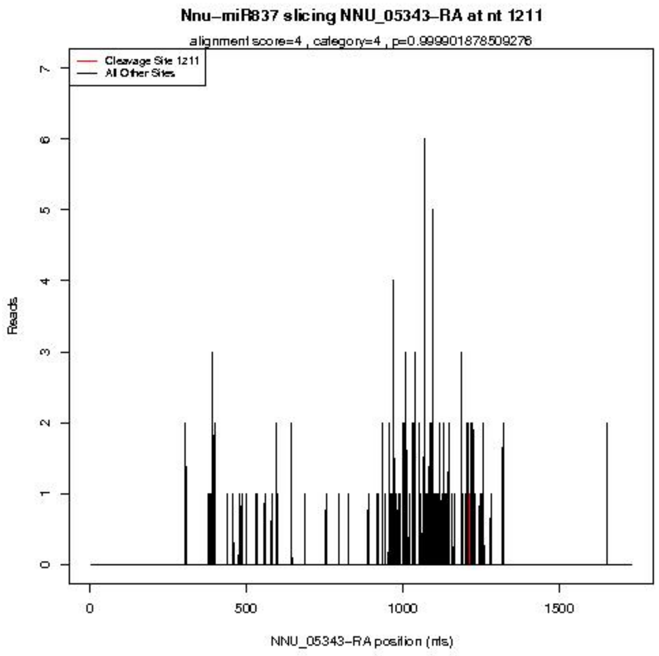
Nnu-miR837 slicing NNU_05343-RA at nt 1211
alignment score=4 , category=4 , p=0.999901878509276
Cleavage Site 1211
All Other Sites
Reads
NNU_05343-RA position (nts)
0
1
2
3
4
5
6
7
0
500
1000
1500

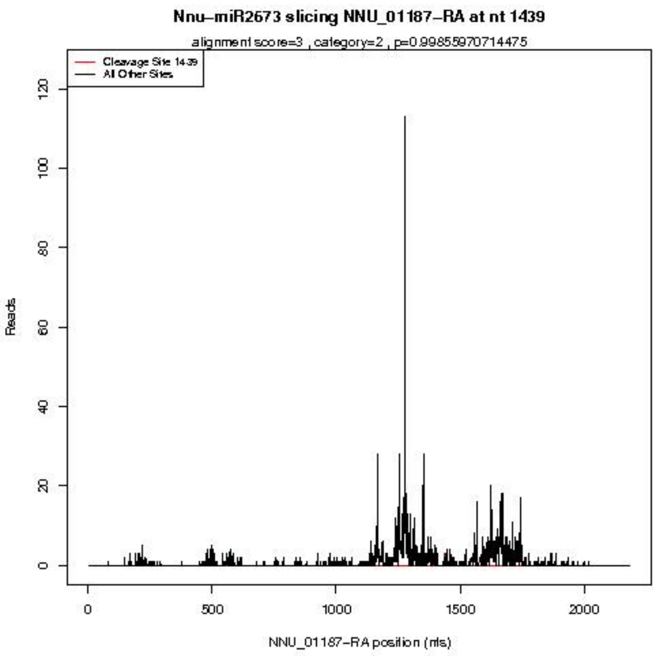
Nnu-miR2673 slicing NNU_01187-RA at nt 1439
alignment score=3 , category=2 , p=0.998559970714475
Cleavage Site 1439
All Other Sites
Reads
NNU_01187-RA position (nts)
0
20
40
60
80
100
120
0
500
1000
1500
2000

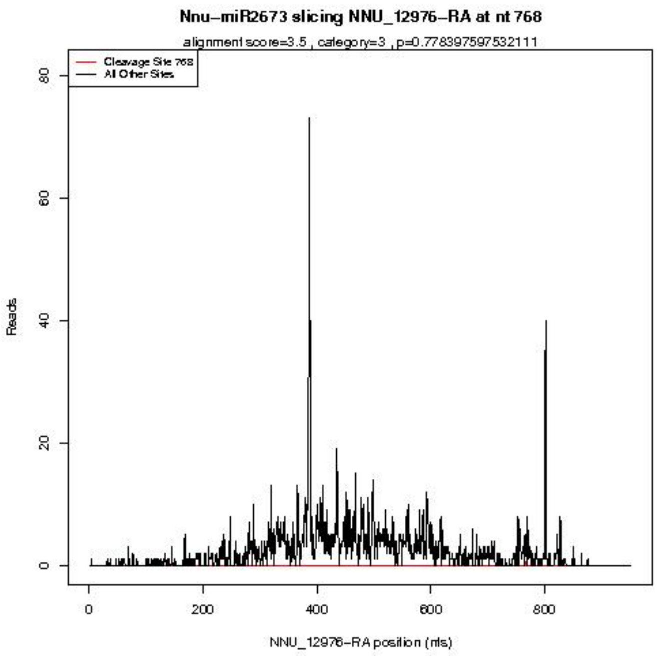
Nnu-miR2673 slicing NNU_12976-RA at nt 768
alignment score=3.5 , category=3 , p=0.778397597532111
Cleavage Site 768
All Other Sites
Reads
NNU_12976-RA position (nts)
0
20
40
60
80
0
200
400
600
800

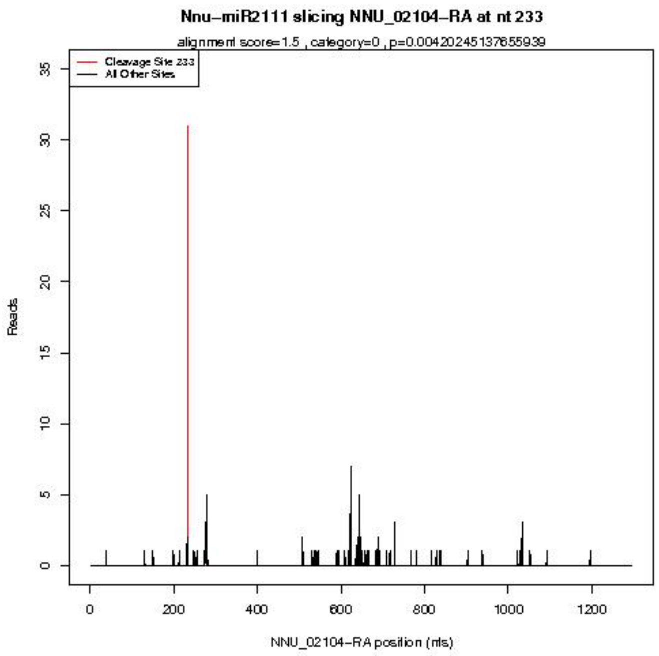
Nnu-miR2111 slicing NNU_02104-RA at nt 233
alignment score=1.5 , category=0 , p=0.00420245137655939
Cleavage Site 233
All Other Sites
Reads
NNU_02104-RA position (nts)
0
5
10
15
20
25
30
35
0
200
400
600
800
1000
1200

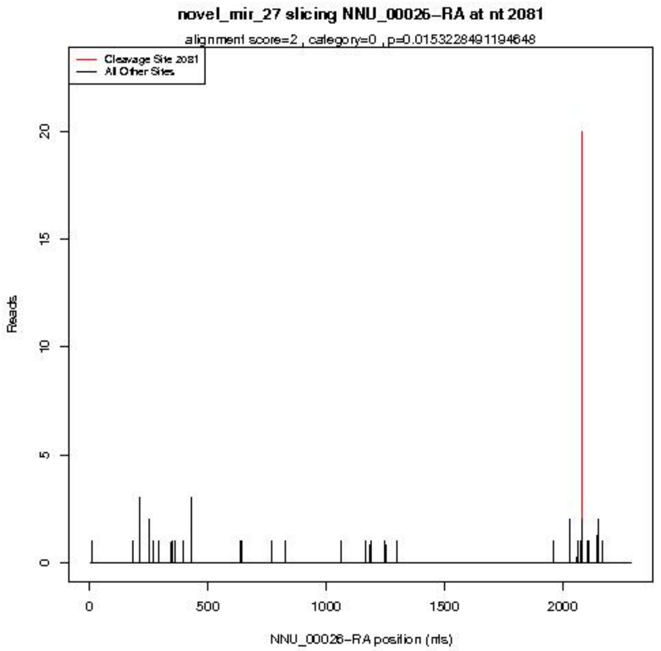
novel_mir_27 slicing NNU_00026-RA at nt 2081
alignment score=2 , category=0 , p=0.0153228491194648
Cleavage Site 2081
All Other Sites
Reads
NNU_00026-RA position (nts)

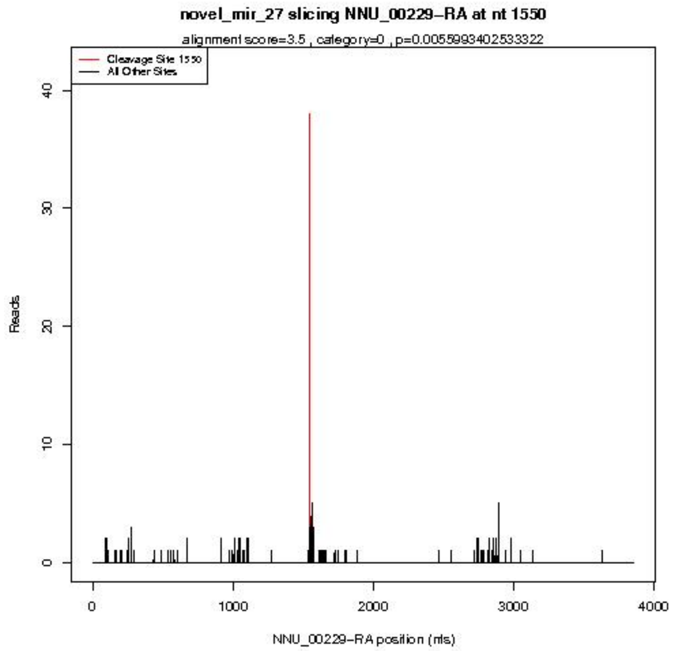
novel_mir_27 slicing NNU_00229-RA at nt 1550
alignment score=3.5 , category=0 , p=0.0055993402533322
Cleavage Site 1550
All Other Sites
Reads
NNU_00229-RA position (nts)

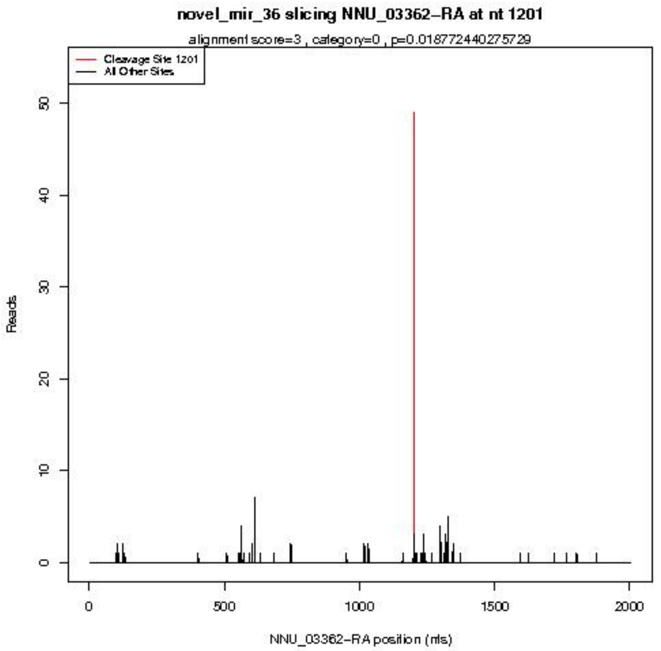
novel_mir_36 slicing NNU_03362-RA at nt 1201
alignment score=3 , category=0 , p=0.018772440275729
Cleavage Site 1201
All Other Sites
Reads
NNU_03362-RA position (nts)

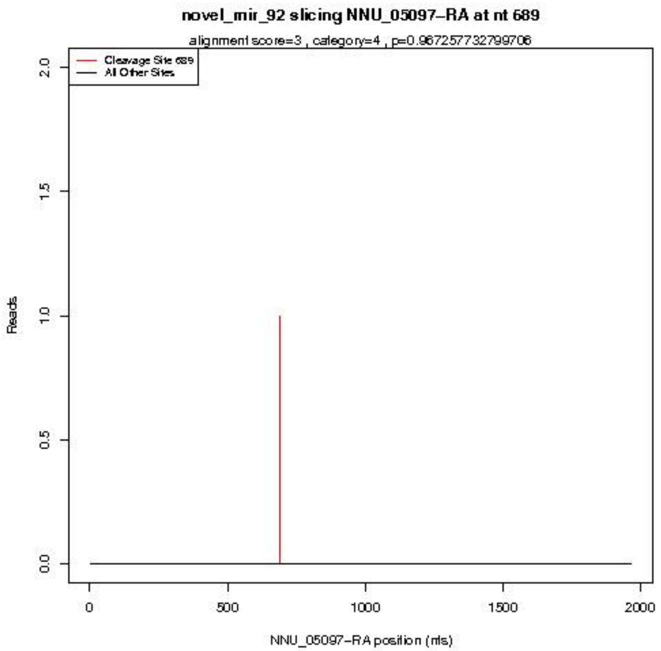
novel_mir_92 slicing NNU_05097-RA at nt 689
alignment score=3 , category=4 , p=0.967257732799706
Cleavage Site 689
All Other Sites
Reads
NNU_05097-RA position (nts)

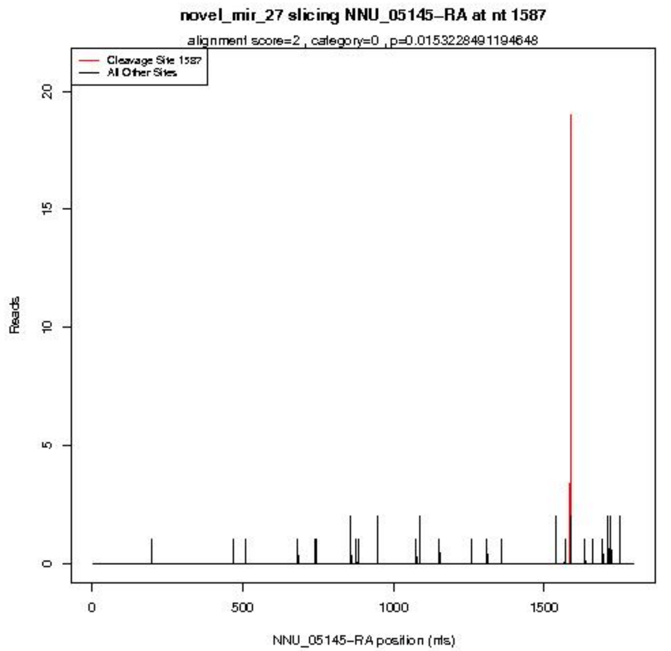
novel_mir_27 slicing NNU_05145-RA at nt 1587
alignment score=2 , category=0 , p=0.0153228491194648
Cleavage Site 1587
All Other Sites
Reads
NNU_05145-RA position (nts)

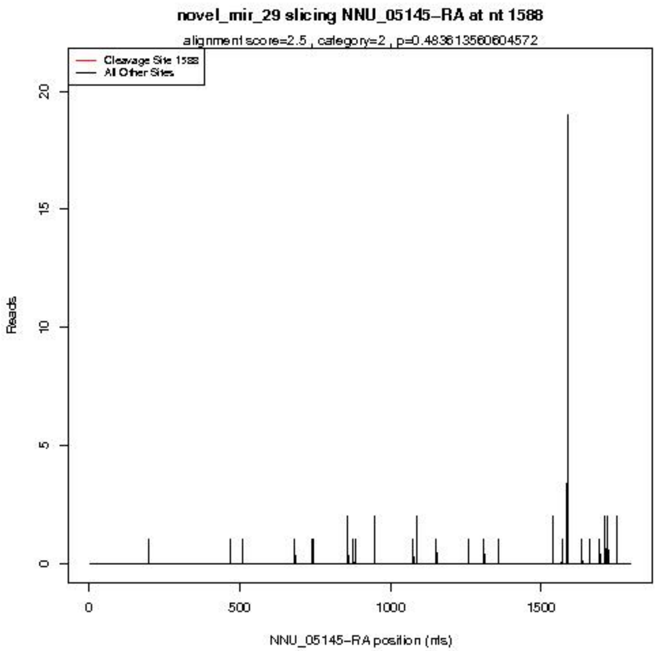
novel_mir_29 slicing NNU_05145-RA at nt 1588
alignment score=2.5 , category=2 , p=0.483613560604572
Cleavage Site 1588
All Other Sites
Reads
NNU_05145-RA position (nts)

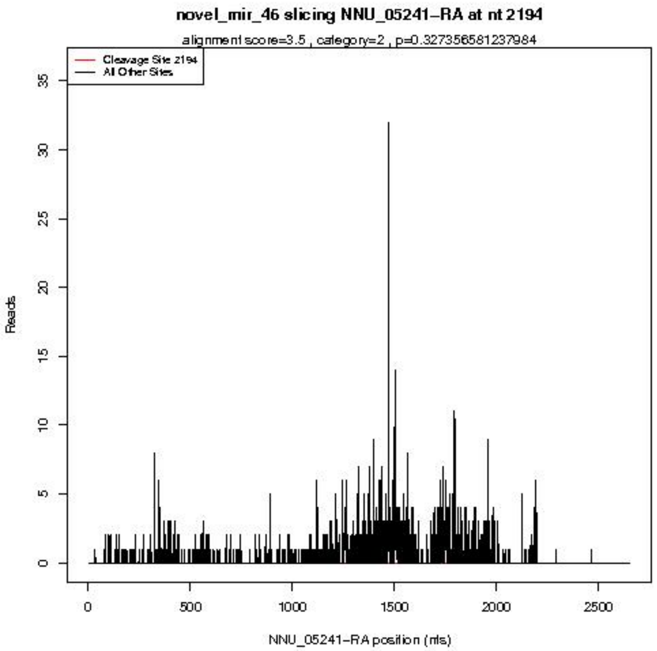
novel_mir_46 slicing NNU_05241-RA at nt 2194
alignment score=3.5 , category=2 , p=0.327356581237984
Cleavage Site 2194
All Other Sites
Reads
NNU_05241-RA position (nts)

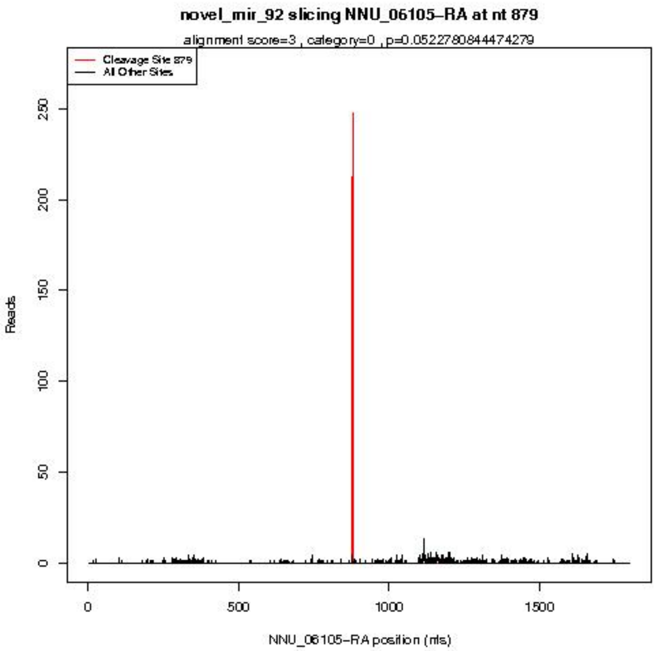
novel_mir_92 slicing NNU_06105-RA at nt 879
alignment score=3 , category=0 , p=0.0522780844474279
Cleavage Site 879
All Other Sites
Reads
NNU_06105-RA position (nts)

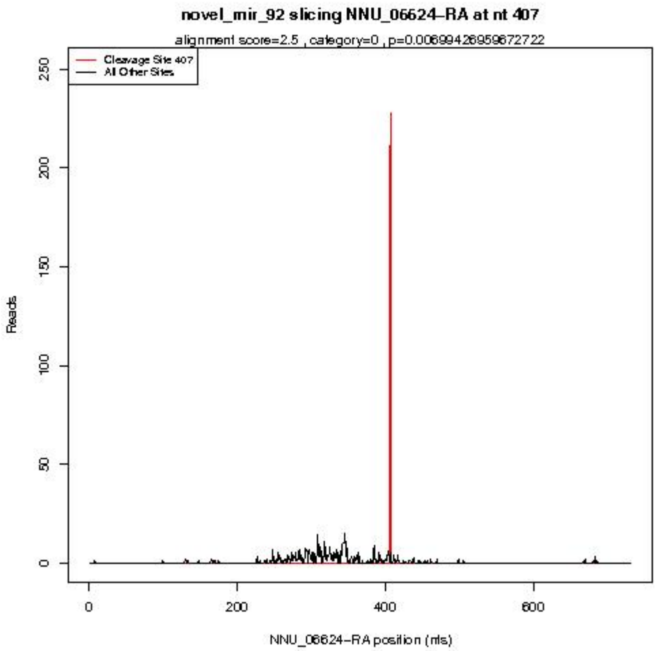
novel_mir_92 slicing NNU_06624-RA at nt 407
alignment score=2.5 , category=0 , p=0.00699426959672722
Cleavage Site 407
All Other Sites
Reads
NNU_06624-RA position (nts)

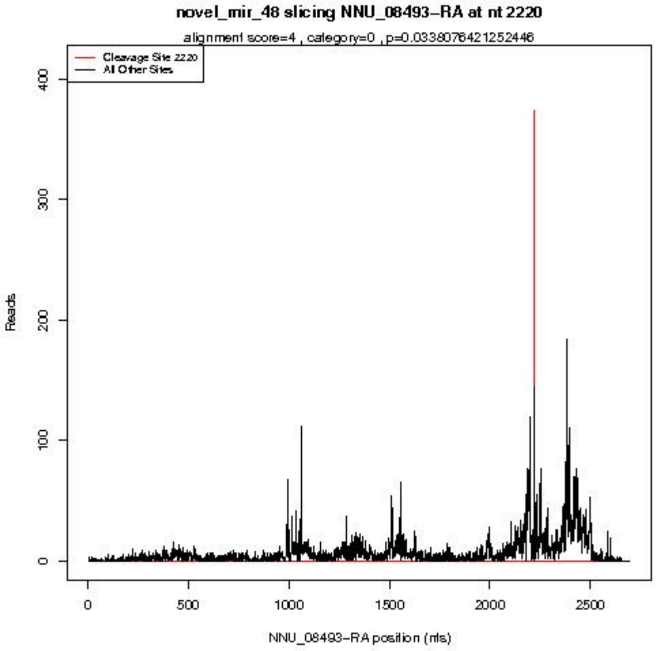
novel_mir_48 slicing NNU_08493-RA at nt 2220
alignment score=4 , category=0 , p=0.0338076421252446
Cleavage Site 2220
All Other Sites
Reads
NNU_08493-RA position (nts)

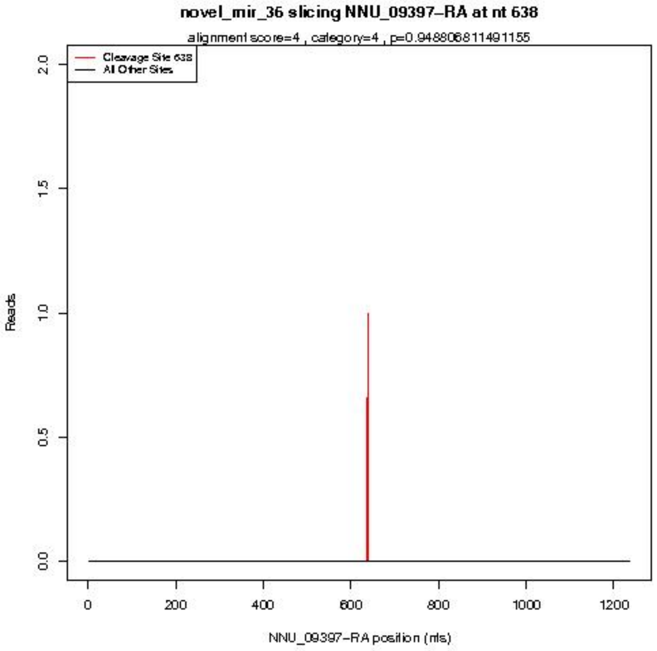
novel_mir_36 slicing NNU_09397-RA at nt 638
alignment score=4 , category=4 , p=0.948806811491155
Cleavage Site 638
All Other Sites
Reads
NNU_09397-RA position (nts)

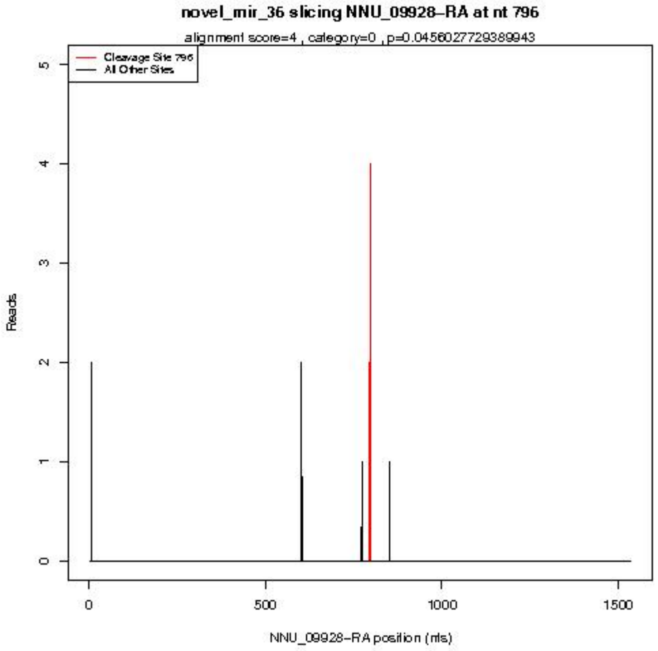
novel_mir_36 slicing NNU_09928-RA at nt 796
alignment score=4 , category=0 , p=0.0456027729389943
Cleavage Site 796
All Other Sites
Reads
NNU_09928-RA position (nts)

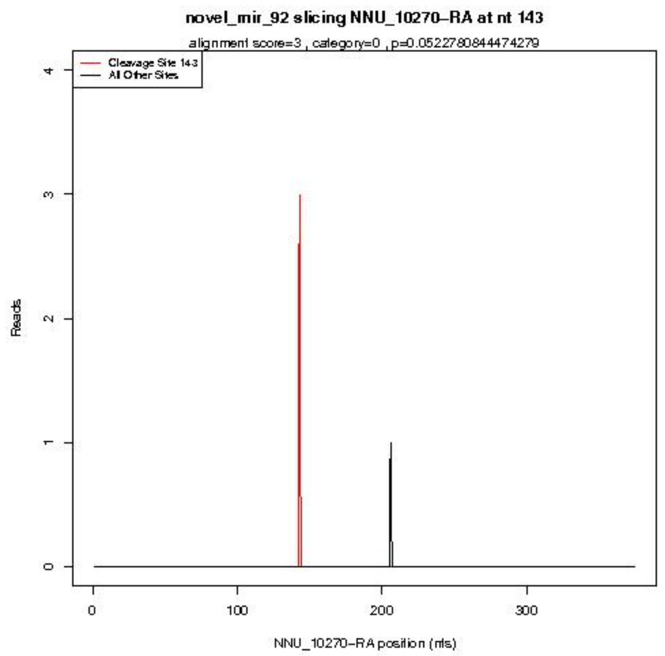
novel_mir_92 slicing NNU_10270-RA at nt 143
alignment score=3 , category=0 , p=0.0522780844474279
Cleavage Site 143
All Other Sites
Reads
NNU_10270-RA position (nts)

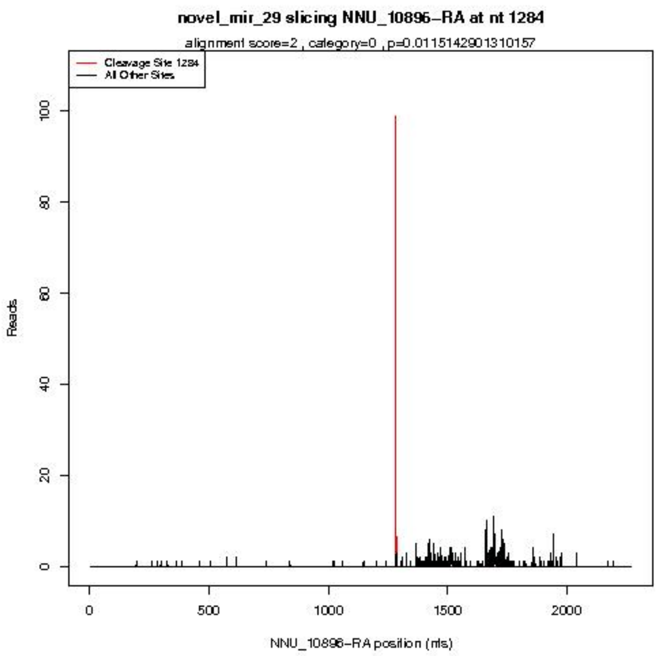
novel_mir_29 slicing NNU_10896-RA at nt 1284
alignment score=2 , category=0 , p=0.0115142901310157
Cleavage Site 1284
All Other Sites
Reads
NNU_10896-RA position (nts)

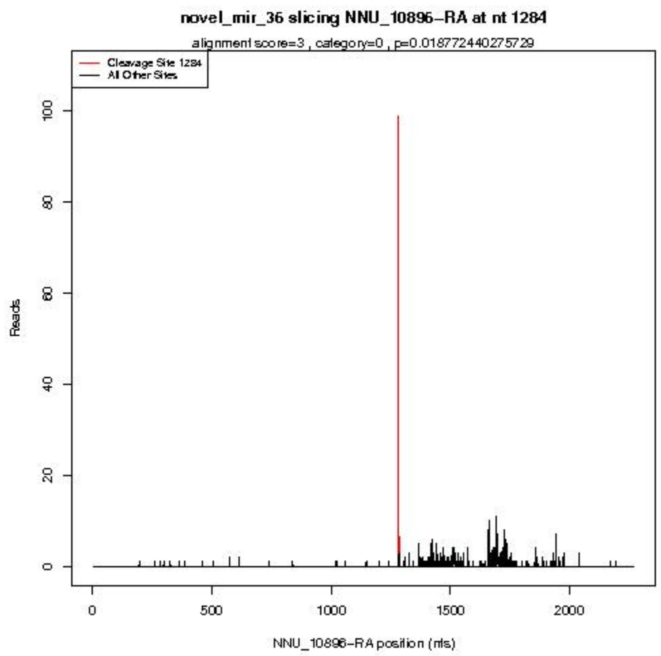
novel_mir_36 slicing NNU_10896-RA at nt 1284
alignment score=3 , category=0 , p=0.018772440275729
Cleavage Site 1284
All Other Sites
Reads
NNU_10896-RA position (nts)

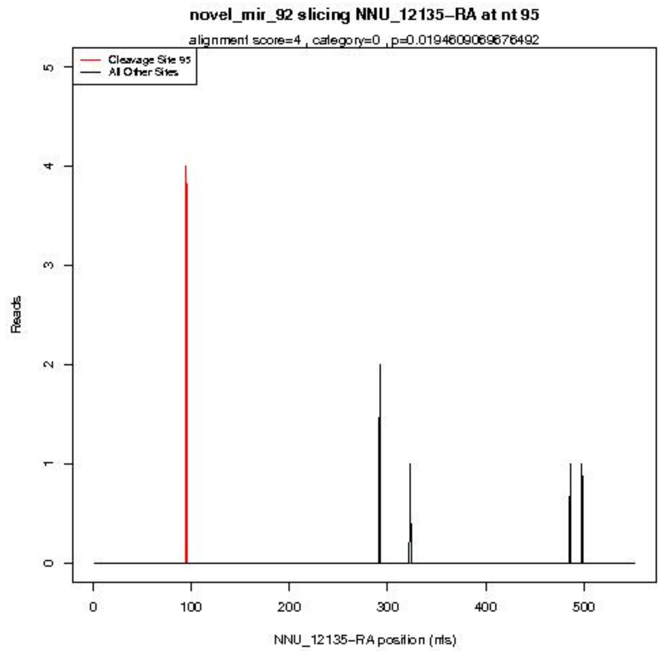
novel_mir_92 slicing NNU_12135-RA at nt 95
alignment score=4 , category=0 , p=0.0194609069676492
Cleavage Site 95
All Other Sites
Reads
NNU_12135-RA position (nts)

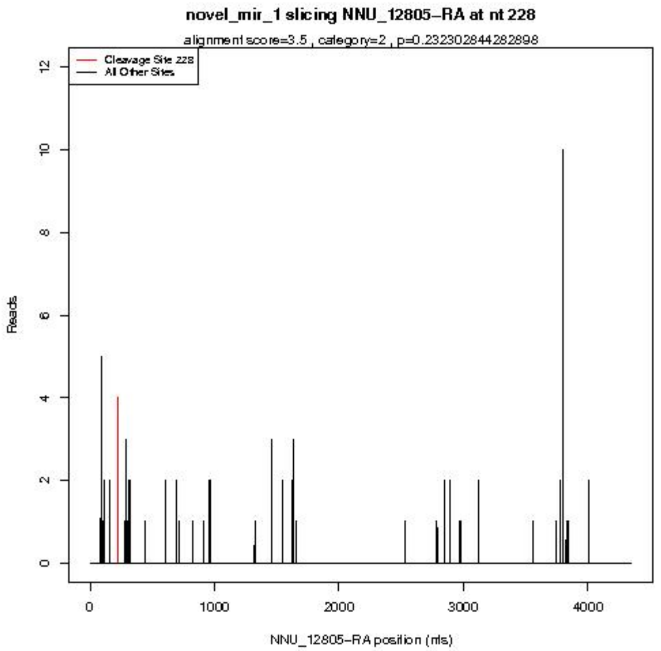
novel_mir_1 slicing NNU_12805-RA at nt 228
alignment score=3.5 , category=2 , p=0.232302844282898
Cleavage Site 228
All Other Sites
Reads
NNU_12805-RA position (nts)

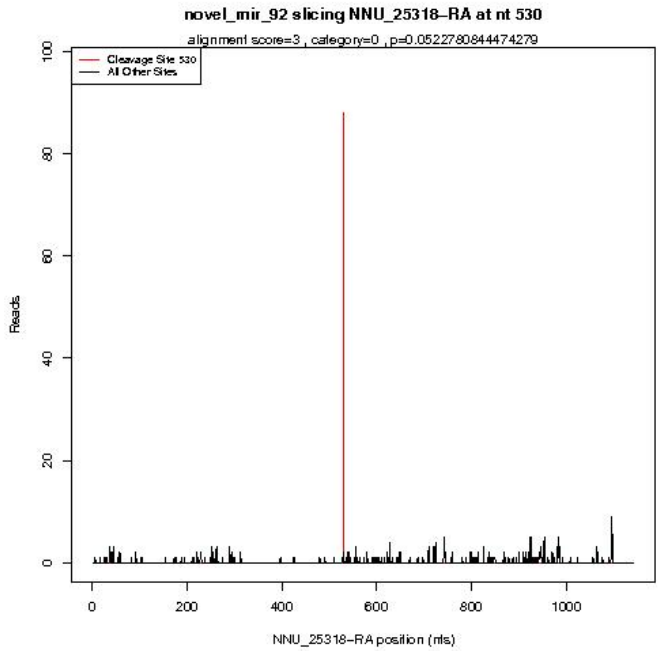
novel_mir_92 slicing NNU_25318-RA at nt 530
alignment score=3 , category=0 , p=0.0522780844474279
Cleavage Site 530
All Other Sites
Reads
NNU_25318-RA position (nts)

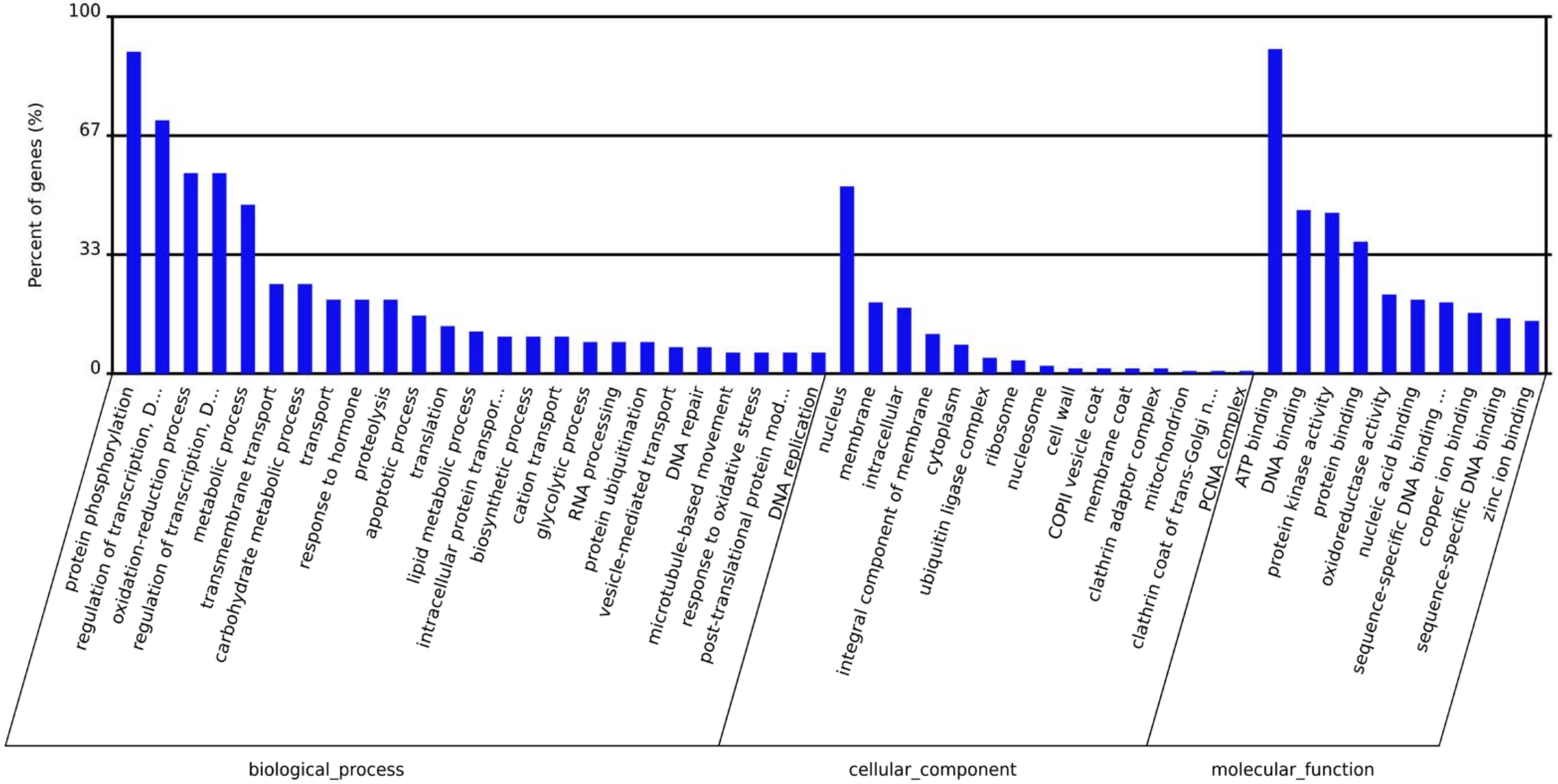

Additional file 1: Figure S5 GO classification of target transcripts for all identified miRNAs in sacred lotus.

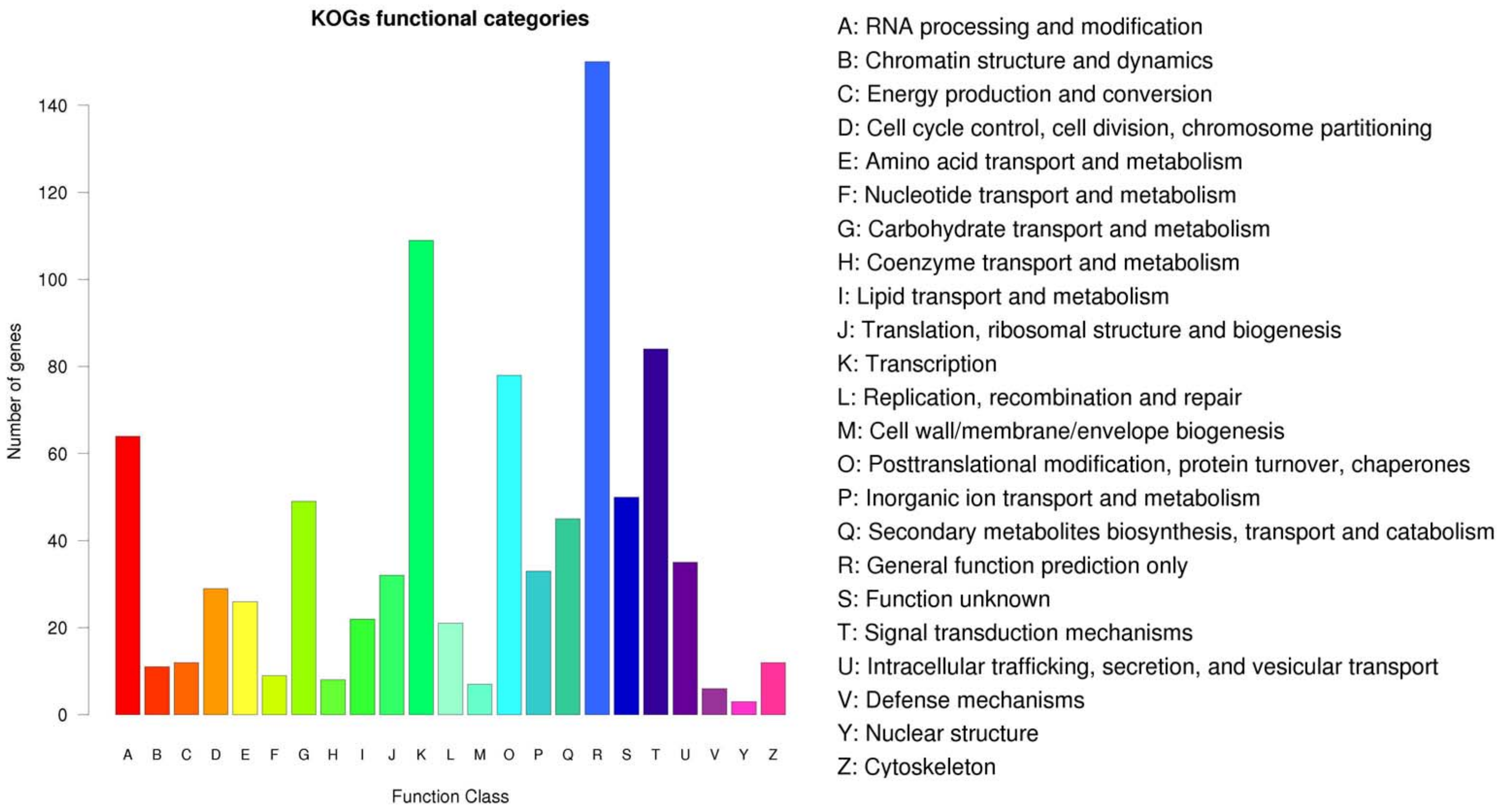

Additional file 1: Figure S6 KOG function classification of the miRNA targets in sacred lotus.
